# Consistent activation differences versus differences in consistent activation: Evaluating meta-analytic contrasts

**DOI:** 10.1162/imag_a_00358

**Published:** 2024-11-08

**Authors:** Vincent Küppers, Edna C. Cieslik, Lennart Frahm, Felix Hoffstaedter, Simon B. Eickhoff, Robert Langner, Veronika I. Müller

**Affiliations:** Institute of Neuroscience and Medicine, Brain and Behaviour (INM-7), Research Center Jülich, Jülich, Germany; Department of Nuclear Medicine, University Hospital and Medical Faculty, University of Cologne, Cologne, Germany; Institute of Systems Neuroscience, Medical Faculty and University Hospital Düsseldorf, Heinrich Heine University Düsseldorf, Düsseldorf, Germany; Department of Psychiatry, Psychotherapy and Psychosomatics, School of Medicine, RWTH Aachen University, Aachen, Germany

**Keywords:** activation likelihood estimation, neuroimaging meta-analysis, method evaluation, replicability, research synthesis, robust comparisons

## Abstract

Meta-analytic contrasts are a promising aspect of coordinate-based meta-analyses in neuroimaging research as they facilitate the statistical comparison of two meta-analytic results. They have been used for a multitude of comparisons, such as task conditions, cognitive processes, and groups. However, it remains to be tested how the results of meta-analytic contrasts relate to those of classic meta-analyses and vice versa. Here, we present a comprehensive empirical investigation of this issue using four datasets from different cognitive domains: working memory, working memory load, cognitive interference processing, and emotional face processing. For all four datasets, we compared the results of a standard meta-analysis across prototypical contrasts (condition A > condition B) reported in individual experiments with those of a contrast between two individual meta-analyses of the same conditions (meta-analysis condition A > meta-analysis condition B). In the meta-analytic contrasts, similar brain regions as in the standard meta-analysis were found but with relatively distinct spatial activation patterns. Additionally, fewer regions were revealed in the meta-analytic contrasts, especially in areas where the conditions spatially overlapped. This can be ascribed to the loss of information on the strength of activations in meta-analytic contrasts, across which standard meta-analysis summarize. In one dataset, additional regions were found in the meta-analytic contrast, potentially due to task effects. Our results demonstrate that meta-analytic contrasts can yield similar results to standard meta-analyses but are sparser. This confirms the overall validity, but also limited ability to capture all regions found in standard meta-analyses. Notable differences observed in some cases indicate that such contrasts cannot be taken as an easy substitute for classic meta-analyses of experiment-level contrasts, warranting further research into the boundary conditions for agreement.

## 1 Introduction

The neuroimaging literature of the last three decades has provided a wealth of findings on structural and functional brain–behavior relationships as well as brain-related alterations in certain diseases. Meta-analytic approaches to neuroimaging results, such as the widely used Activation Likelihood Estimation (ALE) algorithm (Eickhoff et al., 2012;Turkeltaub et al., 2002), are important tools to consolidate these findings and to overcome problems of individual studies (Müller, Cieslik, et al., 2018). Beyond testing for brain regions consistently found across studies, meta-analyses also provide the possibility to directly and statistically compare the results of two individual meta-analyses, using conjunctions and meta-analytic contrasts (Eickhoff et al., 2011;Laird et al., 2005). While conjunctions assess overlap in convergence, contrast analyses on the meta-analytic level reveal those voxels of the brain in which convergence in one meta-analysis (e.g., across studies on face processing) is significantly stronger than in another (e.g., across studies on working memory). Meta-analytic contrasts, therefore, provide a possibility to statistically compare aggregated neuroimaging data and, since their introduction, have enjoyed great popularity for comparing different experimental conditions (Owen et al., 2005;Swick et al., 2011;Wesley & Bickel, 2014), mental processes (Caspers et al., 2010;Gan et al., 2022;Langner et al., 2018), or different groups (Costa et al., 2021;Fehlbaum et al., 2022;Hill et al., 2014;Stevens & Hamann, 2012).

Meta-analytic contrasts are particularly useful to examine previously untested (or poorly studied) differences.Kogler et al. (2015), for example, reported similarities and differences between psychological and physiological stress that have not been tested before in single fMRI studies and mainly found, contrary to previous assumptions, differential convergence for both types of stress.Rottschy et al. (2012)assessed differences in working memory based on stimulus material, task load, and paradigms and reported regional specific convergence within the left dorsolateral prefrontal cortex. Importantly, meta-analytic contrasts can also help to break down larger cognitive concepts into their subcomponents. For example,Kogler et al. (2020)found supporting evidence for a multidimensional concept of empathy,Zhang et al. (2021)for the three subcomponents of executive functions, andLangner et al. (2018)for differences between top–down emotion and action regulation, which they confirmed through additional analyses.

In addition, meta-analytic contrasts are also applied to test (previously not investigated) group differences, such as different ages (Fehlbaum et al., 2022;Yaple et al., 2019;Zhang et al., 2021) or clinical groups (Costa et al., 2021;Klugah-Brown et al., 2021). This is an important potential of contrasts on the meta-analytic level as studies comparing different clinical groups to each other as well as to a control group are quite costly.

These examples illustrate some of the many uses of meta-analytic comparisons. Especially, as the number of neuroimaging results continues to increase, many more applications of meta-analytic comparisons become possible. While all of them can theoretically also be carried out in individual fMRI studies, meta-analytic contrasts take advantage of the wealth of already conducted experiments by looking at them from a different angle and are a promising exploratory method for hypothesis generation.

However, while meta-analytic contrasts offer new possibilities for investigating different concepts, it should be noted that the results of meta-analytic contrasts may not necessarily reflect differences in brain activation, despite often being interpreted as such. Classic ALE meta-analyses are conducted across coordinates derived from individual neuroimaging studies, which typically reflect differences in activation strength between two different brain states or participant groups. The results, thus, reflect the convergence (i.e., consistency) of brain activation differences reported across these studies (Eickhoff et al., 2012). ALE meta-analytic contrasts, on the other hand, compare the results of two classic meta-analyses, testing for those voxels where convergence (the ALE value) significantly differs between two meta-analysis. Meta-analytic contrast analysis, thus, adds an additional layer on top of classic ALE analyses and is an important tool for interpretation as it provides formal information if brain regions found in one meta-analysis but not in the other truly differ in their ALE scores.

It might be suggested that meta-analytic contrasts and classic meta-analyses of the same comparison pick up similar mechanisms. However, as can be seen from the description of the calculation above, meta-analytic contrasts differ conceptually. While meta-analytic contrasts investigate differences in convergence without considering activation strength, classic meta-analyses test for convergence across activation strength. This distinction is further highlighted by two previous clinical meta-analyses using both approaches to investigate changes in brain activation in autism (Costa et al., 2021) and borderline personality disorder (Degasperi et al., 2021) compared with healthy controls. Both studies found divergent results for the two approaches.Costa et al. (2021), for example, identified regions of consistent aberrant brain activation computing a classic meta-analysis (across experiments of patients vs. controls). However, a meta-analytic contrast of the same but conceptually different comparison (meta-analysis across patients vs. meta-analysis across controls) did not. The authors argue that differences in the magnitude of activation can be obscured when looking at differences in convergence (i.e., meta-analytic contrasts). However, while highlighting the importance to distinguish between the two approaches, previous results can only be generalized to group comparisons. Importantly, clinical meta-analyses often consolidate findings found for a specific patient group independent of a particular process and are therefore quite heterogeneous in the tasks and processes investigated (Müller et al., 2017), leading to overall less convergence for all analyses. Results reported byCosta et al. (2021)andDegasperi et al. (2021)may, therefore, be affected by this heterogeneity and may not be transferable to meta-analyses that investigate different task conditions.

Thus, the interpretation of meta-analytic contrast results and their ability to capture effects observed at the experimental level are still not fully clarified. The increasing popularity in computing these contrasts and the resulting interest in understanding the findings necessitates a systemic investigation.

The current study aimed to provide deeper insights into the interpretability and validity of meta-analytic contrasts by conducting a comprehensive empirical evaluation. We compared the results of meta-analytic contrasts with the results of individual meta-analyses across experimental comparisons. This approach is similar toCosta et al. (2021)andDegasperi et al. (2021), who computed group comparisons (patients > controls; meta-analysis patients > meta-analysis controls). However, in order to keep the complexity low and to avoid additional confounding effects at the level of group comparisons, we took a step back and focused on comparing prototypical task conditions in three different cognitive domains. Thus, in each domain, we compared the results of a meta-analysis across contrasts between two conditions (condition A > condition B) with the results of a meta-analytic contrast that contrasted two meta-analyses of the same two conditions with each other (meta-analysis condition A > meta-analysis conditions B).

## 2 Methods

In this paper, the standard meta-analysis across experiment-level contrasts is abbreviated to*CM*(contrast-meta); the meta-analytic contrast between two meta-analyses is abbreviated to*MC*(meta-contrast). It is important to note that the input-data (coordinates we obtained from the individual neuroimaging studies) required for the computation of the two approaches is different. In the first case (CM), coordinates are needed for the contrast between*condition A*>*condition B*, whereas in the second case (MC), the main effect coordinates are needed for*condition A*>*baseline*and coordinates for*condition B*>*baseline*. The availability of the coordinates determines which method can be used.

To conduct a comprehensive empirical study, we collected four sets of data from different cognitive domains.*Working memory 2-back > 0-back*: based on the n-back task, the 2-back (condition A) was contrasted with the 0-back (condition B) condition. To compute the contrast-meta, experiments reporting 2-back > 0-back (A>B) were collected. For the meta-contrast, experiments reporting 2-back > baseline (A) and 0-back > baseline (B) were separately collected, two individual meta-analyses (meta-analysis across A and one across B) computed, and the results statistically compared in the meta-contrast.*Working memory 2-back > 1-back*: experiments reporting 2-back > 1-back (A>B) for the CM and 2-back > 0-back (A) and 1-back > 0-back (B) for the MC were collected.*Cognitive interference processing*: experiments of the color-word Stroop task were collected. Incongruent > congruent (A>B) experiments were collected for the CM, whereas incongruent > control/baseline (A) and congruent > control/baseline (B) experiments were gathered for the MC.*Emotional face processing*: experiments across different tasks using emotional and neutral face stimuli were collected. Experiments of the emotional > neutral faces (A>B) comparison were used for the CM. Emotional faces > control/baseline (A) and neutral faces > control/baseline (B) for meta-contrast (seeTable 1).

**Table 1.** Datasets and experiments (contrasts at experimental level) included in contrast-meta (CM) and meta-contrast (MC).

Domain (dataset)	Experiments included in CM	Experiments included in MC - condition A	Experiments included in MC - condition B
Working memory (WM: 2-back > 0-back)	2-back > 0-back	2-back > baseline	0-back > baseline
Working memory load (WM: 2-back > 1-back)	2-back > 1-back	2-back > 0-back	1-back > 0-back
Cognitive interference processing (interference)	incongruent > congruent	incongruent > control; incongruent > baseline	congruent > control; congruent > baseline
Emotional face processing (emo)	emotional > neutral faces	emotional faces > control; emotional faces > baseline	neutral faces > control; neutral faces > baseline

### 2.1 Analysis approach

As described above, we used two different meta-analytic approaches for computing the same contrast (e.g.,*condition A*>*condition B*): First, an ALE meta-analysis was calculated across experiments that delineated the contrast of interest on the experimental level (CM). The CM, thus, includes experiments reporting results that compare condition A with condition B (*condition A*>*condition B*). This type of meta-analysis, thus, revealed spatial convergence of differences in brain activations between conditions across experiments (Müller, Cieslik, et al., 2018) (compareFig. 1).

**Fig. 1.**
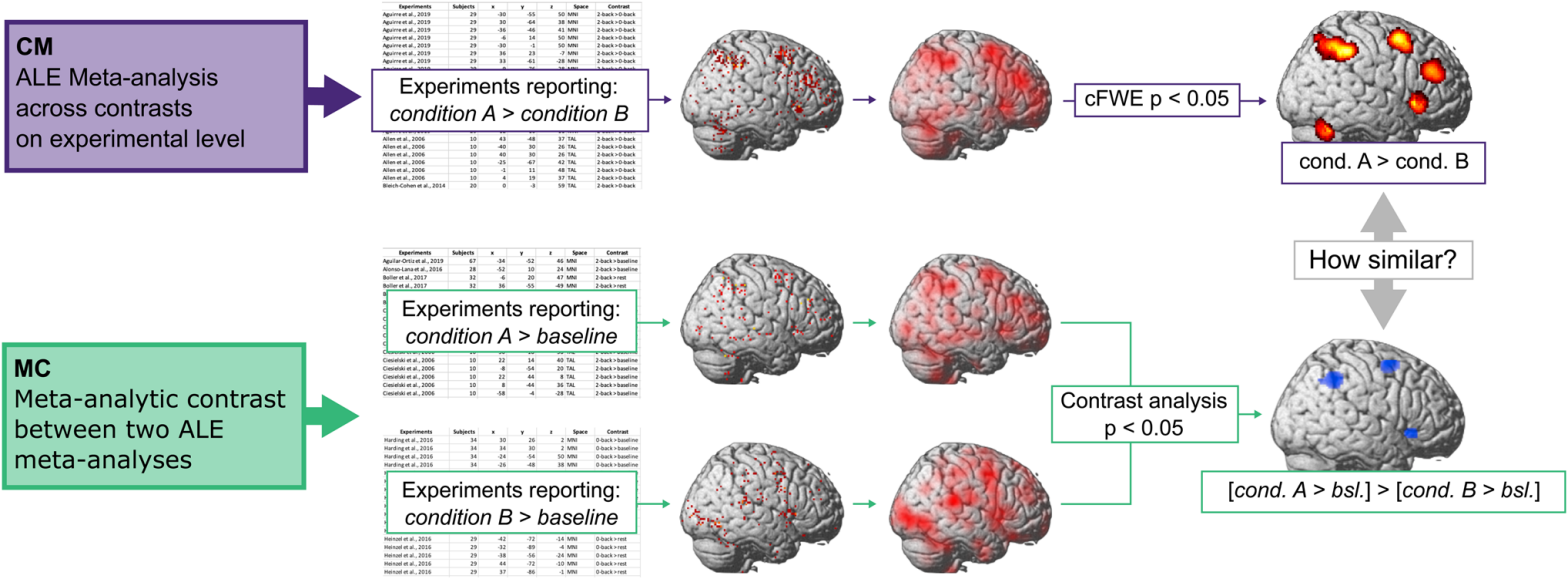
Schematic depiction of the analytic approach: Comparison of the results of a meta-analysis of experiments contrasting condition A with condition B (CM) with a meta-analytic contrast between a meta-analysis of experiments contrasting condition A with a baseline and another meta-analysis contrasting condition B with a baseline (MC).

Second, the contrast of interest was calculated by contrast analysis between the results of two meta-analyses (MC). To do so, we first calculated two separate meta-analyses, one across experiments contrasting*condition A*with a control or baseline condition and once across experiments contrasting*condition B*with a control or baseline condition. Importantly, a significant convergence of a brain region in the meta-analysis across*condition A*and a lack of convergence of the same brain region in the meta-analysis across*condition B*does not necessarily indicate that*condition A*differs in convergence from*condition B*. Thus, to formally test significant difference in convergence, the two meta-analyses were contrasted against each other using meta-analytic contrast analysis resulting in the same contrast of interest (*condition A*>*condition B*) as CM but calculated in a different way. In contrast to the CM, the MC results reflect differences in the across-study convergence of brain activation between conditions.

#### 2.1.1 Activation likelihood estimation

For each dataset, we conducted three meta-analyses: one for the meta-analysis across experimental contrasts (CM), and two for the meta-analytic contrasts (MC). All meta-analyses were computed based on the activation likelihood estimation algorithm (Eickhoff et al., 2009,^2012^;Turkeltaub et al., 2002,^2012^). Here, we used an in-house Python implementation of the algorithm (https://github.com/LenFrahm/pyALE).

In ALE, the foci of all included experiments are treated as centers of Gaussian probability distributions which captures the spatial uncertainty of the coordinates. The width of these distributions is modeled based on empirical data of between-template and between-participant variance. The between-participant variance is weighted by the number of participants of the respective study as a larger sample size is considered as spatially more reliable and therefore modeled with a denser Gaussian than experiments with a smaller sample size. For each experiment, a modeled activation map is generated by aggregating the probabilities of all reported foci for each voxel. To account for experiments reporting multiple foci close to each other, each voxel only receives the largest possible probability value from all foci close to it (Turkeltaub et al., 2012). Then, the probabilities are combined by taking the voxel-wise union across all modeled activation maps of each individual experiment. The resulting map of voxel-wise ALE scores, thus, reflects the convergence of results across experiments in each voxel of the brain (Eickhoff et al., 2009).

Next, the ALE scores are tested against a null distribution of random spatial associations to distinguish true from random convergence and results are thresholded at p < 0.001. The null distribution is calculated using an analytic procedure based on a nonlinear histogram algorithm (Eickhoff et al., 2012).

Finally, a permutation approach is used to correct for multiple comparisons (cFWE p < 0.05) by comparing the cluster sizes to a null distribution of cluster sizes. This null distribution is created by randomly distributing foci (except location all other properties of the foci are held constant) within a grey-matter mask, calculating an ALE analysis in the same manner as with the real data and recording the maximum cluster size found in this analysis. This procedure is repeated 10,000 times, resulting in an empirical null distribution of cluster sizes. A cluster is considered significant if its size exceeds the sizes of 95% of those random permutations.

#### 2.1.2 ALE meta-analytic contrasts

To calculate MCs for each dataset, we contrasted the two ALE meta-analyses across the coordinates of the main effects (*condition A*>*baseline*and*condition B*>*baseline*). The ALE contrast analysis is a statistical comparison between the results of two meta-analyses (Eickhoff et al., 2011). Here, we used an in-house implementation in Python, similar to the current implementation in BrainMap, GingerALE (Version 3.0.2). Meta-analytic contrast analyses were performed by first calculating two separate meta-analyses and calculating the voxel-wise difference score. These difference scores are then compared to an empirical null distribution of ALE difference scores under the assumption of exchangeability. This was done by pooling the experiments of both conditions, shuffling them, and randomly splitting them into two groups of the original size of experiments. ALE difference scores between these random groups are then recorded for every voxel. This process is repeated 10,000 times, and the real differences in ALE scores are tested against this null distribution of difference scores. Here, a threshold of p > 0.95 was used (i.e., the observed probability in the difference between ALE values per voxel needed to be equal to or higher than 95% chance level), the results were inclusively masked by the respective main effect of the condition of interest, and an additional cluster extent threshold of k = 5 was applied.

### 2.2 Data collection and datasets

We conducted a comprehensive literature search to construct, as mentioned above, four datasets for three distinct cognitive domains: working memory (WM), cognitive interference processing (interference), and emotional face processing (emo). These domains were selected based on their extensive investigation in the neuroimaging literature and the availability of sufficient experiments to calculate both a meta-analysis across experimental contrasts (CM) as well as a meta-analytic contrast between two meta-analyses (MC). We did not seek to obtain an ethics vote for this study as our analyses were based solely on previously published aggregated data and did not include individual participant data.

The datasets were constructed from previous meta-analyses (Cieslik et al., 2015;Langner & Eickhoff, 2013;Müller, Höhner, et al., 2018;Rottschy et al., 2012) and extended by tracing references from additional meta-analyses and conducting a comprehensive literature search using the “PubMed” (https://pubmed.ncbi.nlm.nih.gov/) and “Web of Science” (http://webofknowledge.com/) search engines. Different variations of the keywords “fMRI” and “PET” were combined with condition- and domain-specific search terms. We included studies, that reported results of at least one of the contrasts of interest (A>B, A or B), that is, reporting of all three contrasts of interest was not a requirement for inclusion since only few studies reported all contrasts. In accordance with the general guidelines (Müller, Cieslik, et al., 2018), only whole-brain comparisons (scan and analysis) from healthy adults (mean age 18 or older) that reported results in standard anatomical space (TAL or MNI) were included. We excluded intervention, treatment, or induction studies (e.g., medication, TMS). However, if a study reported results of a baseline measurement before an intervention, the corresponding contrast was included. In cases where a paper reported multiple experiments for the same condition of interest in the same group of participants, the coordinates were pooled and treated as one experiment. Results reported in one paper but obtained from different participant groups were treated as separate experiments. Additionally, we reached out to authors (Agostini et al., 2017;Aguirre et al., 2019;Campanella et al., 2013;Cullen et al., 2016;Daamen et al., 2015;Dan et al., 2019;Fukuda et al., 2019;Ghavidel et al., 2020;Habel et al., 2007;Harding et al., 2016;Jung et al., 2018;Kaminski et al., 2020;King et al., 2015;Köhler et al., 2016;Kowalczyk et al., 2021;Kozasa et al., 2018;Kronbichler et al., 2018;Krug et al., 2008;Lahr et al., 2018;Li et al., 2019;Luethi et al., 2016;Miró-Padilla et al., 2019;Papalini et al., 2019;Peven et al., 2019;Schlagenhauf et al., 2008;Schmidt et al., 2012;Schneider et al., 2007;Shashidhara et al., 2020;Smits et al., 2009;Szabó et al., 2019;Takeuchi et al., 2018;Vacchi et al., 2017;van der Horn et al., 2016;Wagner et al., 2015;Wallentin et al., 2015;Wu et al., 2017;Ye & Zhou, 2009) via email to request pertinent additional results (seeFig. 2).

**Fig. 2.**
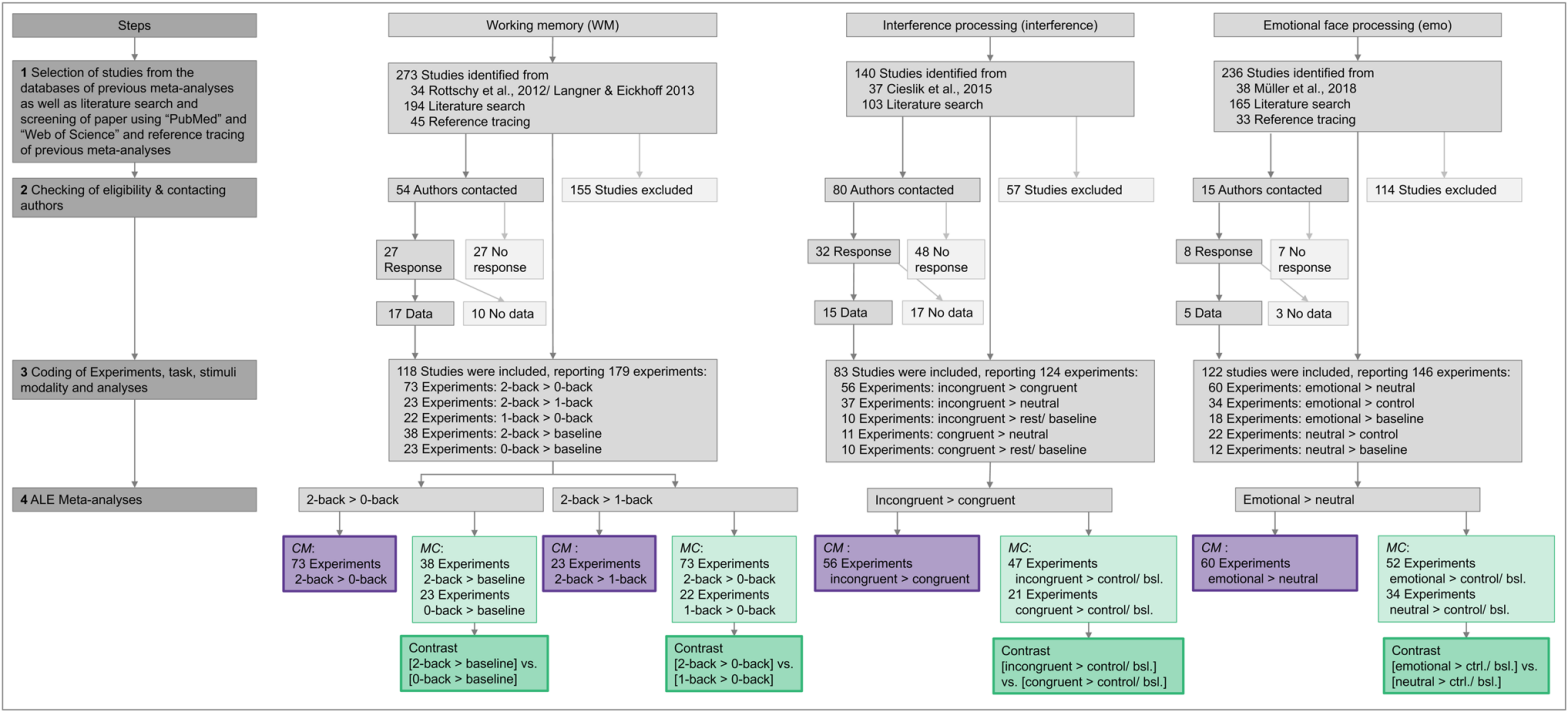
Flowchart depicting the creation of four datasets from three cognitive domains and the subsequent analyses.

Literature search and coding was performed by one author, and the resulting datasets were checked for eligibility and correctness by a second author. Literature search was performed up to 2021-12-09.

Details about the experimental designs, conditions, and specific tasks for each dataset can be found in theSupplementary Material.

In the working memory (2-back > 0-back and 2-back > 1-back) and interference processing datasets, the experiments showed a relatively high degree of homogeneity. Specifically, within the working memory domain, all experiments used the n-back task, with variations between experiments only in the stimuli used (letters, numbers, pictures, etc.). Similarly, in the interference processing domain, experiments used the color-word Stroop task, with only some variations in the stimuli (different colors, number of colors). Additionally, the contrasting condition (*> baseline*) of the experiments used for the calculation of the MC remained relatively consistent in the working memory dataset. However, in the interference processing dataset, there was some variation, with rest and control (e.g., words, symbols) as contrasting conditions.

In contrast, the emotional face processing dataset showed greater heterogeneity compared to the other two domains. This dataset encompassed experiments involving different tasks (such as gender-discrimination, passive viewing, and emotion-matching) along with a wide range of stimuli (different face datasets, different emotions, etc.). The contrasting conditions in this dataset were more diverse, including rest and control conditions with varying characteristics such as shapes, objects, scenes, and others. Further details about the four datasets can be found in theSupplementary Material(Supplementary Tables S4–S6), providing a comprehensive description of the experimental designs and conditions.

Figure 2provides a visual representation of the literature search process, including the number of authors contacted and the contrasts of interest.

#### 2.2.1 Comparison of meta-contrast and contrast-meta results

To quantify the extent to which the MC results were similar to the CM results, different metrics were applied for voxel-, cluster-, and peak-related comparisons.

##### 2.2.1.1 Voxel-wise comparison

First, we evaluated similarity based on a whole-brain voxel-wise comparison of the two result maps. First, we binarized both maps, that is, treated significant voxels as 1’s and non-significant voxels as 0’s and calculated the Jaccard similarity coefficient (Jaccard, 1901;Maitra, 2010) as well as the sensitivity and precision. Jaccard coefficient was calculated as the intersection of all significant voxels between CM and MC divided by the union of significant voxels (Eq. (1)).

$$Jaccard\;coefficient=\frac{CM\cap MC}{CM\cup MC}$$ (1)

Sensitivity was assessed by calculating the intersection of all significant voxels between CM and MC divided by all significant voxels of CM (Eq. (2)). This metric reflects how well the MC reveals voxels that are also significant in the CM analysis.

$$Sensitivity=\frac{CM\cap MC}{CM}$$ (2)

Precision, in contrast, shows the proportion of all significant MC voxels that lie within the CM network (Eq. (3)).

$$Precision=\frac{CM\cap MC}{MC}$$ (3)

##### 2.2.1.2 Cluster-wise comparison

As a second measure of similarity, a comparison of clusters was chosen, providing a similarity measure that is relatively independent of the size of clusters and more based on the spatial location of results. Here, we assess how many clusters of one map overlap with clusters from the other map. Clusters are counted as overlapping if at least one voxel of both maps is overlapping (Bossier et al., 2020). Cluster-wise sensitivity was computed by first dividing the number of CM clusters overlapping with clusters from MC by the total number of CM clusters. Precision was assessed by first dividing the number of MC clusters overlapping with clusters from CM by the total number of MC clusters.

##### 2.2.1.3 Peak-wise comparison

As the cluster-wise comparison does not take into account cases where the same anatomical region is revealed in both analyses without direct overlap, for example, in the case of relatively small clusters, similarity between meta-analytic maps was additionally assessed by assessing the location of local maxima. This was done by extracting peaks from CM and MC using the FSL cluster command on the uncorrected thresholded z-score maps. We extracted all local maxima with a minimum distance of 8 mm (default distance in SPM12—Statistical Parametric Mapping software) and evaluated the proximity to the nearest peak in the opposite map. In particular, for each local maxima from CM we calculated the Euclidean distance to the next peak from MC. Likewise, for all local maxima from MC we determined the closest peak from CM. Median distances in each direction (CM to MC and MC to CM) were computed and reported in millimeters.

It should be noted that the comparison was conducted using the significant result maps rather than the unthresholded maps. This approach allowed us to assess the similarity of the results with those that would typically be reported in a meta-analytic study.

#### 2.2.2 Comparison of MC to a contrast derived from a large-sample single study

We also compared MC of 2-back > 0-back to the results of a contrast derived from a highly powered large single study dataset, calculating voxel-wise, cluster-wise, and peak-wise comparisons in the same way as for comparisons with CM. For this, we used task-based fMRI data from the working memory task of an unrelated sample of the Human Connectome Project (HCP) (Van Essen et al., 2013). Details regarding the dataset, sample, and preprocessing as well as the results can be found in theSupplementary Material. The maps of the two “comparison maps,” that is, CM and HCP demonstrated a voxel-wise Jaccard index of 0.27.

## 3 Results

### 3.1 Description of the datasets

To investigate the effects captured in meta-analytic contrasts (MCs) in comparison with meta-analyses across the same contrasts at the experimental level (CMs), we compared four MC analyses and corresponding CM analyses across three different cognitive domains. The final datasets included 134 experiments for working memory: 2-back > 0-back, 118 experiments for working memory: 2-back > 1-back, 124 experiments for interference processing, and 146 experiments for emotional face processing. Most studies reported experiments for only one condition of interest (A, B or A>B), but there were also those that provided experiments for two or all three contrasts of interest. All three contrasts of interest (i.e., A, B, A>B) were available for 12 studies in WM: 2-back > 0-back, for 6 studies in WM: 2-back > 1-back, for 15 studies in interference processing, and for only 3 studies in emo dataset. Both sets of coordinates required to compute the MC (i.e., A, B) were available for 16 studies in WM: 2-back > 0-back, 17 studies in WM: 2-back > 1-back, 20 studies in interference, and 17 studies in emo datasets.

### 3.2 Comparison between CM and MC

Overall, voxel-wise comparisons revealed disparities in the number of significant voxels between CM and MC, with CM exhibiting a larger network for all datasets (seeFigures 3–6). Thus, CM is, in general, more sensitive than MC. For the working memory (2-back > 0-back) and interference processing contrasts, the voxels identified by MC largely aligned with the CM network. However, this correspondence was not observed for the 2-back > 1-back and emotional face processing contrasts. At the cluster level, several of the MC clusters overlapped with those of CM.

**Fig. 3.**
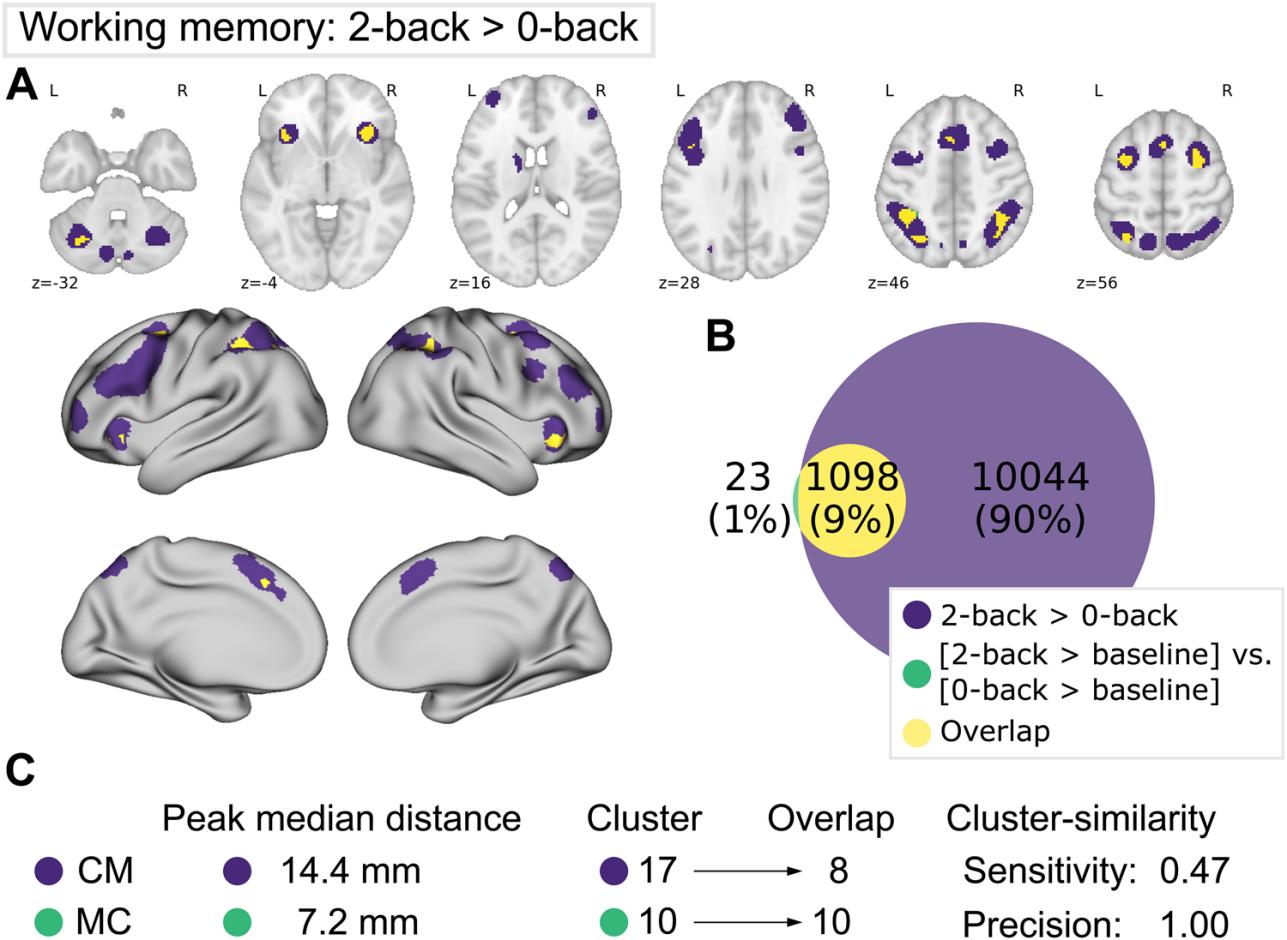
Comparison of meta-analytic contrast (MC, green) and contrast-meta (CM, purple) for 2-back versus 0-back. (A) MC and CM projections on MNI152 volume and fsLR (FreeSurfer surface template) surface. (B) Venn diagram depicting the absolute voxel-wise overlap between the two maps (yellow). (C) Median distance between CM peaks and their nearest MC peaks, and vice versa. Cluster-wise overlap by count of CM and MC clusters overlapping with any cluster of the opposite map. Sensitivity shows the proportion of overlapping CM clusters with MC clusters, precision as overlapping MC clusters with CM.

**Fig. 4.**
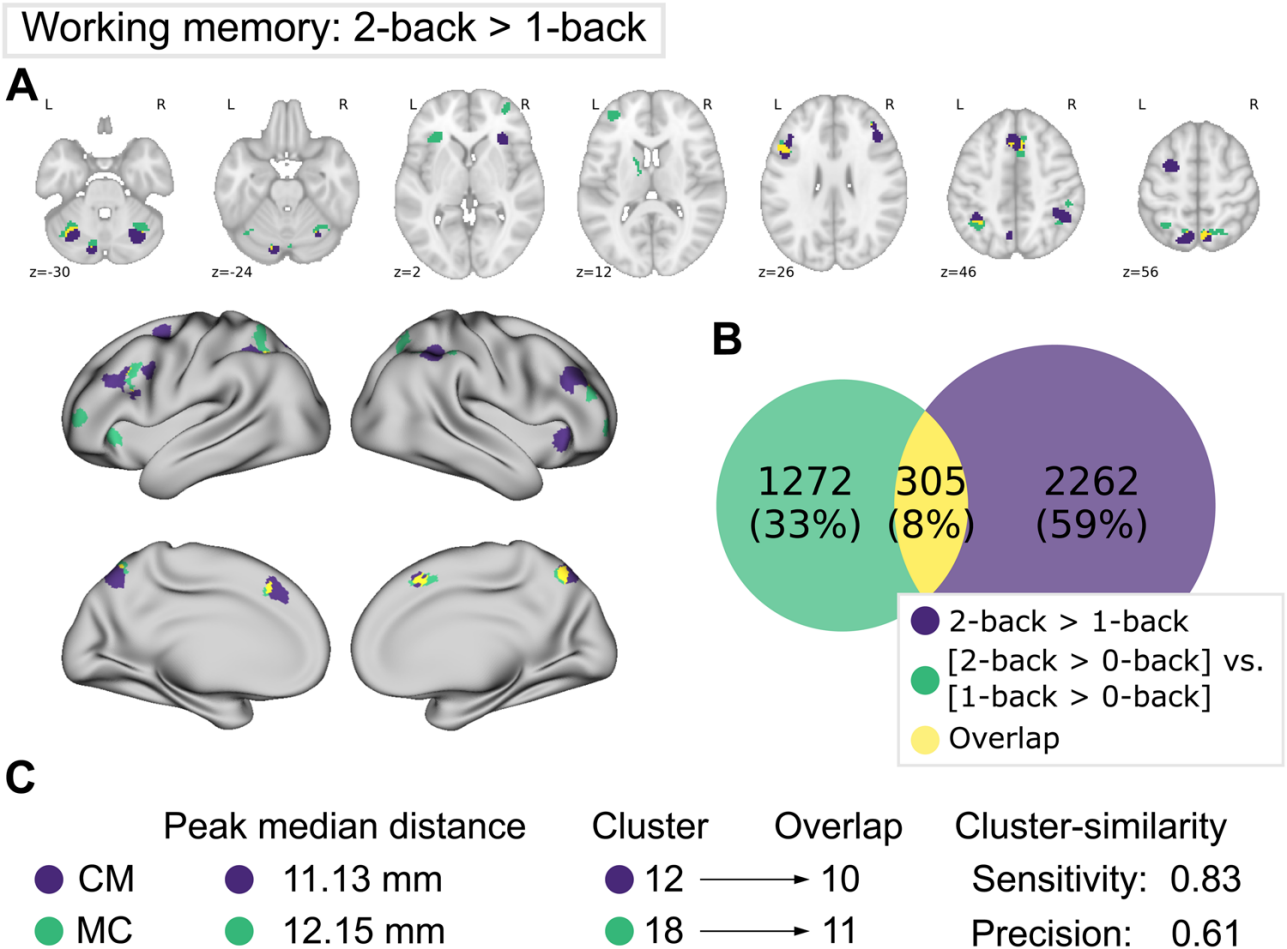
Comparison of meta-analytic contrast (MC, green) and contrast-meta (CM, purple) for the working memory 2-back versus 1-back dataset. (A) MC and CM projections on MNI152 volume and fsLR surface. (B) Venn diagram depicting voxel-wise overlap between CM and MC (yellow). (C) Proximities of peaks and cluster-wise overlap between the maps. Refer to previous caption for detailed descriptions.

**Fig. 5.**
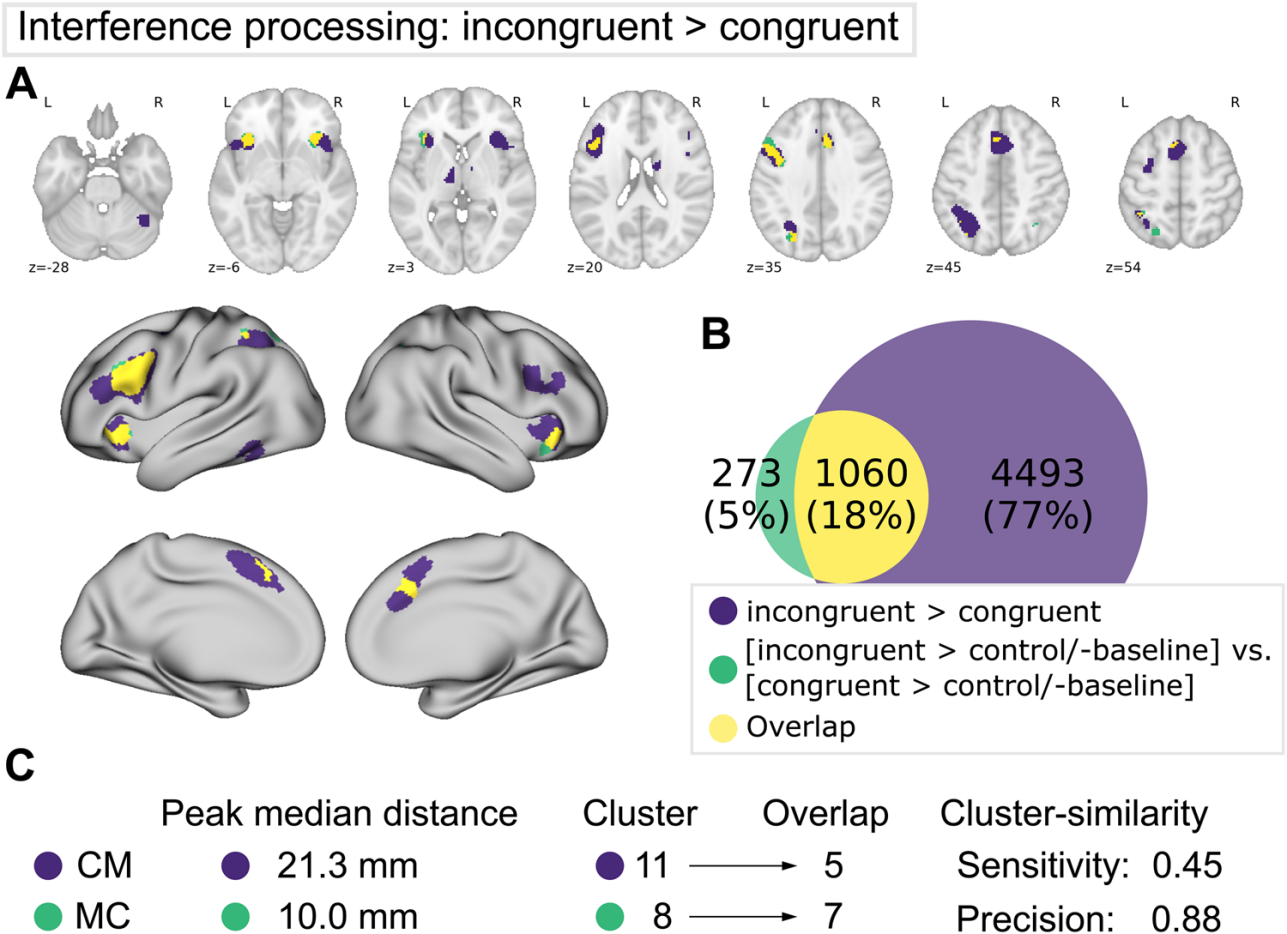
Comparison of meta-analytic contrast (MC, green) and contrast-meta (CM, purple) for the interference dataset. (A) MC and CM projections on MNI152 volume and fsLR surface. (B) Venn diagram depicting voxel-wise overlap between CM and MC (yellow). (C) Proximities of peaks and cluster-wise overlap between the maps. Refer to previous caption for detailed descriptions.

**Fig. 6.**
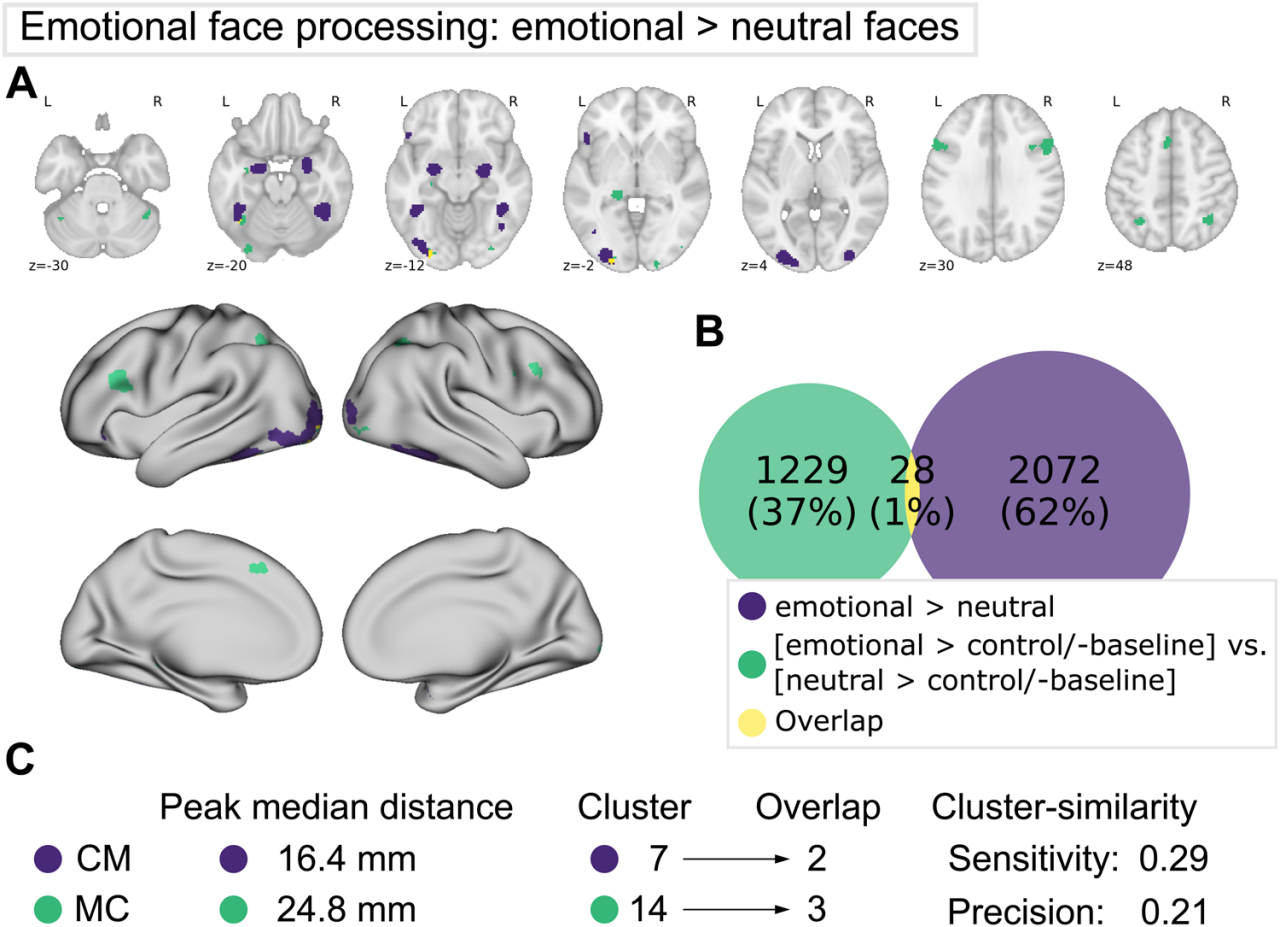
Comparison of meta-analytic contrast (MC, green) and contrast-meta (CM, purple) for the emotional face processing dataset. (A) MC and CM projections on MNI152 volume and fsLR surface. (B) Venn diagram depicting voxel-wise overlap between CM and MC (yellow). (C) Proximities of peaks and cluster-wise overlap between the maps. Refer to previous caption for detailed descriptions.

#### 3.2.1 Working memory

##### 3.2.1.1 2-back > 0-back

As shown inFigure 3, the classic CM yielded a more extensive network in terms of regions and significant voxels, as compared to the MC results. Voxel-wise Jaccard coefficients and sensitivity are thus rather low (~0.1, seeSupplementary Table S1). However, all regions showing significantly stronger convergence in the 2-back > baseline meta-analysis, as compared to the 0-back > baseline meta-analysis (MC), were located entirely within the CM network, reflected by high precision (0.98) and perfect cluster and peak overlaps (Fig. 3). Regions delineated in both types of contrast analyses included bilateral intraparietal sulcus (IPS), dorsal premotor cortex (dPMC), (pre-) supplementary motor area (SMA), anterior insula, and left cerebellum; in contrast, 3 out of 4 cerebellar regions, left basal ganglia, bilateral dorsolateral prefrontal cortex (dlPFC), frontal pole, as well as inferior frontal junction (IFJ) were not identified in MC, which is reflected in low cluster-wise sensitivity (0.47) and relatively far median peak distance (14.4 mm).

##### 3.2.1.2 2-back > 1-back

In a second dataset based on the WM domain, we focused on conditions with different working memory loads (i.e., 2-back > 1-back). At the voxel level, both networks seem to differ substantially (compareFig. 4andSupplementary Table S1). However, many clusters of MC and CM were located directly next to each other and therefore the comparison by cluster and peak locations revealed relatively good correspondence of MC and CM (compareFig. 4). Regions for which significant differences were found in both contrast analyses included the bilateral IPS, dlPFC, (pre-)SMA, and 3 clusters in the cerebellum. Left dorsal premotor cortex and right anterior insula were only identified in CM, while bilateral frontal pole clusters and left insula were only found for MC.

#### 3.2.2 Interference processing

The comparison of the interference meta-analytic contrast (MC) with the results of the contrast-meta (CM) yielded a similar pattern as seen for the WM 2-back > 0-back comparison (seeFig. 5). However, for the interference contrast, higher voxel-wise similarity was found together with lower precision (Supplementary Table S1). The results of the peak comparison showed a moderate correspondence from MC to CM (with a median distance of 10 mm), while this was relatively lower for CM to MC (with a median distance of 21.3 mm). Similarly, the cluster-wise comparison showed that almost all regions revealed by MC have overlapping clusters in CM, while many clusters revealed by CM had no correspondence in MC (compareFig. 5). Regions found in both contrast approaches comprised bilateral anterior insula, (pre-)SMA, midcingulate cortex, left IPS, and left dlPFC. For CM bilateral thalamus, right cerebellum and left fusiform gyrus were additionally identified, while MC revealed a cluster in right IPS.

#### 3.2.3 Emotional face processing

For emotional face processing, CM and MC revealed networks with little to no similarity (seeFig. 6). The only regional correspondence was observed in the left lateral occipital cortex, fusiform gyrus, and left amygdala. In contrast to the other domains (WM: 2-back > 0-back and interference), we observed twice as many clusters and peaks for MC than for CM (seeFig. 6). In both analyses, bilateral lateral occipital gyrus, left amygdale, and left fusiform gyrus were found (in slightly different locations). For CM right fusiform gyrus and left lateral orbitofrontal cortex were additionally found, whereas MC identified additional clusters in bilateral dlPFC and IPS, left hippocampus, right cerebellum, bilateral occipital pole, as well as pre-SMA.

## 4 Discussion

Meta-analytic studies often test for differences between various mental faculties, groups, and other experimental factors that are not sufficiently enough contrasted in the neuroimaging literature (Fehlbaum et al., 2022;Klugah-Brown et al., 2021;Swick et al., 2011) by computing separate meta-analyses for each condition of interest. However, the results of individual meta-analyses do not provide any information about where in the brain convergence differs between these analyses. For instance, the presence of convergence in a specific brain region found in one meta-analysis and absence of the same region in another one does not necessarily imply that one of them shows less consistency across experiments than the other. Meta-analytic contrast analyses are, thus, important tools to formally test for these differences and should be provided in any meta-analytic study that claims to interpret differences between individual analyses results. However, while the statistical relevance and usefulness of meta-analytic contrast is out of question, it is not fully evaluated which exact conclusions can be drawn from the results. This empirical investigation aimed to assess the extent to which meta-analytic contrasts (MC), as implemented in the ALE meta-analysis framework, reflect the effects observed in standard meta-analyses across experiment-level contrasts (CM). Results revealed that meta-analytic contrasts revealed, in general, less differences between conditions than CM together with a high rate of precision for most datasets, except emotion processing, the dataset where tasks, control conditions, and stimuli varied most. Therefore, for most datasets, regions found in meta-analytic contrast analysis can quite confidently be interpreted in a similar way as results of classic CMs, that is, consistent activation differences between conditions. However, an absence of differences in meta-analytic contrast results does not necessarily imply the absence of consistent activation differences. This is especially true for regions that overlap across the two meta-analyses that are compared to each other, as well as to low-powered and/or datasets with a lot of experimental and methodological variation. Thus, meta-analytic contrasts, in addition to providing statistical evidence of differences in convergence between the results of separate analyses, are well suited for complementary and exploratory meta-analytic investigations, especially in situations where there is insufficient literature reporting the exact contrast of interest. However, the results of MC should always be interpreted considering the specific characteristics of the datasets and potential systematic confounds be assessed that could affect the results.

### 4.1 Functional relevance of identified regions

When looking at the specific regions revealed for the working memory and interference processing domains, CM and MC both reveal networks including, frontal and parietal regions as well as the anterior insula and (pre)SMA. These regions are part of the multiple demand (Camilleri et al., 2018;Duncan, 2010), central executive (Menon, 2011), or cognitive control (Cole & Schneider, 2007) networks and are thus scientifically meaningful to be involved in working memory and interference processing. Interestingly, most regions that are additionally found in CM or MC, like the more anterior dlPFC, dPMC, and subcortical regions (basal ganglia and thalamus) are part of the so-called extended multiple demand network (Camilleri et al., 2018), regions involved in executive functions but, in contrast to the core multiple demand regions, more dependent on specific cognitive demands. These regions might, therefore, show smaller and less robust effects and are potentially only found when contrasted against a specific high-level control condition or in experiments with specific task characteristics.

For the emotion processing domain, in turn, there were only few regions that were found in both contrast approaches. While CM primarily identified classical regions of (facial) emotion processing, that is, bilateral amygdala, fusiform, and inferior occipital gyrus as well as the left lateral orbitofrontal cortex (Adolphs, 2002;Dolcos et al., 2011), the contrast at the meta-analytic level (MC) revealed many regions (like dlPFC, IPS, preSMA, hippocampus) that are more implicated in emotional control as well as increased task demand (Dolcos et al., 2011;Duncan, 2010;Phillips et al., 2003). Thus, MC seems to pick up cognitive processes during emotion perception to a stronger degree than CM.

### 4.2 Regions showing convergence of differences but no differences in convergence

The results of this study revealed relatively low voxel-level similarity between MC and CM. This can be attributed to the generally lower number of significant voxels in MC. When looking at the results from a regional perspective, 45%–83% of the CM result clusters were also obtained in MC for WM and interference processing. However, still about half of the regions remained undetected and much less in the dataset of emotional processing. This is consistent with the results ofCosta et al. (2021), reporting less differences between groups when using MC, relative to contrast meta-analyses.

#### 4.2.1 Power

The fact that less voxels/regions show convergence in CM but not in MC might be due to low power in the individual meta-analyses, with more experiments needed for MC to detect the same effects as CM. In general, all the individual meta-analyses include enough experiments, that is, n > 21 experiments, for detecting strong effects and most of them are decently powered for detecting medium-size effects (Eickhoff et al., 2016). However, more power might be needed for finding differences in convergence (via MCs). This is supported by the results of the 2-back > 1-back comparison, exhibiting the highest cluster-level sensitivity and lowest median peak distance (CM to MC peaks) with 73 experiments included in the meta-analysis of 2-back > 0-back. When reducing the number of experiments to 22 experiments in the deterministically matched analyses (seeSupplementary Fig. S1), sensitivity decreased. However, this goes along with a lower level of precision and cannot be generalized to all the datasets, therefore indicating that a lack of power alone is unlikely to explain the detection of less voxels/clusters of MC.

A second factor that potentially influences statistical power is the heterogeneity between experiments (i.e., variations in tasks, stimuli, or control conditions) but also populations (variations in gender and age distribution, recruitment for clinical studies). For effect-size meta-analysis, the power of an analysis is affected by the degree of heterogeneity across studies (Jiang et al., 2010;Kenny & Judd, 2019;McShane & Böckenholt, 2014). Neuroimaging meta-analyses might be similarly affected and with increasing heterogeneity, disproportionately more experiments would be needed to find significant differences in the MC.

#### 4.2.2 Differences in activation strength are lost in MC

It is important to note that in CM, the factor of interest is modeled as a within-subject factor on the single-experiment level, while for MC the difference between conditions is based on the already sparser representation of the two to-be-compared main effects as peak coordinates. MC analysis thus adds an additional layer on top of classic ALE analyses, resulting in further information reduction. Consequently, the results of meta-analytic contrasts only indirectly include differences in brain activation and no information about effect sizes (Müller, Cieslik, et al., 2018). This sparser representation might affect regions that are involved in both conditions. Indeed, our results suggest that MC is especially insensitive for clusters that are found in both individual meta-analyses that are compared to each other. For example, the MC of emotional face processing where the individual meta-analyses overlap most (33% of the voxels of the individual meta-analysis emotion > baseline overlap with the voxels of neutral > baseline) reveals only few regions that are also found in CM. In turn, the MC with the highest amount of voxels that are also found in CM (interference processing) exhibited the lowest overlap of 13% (seeSupplementary Figs. S2–S5for the amount of overlap between the individual meta-analyses of MCs for all datasets). Thus, activation differences for regions involved in both conditions cannot be revealed by MC. However, stronger convergence in similar regions can be observed if regions are larger in extent in one of the individual analyses or are slightly shifted. Therefore, on the regional level, the problem of not detecting differences in convergence in overlapping regions can be mitigated by higher power of the individual meta-analyses, as the clusters become larger, the more experiments are included that show activation differences in a given area (Frahm et al., 2022). This is particularly evident in the WM 2-back > 1-back comparison. The extent of the clusters for 2-back > 0-back found also for 1-back > 0-back is much larger, and MCs can, therefore, on the regional level, reveal differences that are also apparent in CMs. The extent and location of convergence, thus, directly influence if a difference in convergence of a particular contrast can be detected.

Less differences in convergence compared to convergence in differences can additionally be attributed to effects driven by deactivations. For example, a region might be slightly activated in condition A and deactivated by condition B. Testing A against baseline as well as B against baseline in an individual fMRI experiment can thus lead to non-significant effects, but testing A against B would reveal significant effects. Thus, regions may be identified in CM but not in the meta-contrast. The converse also holds true, regions observed in individual experiments of A > baseline might be missing in A > B, not due to activations in the control condition B (cancelling out of effects) but due to slight activations in A and deactivations in the baseline condition, leading to significant activation differences in A > baseline. It should be noted that these theoretical considerations also apply to differences in activation in contrasts of single fMRI studies. Previous studies have shown that the activation strength and significance of regions can be directly influenced by the choice of initial conditions at the experiment level (Marx et al., 2004;Newman et al., 2001;Stark & Squire, 2001). This, therefore, has a direct impact on CM and MC results since activation strength is not considered in the MC calculation and sparsely represented regions will lead to non-significant results on a meta-analysis level. Thus, given that differences in activation are ignored in MC, both deactivations and regions activating on both conditions (overlapping regions) cannot be picked up by meta-analytic contrasts.

### 4.3 Regions showing differences in convergence but no convergence of differences

Cluster-wise precision and median peak distance are quite good for the working memory and interference processing datasets, but for emotional face processing there are a lot of regions found in MC but not CM. Thus, for the two datasets from the cognitive domain, there are only a few additional regions in MC, while for the emotional domain the results look different. This can be attributed to systematic experimental and methodological differences between experiments included in the two meta-analyses of MC which are not related to the condition of interest as well as sub-optimal cognitive subtraction and thresholding of the original studies. For example, task and stimulus effects that were present in the “emotional > baseline” meta-analysis might have been absent or weaker in the “neutral > baseline” meta-analysis, resulting in task-specific effects not being subtracted in MC. Similarly, general task demands that are unrelated to the process of interest might only become apparent when contrasting against baseline conditions. Thus, divergent effects between MC and CM might be the results of suboptimal cognitive subtraction, especially for baseline contrasts. This becomes especially apparent for the emotion dataset, where primarily regions involved in increased task demands are identified in MC, rather than regions involved in emotional face processing.

The emotional face processing dataset shows a lot of variation of the face and control stimuli, as well as in the task performed. Experiments testing the contrast between emotional and neutral faces (which are included in CM) typically use only face stimuli and do not vary the task (e.g.,Seara-Cardoso et al., 2016;Williams et al., 2001). In contrast, in experiments testing emotional or neutral against control/baseline (which are included in MC), both face and non-face stimuli, and the task is varied for face and control stimuli (i.e., emotion matching for faces and shape matching for control stimuli, e.g.,Binelli et al., 2016;Via et al., 2014). Together with very similar regions in face-processing-related regions, this could have led to a meta-contrast of more emotion-unspecific and more task-related effects (seeSupplementary Fig. S5). For example, parietal regions, which are found in MC for emotion processing, play a major role in attentional shifts (Langner et al., 2011;Serences et al., 2004;Shomstein & Yantis, 2004;Townsend et al., 2006). Therefore, it is quite likely that stronger convergence for the meta-analytic contrast between emotional versus neutral faces reflects those attentional shifts between facial and control stimuli and not necessarily emotional processes. This is supported by the results of the matched analyses where parietal clusters are no longer found for MC when the control condition is matched.

In summary, systematic differences and suboptimal cognitive subtraction in experiments used for MC computation may introduce confounds. These are not unique to task-focused meta-analyses but may be even more prevalent in MCs between groups (such as young vs. old, patients vs. control, disease A vs. disease B), since there may be confounding factors in the compared groups that are difficult to control, in addition to the systematic variations in task, control condition, and stimuli.

Therefore, our results highlight the susceptibility of MC for effects of systematic confounds between the contrasted datasets as well as imperfect cognitive subtraction. These should be considered in the planning, analyses, interpretation, and reporting of MC results by applying appropriate inclusion criteria, providing a detailed characterization of the experiments of the datasets, evaluation, and accounting of variables of no interest that may systematically differ between the two meta-analyses as well as transparent reporting.

### 4.4 Limitations and outlook

Some of the analyses presented here were only made possible thanks to additional non-peer-reviewed results sent to us by authors. While this approach introduces a potential new source of variance, it is not uncommon in the meta-analytic literature (Müller, Cieslik, et al., 2018). Furthermore, we were only able to gather all three contrasts of interest (experiments) for a limited number of studies, so we could not eliminate the effects generated by the heterogeneity of experiments. This may have been particularly evident in the emotional face processing comparison. Finally, we restricted ourselves to task fMRI contrasts between different cognitive conditions. Group contrasts, such as patients versus controls (Costa et al., 2021;Degasperi et al., 2021), were not investigated here. Therefore, caution should be exercised when generalizing the results discussed here to such scenarios, which contain even more sources of heterogeneity.

It is important to acknowledge that the individual meta-analyses between which MC was calculated differed in terms of sample size, with condition A (vs. baseline) always included more experiments than condition B (vs. baseline). This might have influenced the results, given a difference in power between the two analyses. One potential solution would be the use of balanced contrasts (Tamon et al., 2024). However, for this evaluation, we chose to calculate MC in the traditional way, as the results are better generalizable to most applications in the literature.

Importantly, although we compared MC to the results of CM and calculated metrics such as sensitivity, we do not claim that CM is the gold standard and free of any bias or problems. That is, systematic confounds that are common across experiments of CM can also lead to convergence in CM that is not necessarily related to the process of interest. Furthermore, as discussed above, coordinate-based meta-analyses use a sparse representation of results of individual studies, with coordinates being strongly dependent on the methodological and reporting characteristics of the individual studies (thresholding, number of peaks reported, etc.). Thus, to allow generalizations and conclusions that go beyond the effects found for CM, MC should ideally be compared to results of the contrast of interest derived from different approaches. This could, for example, be results based on a large-sample single study (as already done for 2-back > 0-back here; reported in theSupplementary Material) or of an image-based meta-analysis (Salimi-Khorshidi et al., 2009). Unfortunately, the latter is often hampered by a lack of availability of unthresholded maps of individual studies.

Lastly, beyond the contrast-analyses used and implemented for ALE meta-analyses, differences between conditions can also be tested by modelling categorical regressors in meta-regression analyses. While this is not currently implemented for ALE, Bayesian spatial regression models (Samartsidis et al., 2019) and coordinate-based meta-regression models (Yu et al., 2024) would allow the modeling of covariates, reflecting a different approach to group comparisons. AlthoughSamartsidis et al. (2019)reported similar results from meta-regression and the contrast analyses of ALE, a comparison of MC to the results of meta-regression would be a valuable extension of the current project.

## 5 Conclusion

ALE meta-analytic contrasts are a widely used method for comparing results between individual meta-analyses and are a necessary extension for meta-analytic studies that claim differences in regional convergence between two analyses across experiments of different conditions, states, or groups. Our results show that these contrasts in most cases capture similar results to standard meta-analyses, but results can suffer from low power, systematic confounds, and a sparser representation of effects. Thus, meta-analytic contrasts are beyond demonstrating significant differences in convergence between meta-analytic results, well suited for complementary and exploratory meta-analytic investigations. However, they should not be understood as a direct substitute for classic meta-analyses across experiment-level contrasts reported in the literature, and the regions found should be interpreted accordingly.

## Supplementary Material

Supplementary Material

## Data Availability

All ALE meta-analyses were computed using pyALE, accessible athttps://github.com/LenFrahm/pyALE. Code for comparing the meta-analytic results and plotting figures can be found athttps://github.com/vinkue/ale-meta-contrasts. The data are available upon request, as the coordinates include data from other research groups that have not been consented for redistribution. The code used to compute the large sample single study contrast was based on the HCPpipelines (https://github.com/Washington-University/HCPpipelines) and the Python exemplary analysis byEsteban et al. (2020)(https://github.com/poldracklab/ds003-post-fMRIPrep-analysis). Data from the Human Connectome Project Young Adults are available athttps://db.humanconnectome.org/.
