# Supplementary material for: Consistent activation differences versus differences in consistent activation: Evaluating meta-analytic contrasts
Source: Imaging Neurosci (Camb). 2024 Nov 8;2:imag-2-00358. doi: 10.1162/imag_a_00358 (PMC12315735; doi:10.1162/imag_a_00358)
Supplement: Supplementary Material [file imag_a_00358-supp.pdf]

Supplement - Consistent activation differences versus differences in consistent activation: Evaluating meta-analytic contrasts

Vincent Küppers[1,2,✉], Edna C. Cieslik[3,1], Lennart Frahm[4,1], Felix Hoffstaedter[1,3], Simon B. Eickhoff[3,1], Robert Langner[3,1], and Veronika I. Müller[1,3,✉]

[1] Institute of Neuroscience and Medicine, Brain and Behaviour (INM-7), Research Center Jülich, Jülich, Germany

[2] Department of Nuclear Medicine, University Hospital and Medical Faculty, University of Cologne, Cologne, Germany

[3] Institute of Systems Neuroscience, Medical Faculty and University Hospital Düsseldorf, Heinrich Heine University Düsseldorf, Düsseldorf, Germany

[4] Department of Psychiatry, Psychotherapy and Psychosomatics, School of Medicine, RWTH Aachen University, Aachen

✉ Correspondence: Vincent Küppers <v.kueppers@fz-juelich.de>, Veronika I. Müller <v.mueller@fz-juelich.de>

1 Supplement: Methods

1.1 Datasets

Working memory (WM): 2-back > 0-back. The initial WM: 2-back > 0-back dataset was based on n-back papers collected by Rottschy et al. (2012) and Langner & Eickhoff (2013). This dataset was extended by reference tracing of other previously published working-memory meta-analyses (Mencarelli et al., 2019; Yaple et al., 2019; Zhang et al., 2021). The following n-back specific keywords were used in the literature search: “n-back” OR “2-back” OR “two-back” OR OR “0-back” OR “zero-back,” limiting the search to abstracts and title. Here we included all experiments using the n-back paradigm in

different variation (e.g., verbal, spatial tasks; visual or auditory stimuli) and reporting one or more of the following comparisons: 2-back > 0-back, 2-back > baseline, 0-back > baseline. Baseline, comprised all conditions with a passive, low-level paradigm e.g., rest, passive perception of stimuli, fixation baseline. The resulting dataset consisted of 134 experiments from 104 individual studies

Working memory (WM): 2-back > 1-back. The dataset was again constructed using papers collected by Rottschy et al. (2012) and Langner & Eickhoff (2013) and extended by reference tracing of other previously published working-memory meta-analyses (Mencarelli et al., 2019; Yaple et al., 2019; Zhang et al., 2021). The following n-back specific keywords were used in the literature search: “n-back” OR “2-back” OR “two-back” OR “1-back” OR “one-back,” limiting the search to abstracts and title. n-back paradigms in different variations (e.g., verbal, spatial tasks; visual or auditory stimuli) and reporting one or more of the following comparisons: 2-back > 0-back, 2-back > 1-back, 1-back > 0-back were included. The resulting dataset consisted of 118 experiments from 91 individual studies.

Interference processing (interference). The initial interference dataset was based on experiments assessing the verbal colour-word Stroop task collected by Cieslik et al. (2015). Further, literature was searched by using the keyword “Stroop” with neuroimaging keywords. We included only colour word variants of the Stroop task (i.e., excluding auditory, gender, spatial etc. versions). We included results of the comparisons between incongruent > congruent; incongruent > control; incongruent > baseline; congruent > control; congruent > baseline. The control condition was defined as the presentation of neutral words, letters or symbols and the baseline condition as a passive fixation/ rest stimuli. The resulting dataset consisted of 124 experiments from 83 individual studies.

Emotional face processing (emo). The initial emo dataset was based on papers collected by Müller et al. (2018). This dataset was extended by reference tracing of the meta-analyses by Schurz et al. (2021) and Liu et al. (2021) and literature search using the following dataset specific keywords: “face” AND (“neutral” OR “emotional”). We

included different visual paradigms (e.g., emotional attention, passive viewing, gender discrimination tasks) presenting static pictures of human faces without any additional auditory input. Results of the following conditions of interest were included: emotional > neutral, emotional > control, emotional > baseline, neutral > control, neutral > baseline. Experiments using morphed faces (i.e., morphing faces between neutral and emotional expressions) were excluded to keep emotional and neutral stimuli as separate as possible. Similarly, paradigms using masking were excluded. We included experiments with different control tasks (e.g., contrasting to scrambled faces/pictures, objects, houses, geometrical shapes) or passive low level baselines (e.g., fixation). The resulting dataset consisted of 146 experiments from 122 individual studies.

1.2 Large sample single study contrast

Sample. We used the minimally preprocessed task-based fMRI data from the working memory task from 435 unrelated participants (240 females, age mean = 28.8, age STD = 3.69) from the Human Connectome Project Young Adults S1200 release (Glasser et al., 2013; Van Essen et al., 2013). All participants were without known quality control issues and scored with > 50% accuracy in the 2-back condition of the working memory (WM) n-back task.

Participant-level contrast. Volume based minimally preprocessed time-series for each run were analyzed using modified scripts from the HCPpipeline ((Barch et al., 2013), https://github.com/Washington-University/HCPpipelines), which are based on the FSL FEAT module (Woolrich et al., 2001, 2004). First, temporal high pass filtering (200 s cutoff) and spatial smoothing (8mm FWHM) were applied. GLM model fitting was performed by including eight blocked predictors, modelling each stimulus type (faces, places, body, tools) for each n-back condition (0-back, 2-back). The blocked predictors were convolved with a “canonical” hemodynamic response function (HRF). Regressors of no interest included 12 motion parameters from rigid-body transformation and their temporal derivatives to account for motion artifacts and derivates of each predictor to account for slice timing variance and HRF delay (Barch et al., 2013). To combine the

two runs, a fixed-effects analysis was computed within participants across the results of the GLM for both runs to compute a participant-level linear contrast for 2-back vs. 0-back.

Group-level contrast. Group-level GLM was calculated based on modified scripts of a Python NiPype-based workflow (Esteban et al., 2020). The FSL FLAME module was used to compute a one-sample t-test across the 435 participants. Effect size maps (Cohen's d) were computed by dividing the group-level contrasts of parameter estimate (COPE) by the square root of the estimated variance of parameter estimate (VARCOPE) divided by the square root of the sample size (Poldrack et al., 2017). These effect size maps were then thresholded at $d = 0.5$. A medium effect size according to Cohen (Cohen, 2013) was chosen as ALE meta-analyses primarily detect moderate to strong effects (Eickhoff et al., 2016; Salimi-Khorshidi et al., 2009).

1.3 Coding of sent results / neurovault maps

Some authors kindly provided us with additional results as either coordinate tables or statistical maps. Furthermore, for some publications we could download result maps from neurovault (Gorgolewski et al., 2016). Unthresholded statistical maps were first thresholded in the same manner as was done in the respective paper. In the rare cases that no information was provided in the respective paper, a threshold of $p > 0.001$ uncorrected was chosen and an additional cluster extent threshold of $k = 5$. From the thresholded maps, peaks with a minimum distance of 8 mm were extracted with the FSL (version 6.0.3) tool cluster. Only the first 20 peaks per cluster were considered. In case authors sent a table of coordinates, we included the peak coordinates as provided by the authors, but maximum 20 peaks per cluster.

1.4 Experiment matching

The number of experiments varies between the different meta-analyses of the meta-analytic contrast (MC), such as 38 experiments for 2 > baseline and 23 experiments for 0 > baseline, in the working memory domain. Additionally, there were variations in the

type of stimuli, task, and the number of participants included in the experiments, particularly within the more heterogeneous datasets.

To ensure comparability between the meta-analyses across contrasts and the meta-analytic contrast, deterministic matching was performed for each domain, including the working memory domain with both 2-back > 0-back and 2-back > 1-back analyses. Initially, studies were included that reported all three sets of coordinates (i.e., *condition A > condition B*, *condition A > baseline*, and *condition B > baseline*), or at least two sets of coordinates.

Next, experiments were manually selected so that for each contrast of interest there were a similar number of experiments with comparable characteristics. The matching process considered factors, such as the number of participants, the type of stimuli, the contrasting condition (low-level-baseline and control stimuli), and the tasks (in case of emotional face processing).

2 Supplement: Results

2.1 Main analysis

2.1.1 Voxel-wise comparison

Supplementary Table S2.1: Voxel-wise similarity of meta-analytic contrast (MC) and contrast-meta (CM) as well as MC of WM and large-sample group map (HCP).

	Comparison	Jaccard	Sensitivity	Precision
WM: 2-back > 0-back	MC vs. CM	0.098	0.099	0.979
	MC vs. HCP	0.028	0.029	0.963
WM: 2-back > 1-back	MC vs. CM	0.079	0.119	0.193
Interference processing	MC vs. CM	0.182	0.191	0.795
Emotional face processing	MC vs. CM	0.008	0.013	0.022

2.2 Matched analysis

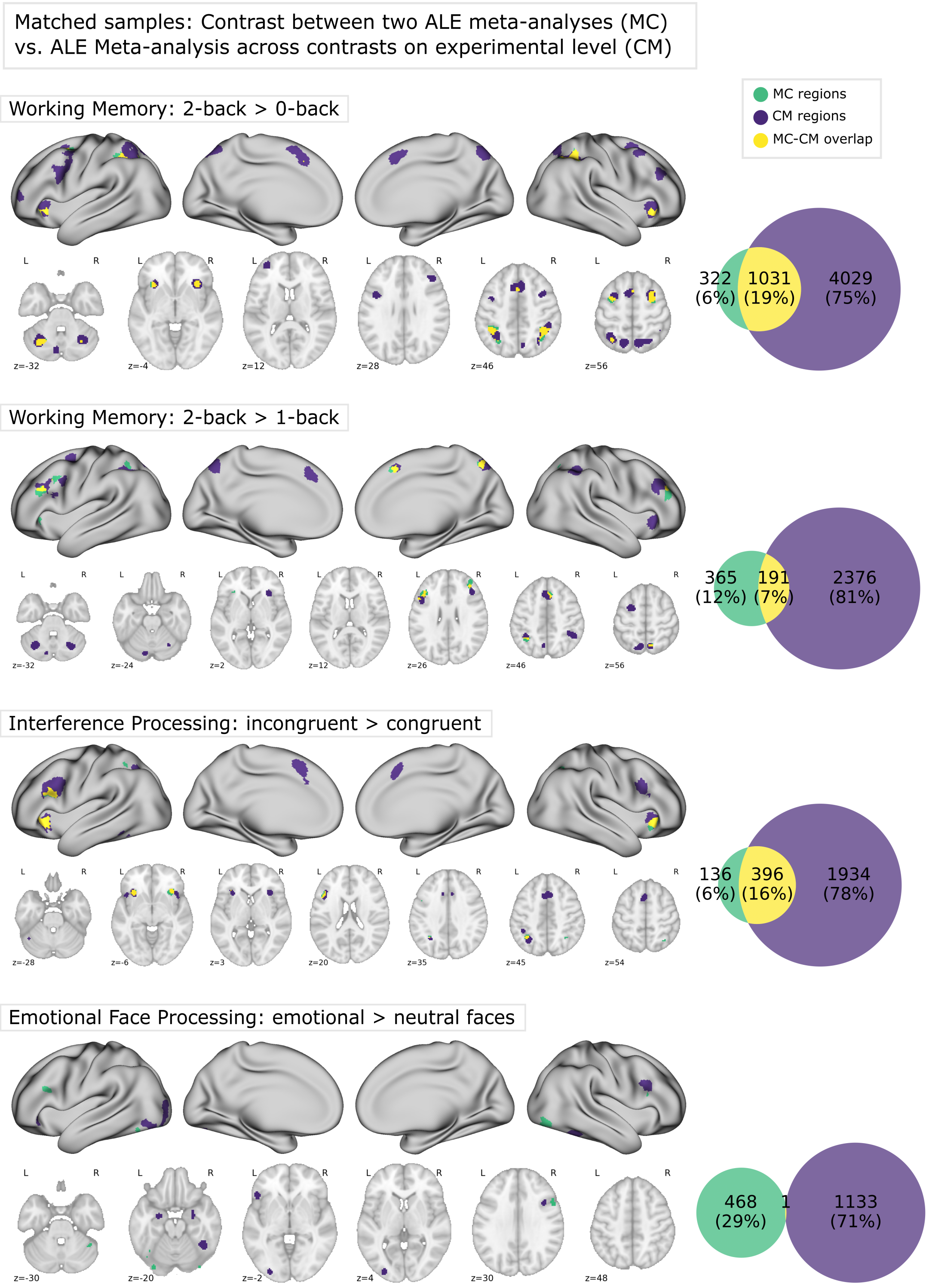

Supplementary Figure S2.1: Meta-analytic contrast between two meta-analyses (MC) vs. meta-analysis across contrasts (CM) of deterministically matched samples.

Supplementary Table S2.2: Voxel-wise similarity. Quantification of similarity on a voxel-wise level between matched meta-analytic maps.

	Comparison	Jaccard	Sensitivity	Precision
WM: 2-back > 0-back - matched	MC vs. CM	0.192	0.204	0.762
	MC vs. HCP	0.034	0.035	0.976
WM: 2-back > 1-back - matched	MC vs. CM	0.065	0.074	0.344
Interference Processing - matched	MC vs. CM	0.161	0.170	0.744
Emotional Face Processing - matched	MC vs. CM	0.001	0.001	0.002

Supplementary Table S2.3: Peak- and cluster-level comparisons of deterministically matched meta-analytic contrasts (MC), meta-analyses across contrasts (CM), and the group-level map for WM (HCP). Peak distances as median distance between CM peaks and their nearest MC peaks, and vice versa.

	Comparison	Peaks MC to CM [mm]	Peaks CM to MC [mm]	Cluster Sensitivity	Cluster Precision
WM: 2-back > 0-back - matched	MC vs. CM	6.0	8.9	0.0	0.00
	MC vs. HCP	8.2	29.4	0.3	1.00
WM: 2-back > 1-back - matched	MC vs. CM	8.2	12.1	0.2	1.00
Interference Processing - matched	MC vs. CM	6.7	37.6	0.2	1.00
Emotional Face Processing - matched	MC vs. CM	25.5	15.5	0.2	0.71

2.3 MC: individual meta-analyses

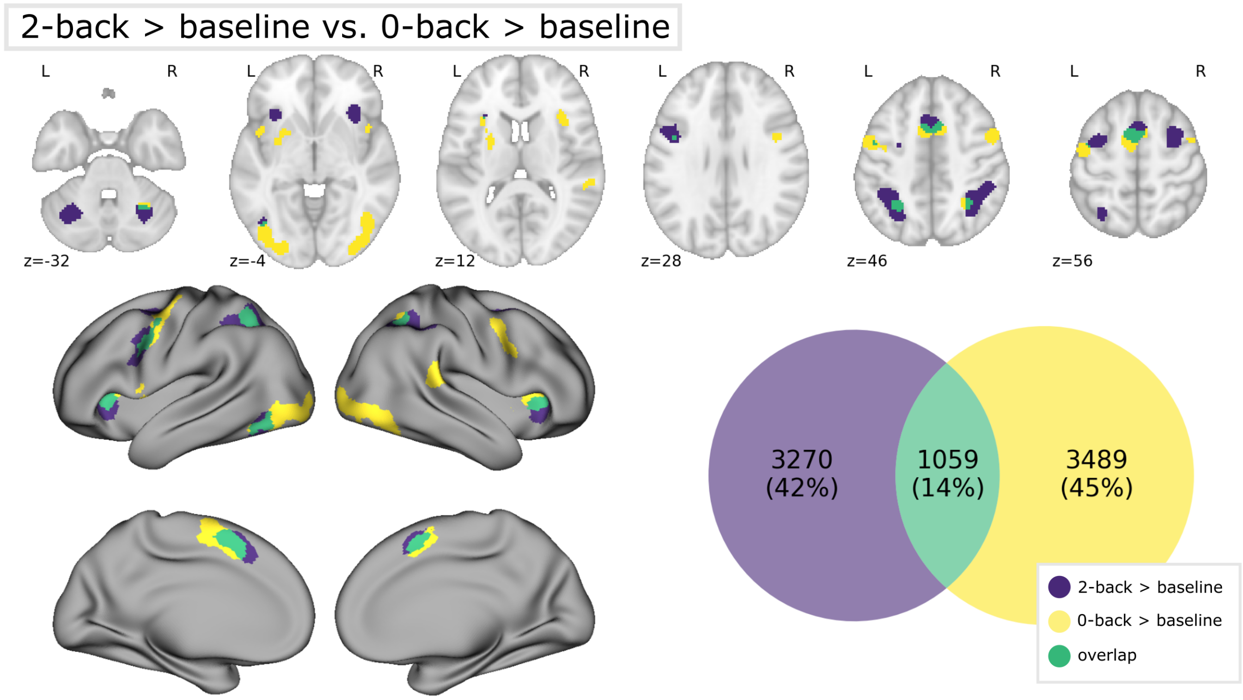

Supplementary Figure S2.2: 2-back > baseline (38 experiments) vs. 0-back > baseline (20 experiments). Individual meta-analysis (cFWE 0.05 corrected) used for computation of meta-analytic contrast between two meta-analyses (MC) overlayed on each other.

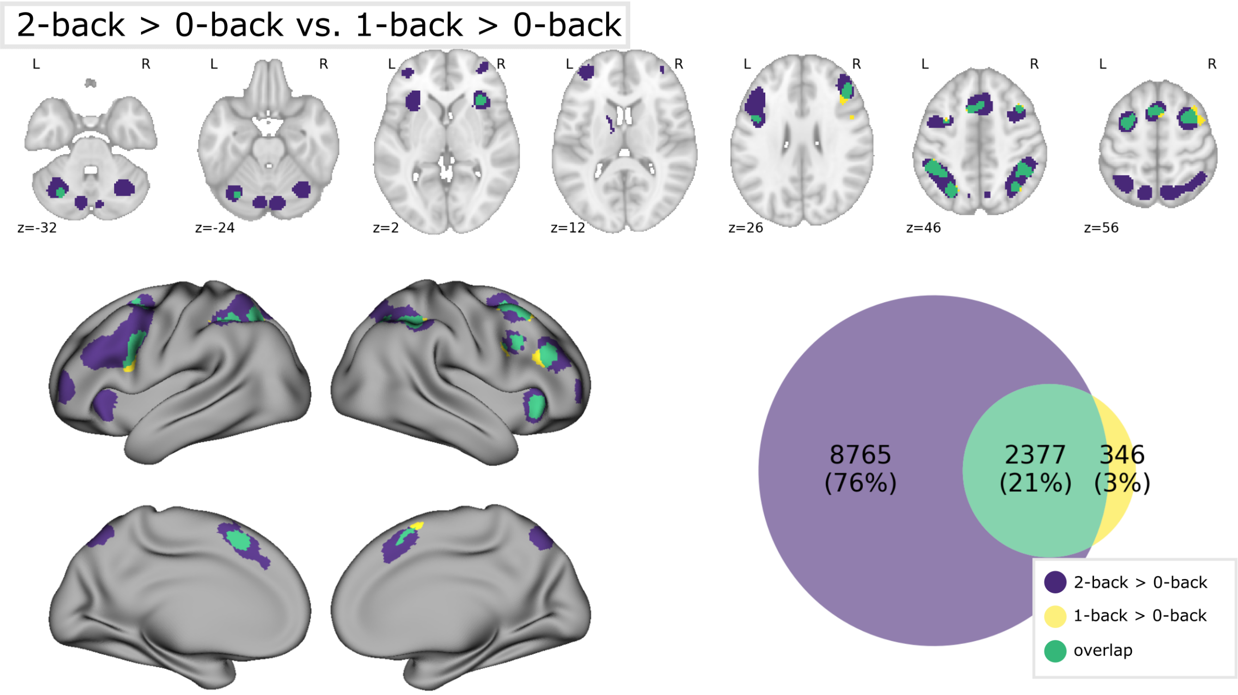

Supplementary Figure S2.3: 2-back > 0-back (73 experiments) vs. 1-back > O-back (22 experiments). Individual meta-analysis (cFWE 0.05 corrected) used for computation of meta-analytic contrast between two meta-analyses (MC) overlayed on each other.

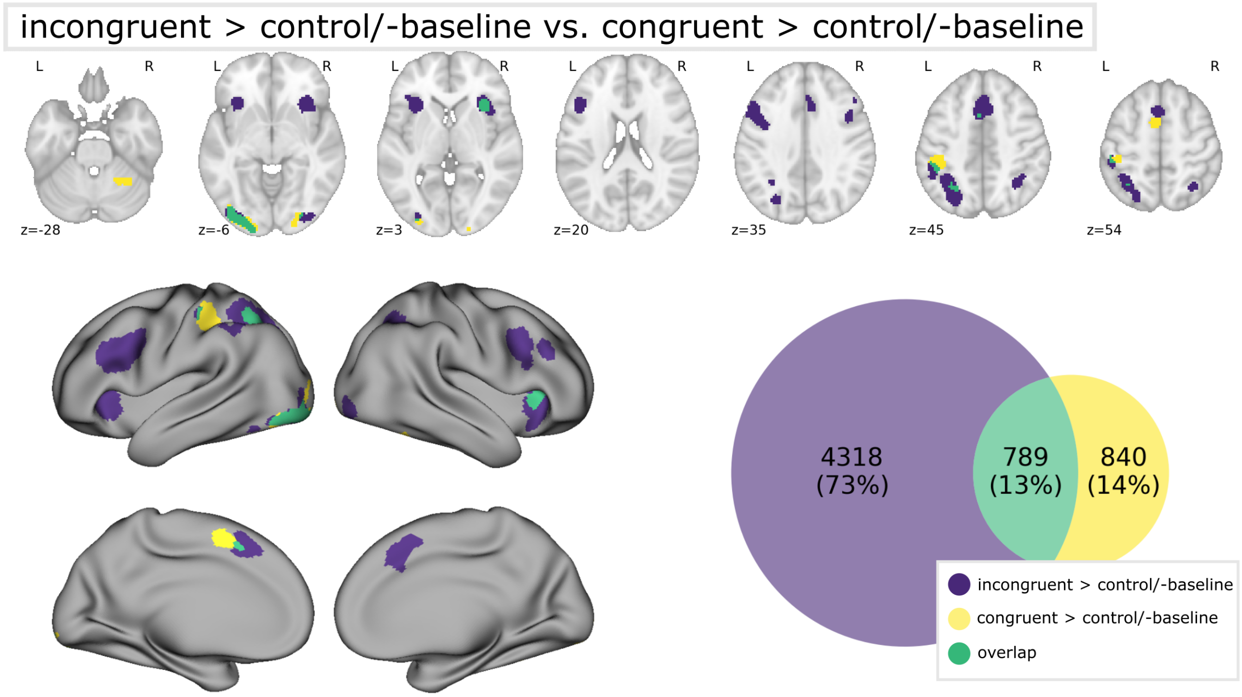

Supplementary Figure S2.4: Incongruent > control/-baseline (47 experiments) vs. congruent > control/-baseline (21 experiments). Individual meta-analysis (cFWE 0.05 corrected) used for computation of meta-analytic contrast between two meta-analyses (MC) overlayed on each other.

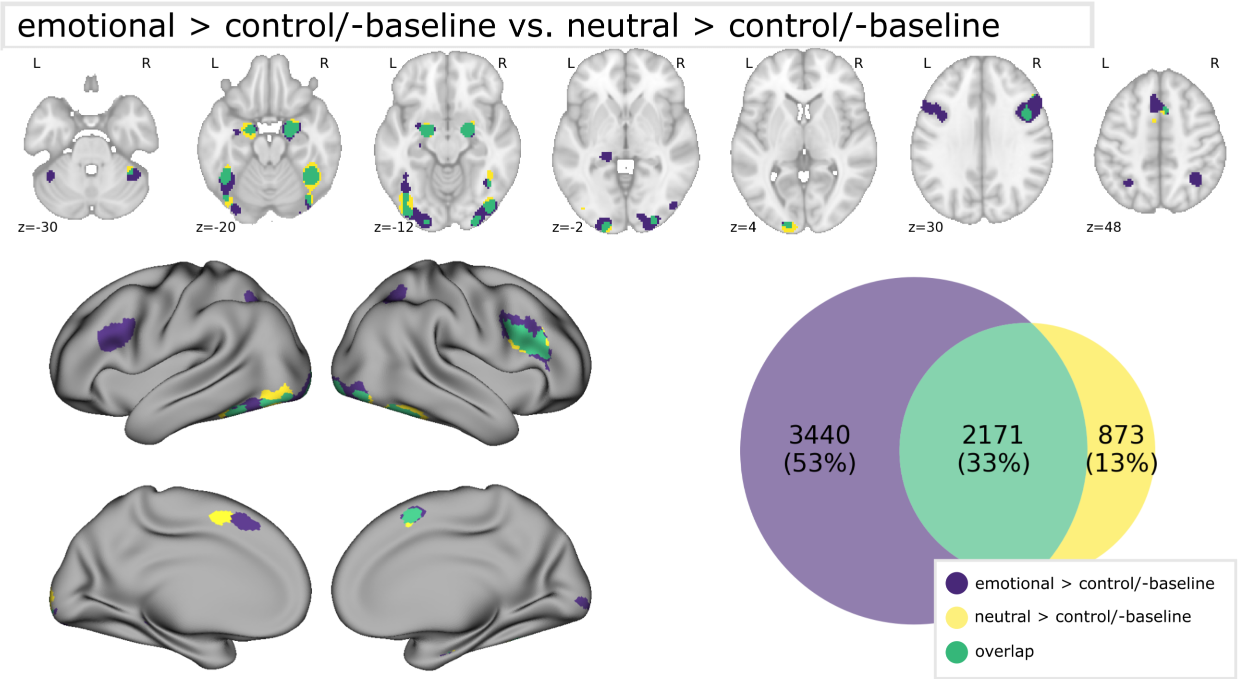

Supplementary Figure S2.5: Emotional > control/-baseline (52 experiments) vs. neutral > control/-baseline (34 experiments). Individual meta-analysis (cFWE 0.05 corrected) used for computation of meta-analytic contrast between two meta-analyses (MC) overlayed on each other.

3 Supplement: datasets

3.1 Working memory datasets

Supplementary Table S3.1: The two Working Memory (2-back > 0-back; 2-back > 1-back) datasets

	N	Space	Contrast	Task	Stimuli	Source	Sub-analysis
Jiang et al., 2015 - group 1	20	MNI	2-back > 0-back	n-back	numbers	from publication	
Jiang et al., 2015 - group 2	20	MNI	2-back > 0-back	n-back	numbers	from publication	
Aguilar-Ortiz et al., 2019	67	MNI	2-back > baseline	n-back	letter	from publication	matched (2>0)

	N	Space	Contrast	Task	Stimuli	Source	Sub-analysis
	67	MNI	2-back > 1-back	n-back	letter	from publication	matched (2>1)
Aguirre et al., 2019	29	MNI	2-back > 0-back	n-back	letter	sent by authors	
Alain et al., 2010	12	TAL	2-back > 1-back	n-back	sound	from publication	matched (2>1)
Alain et al., 2018	41	MNI	2-back > 1-back	n-back	sound	from publication	matched (2>1)
Allen et al., 2006	10	TAL	2-back > 0-back	n-back	letter	from publication	
Alonso-Lana et al., 2016	28	MNI	2-back > baseline	n-back	letter	from publication	
	28	MNI	2-back > 1-back	n-back	letter	from publication	matched (2>1)
Amann et al., 2011	15	TAL	2-back > baseline	n-back	numbers	from publication	matched (2>0)
Awh et al., 1996	9	TAL	2-back > 0-back	n-back	letter	from publication	matched (2>0)
Bleich-Cohen et al., 2014	20	TAL	2-back > 0-back	n-back	numbers	from publication	matched (2>0)
Boller et al., 2017	32	MNI	2-back > baseline	n-back	letter	from publication	
	32	MNI	2-back > 1-back	n-back	letter	from publication	matched (2>1)
Campanella et al., 2013	16	MNI	2-back > 0-back	n-back	numbers	sent by authors	
Cerasa et al., 2008	30	MNI	2-back > 0-back	n-back	shapes	from publication	
Choo et al., 2005	12	TAL	2-back > 1-back	n-back	letter	from publication	matched (2>1)
Ciesielski et al., 2006	10	TAL	2-back > baseline	n-back	figures	from publication	
Clark et al., 2017	63	MNI	2-back > 1-back	n-back	shapes/ letter	from publication	matched (2>1)
Daamen et al., 2015 - group 1	73	MNI	2-back > baseline	n-back	letter	sent by authors	matched (2>0)
	73	MNI	0-back > baseline	n-back	letter	sent by authors	matched (2>0)
	73	MNI	2-back > 0-back	n-back	letter	sent by authors	matched (2>0)

	N	Space	Contrast	Task	Stimuli	Source	Sub-analysis
Daamen et al., 2015 - group 2	73	MNI	2-back > 0-back	n-back	letter	sent by authors	matched (2>0)
	73	MNI	2-back > baseline	n-back	letter	sent by authors	matched (2>0)
	73	MNI	0-back > baseline	n-back	letter	sent by authors	matched (2>0)
Deckersbach et al., 2008	17	MNI	2-back > baseline	n-back	letter	from publication	
Dima et al., 2014	40	MNI	2-back > 0-back	n-back	letter	from publication	matched (2>0, 2>1)
	40	MNI	1-back > 0-back	n-back	letter	from publication	matched (2>1)
Doehnel et al., 2008	16	MNI	2-back > baseline	n-back	figures	from publication	
Dores et al., 2017	10	TAL	2-back > baseline	n-back	shapes	from publication	matched (2>0)
Drapier et al., 2008	20	TAL	1-back > 0-back	n-back	letter	from publication	matched (2>1)
	20	TAL	2-back > 0-back	n-back	letter	from publication	matched (2>1)
Duggirala et al., 2016	50	MNI	2-back > 0-back	n-back	words	from publication	
Esteves et al., 2018	22	MNI	2-back > 0-back	n-back	letter	from publication	matched (2>1)
	31	MNI	1-back > 0-back	n-back	letter	from publication	matched (2>1)
Fernández-Corcuera et al., 2013	41	MNI	2-back > baseline	n-back	letter	from publication	matched (2>0)
Forn et al., 2007	10	TAL	2-back > 0-back	n-back	letter	from publication	matched (2>1)
Fuentes-Claramonte et al., 2019	36	MNI	2-back > 1-back	n-back	letter	from neurovault	matched (2>1)
	36	MNI	2-back > baseline	n-back	letter	from publication	matched (2>0)
Fuentes-Claramonte et al., 2021	70	MNI	2-back > baseline	n-back	letter	from publication	
Fukuda et al., 2019 - group 1	24	MNI	2-back > baseline	n-back	numbers	sent by authors	matched (2>0)
	24	MNI	0-back > baseline	n-back	numbers	sent by authors	matched (2>0)

	N	Space	Contrast	Task	Stimuli	Source	Sub-analysis
	24	MNI	2-back > 0-back	n-back	numbers	sent by authors	matched (2>0)
Fukuda et al., 2019 - group 2	24	MNI	0-back > baseline	n-back	numbers	sent by authors	matched (2>0)
	24	MNI	2-back > baseline	n-back	numbers	sent by authors	matched (2>0)
	24	MNI	2-back > 0-back	n-back	numbers	sent by authors	matched (2>0)
Garrett et al., 2011	19	TAL	1-back > 0-back	n-back	letter	from publication	matched (2>1)
	19	TAL	2-back > 0-back	n-back	letter	from publication	matched (2>1)
Gillis et al., 2016	15	TAL	2-back > 0-back	n-back	letter/ visuospatial	from publication	matched (2>1)
Goikolea et al., 2019	31	TAL	2-back > baseline	n-back	letter	from publication	
Gropman et al., 2013	21	MNI	2-back > 1-back	n-back	letter	from publication	matched (2>1)
Habel et al., 2007	21	MNI	2-back > 0-back	n-back	letter	from publication	
Habel et al., 2007 - Koch et al., 2007	47	MNI	0-back > baseline	n-back	letter	sent by authors	matched (2>0)
Harding et al., 2016	34	MNI	2-back > 0-back	n-back	letter	sent by authors	matched (2>0)
	34	MNI	2-back > baseline	n-back	letter	sent by authors	matched (2>0)
	34	MNI	0-back > baseline	n-back	letter	sent by authors	matched (2>0)
Heinzel et al., 2016	29	MNI	2-back > baseline	n-back	numbers	from publication	matched (2>0)
	29	MNI	0-back > baseline	n-back	numbers	from publication	matched (2>0)
Honey et al., 2000	20	TAL	2-back > 0-back	n-back	letter	from publication	
Honey et al., 2003	27	TAL	2-back > 0-back	n-back	letter	from publication	
Huang et al., 2016	18	TAL	1-back > 0-back	n-back	shapes	from publication	matched (2>1)
	18	TAL	2-back > 1-back	n-back	shapes	from publication	matched (2>1)

	N	Space	Contrast	Task	Stimuli	Source	Sub-analysis
J.-W. Park et al., 2016	45	MNI	2-back > 0-back	n-back	shapes	from publication	
Jablonska et al., 2020	41	MNI	2-back > 0-back	n-back	sound	from publication	matched (2>1)
	41	MNI	2-back > 1-back	n-back	sound	from publication	matched (2>1)
	41	MNI	1-back > 0-back	n-back	sound	from publication	matched (2>1)
Johannsen et al., 2013	12	MNI	2-back > baseline	n-back	letter	from publication	
Jonides et al., 1997	18	TAL	0-back > baseline	n-back	letter	from publication	matched (2>0)
	18	TAL	2-back > baseline	n-back	letter	from publication	matched (2>0)
Jung et al., 2018	24	MNI	2-back > 0-back	n-back	numbers	sent by authors	
Kaminski et al., 2019	41	MNI	2-back > 0-back	n-back	numbers	sent by authors	matched (2>0)
	41	MNI	2-back > baseline	n-back	numbers	sent by authors	matched (2>0)
	41	MNI	0-back > baseline	n-back	numbers	sent by authors	matched (2>0)
Kaur et al., 2022 - group 1	10	MNI	2-back > baseline	n-back	letter	from publication	
Kaur et al., 2022 - group 2	12	MNI	2-back > baseline	n-back	letter	from publication	
Kaur et al., 2022 - group 3	11	MNI	2-back > baseline	n-back	letter	from publication	
Kim et al., 2002	14	TAL	2-back > 0-back	n-back	shapes	from publication	
Kim et al., 2003	12	TAL	2-back > 0-back	n-back	shapes	from publication	
Kim et al., 2006	12	TAL	2-back > baseline	n-back	letter	from publication	
King et al., 2015	17	MNI	2-back > 0-back	n-back	letter	sent by authors	matched (2>0)
	17	MNI	0-back > baseline	n-back	letter	sent by authors	matched (2>0)
Knops et al., 2006	13	TAL	2-back > 1-back	n-back	letter/ numbers	from publication	matched (2>1)

	N	Space	Contrast	Task	Stimuli	Source	Sub-analysis
Koppelstaetter et al., 2008	15	TAL	2-back > 0-back	n-back	letter	from publication	
Korsnes et al., 2013	11	MNI	2-back > 1-back	n-back	numbers	from publication	matched (2>1)
Kowalczyk et al., 2020	20	MNI	1-back > 0-back	n-back	letter	sent by authors	matched (2>1)
	20	MNI	2-back > 0-back	n-back	letter	sent by authors	matched (2>1)
	20	MNI	2-back > 1-back	n-back	letter	sent by authors	matched (2>1)
Krug et al., 2008	85	MNI	0-back > baseline	n-back	letter	sent by authors	matched (2>0)
Kumari et al., 2006	13	TAL	1-back > 0-back	n-back	shapes	from publication	matched (2>1)
	13	TAL	2-back > 0-back	n-back	shapes	from publication	matched (2>0, 2>1)
	13	TAL	0-back > baseline	n-back	shapes	from publication	matched (2>0)
L. Li et al., 2019	24	MNI	2-back > baseline	n-back	letter	sent by authors	matched (2>0)
	24	MNI	2-back > 0-back	n-back	letter	sent by authors	matched (2>0, 2>1)
	24	MNI	2-back > 1-back	n-back	letter	sent by authors	matched (2>1)
	24	MNI	0-back > baseline	n-back	letter	sent by authors	matched (2>0)
Lahr et al., 2018	83	MNI	2-back > 1-back	n-back	letter	sent by authors	matched (2>1)
	83	MNI	1-back > 0-back	n-back	letter	sent by authors	matched (2>1)
	83	MNI	2-back > 0-back	n-back	letter	sent by authors	matched (2>0, 2>1)
Leung and Alain 2011	16	TAL	2-back > 1-back	n-back	sound	from publication	matched (2>1)
Li et al., 2014	15	MNI	0-back > baseline	n-back	letter	from publication	matched (2>0)
	15	MNI	2-back > baseline	n-back	letter	from publication	matched (2>0)
Luo et al., 2014	25	MNI	2-back > 0-back	n-back	faces	from publication	

	N	Space	Contrast	Task	Stimuli	Source	Sub-analysis
Malisza et al., 2005	6	MNI	1-back > 0-back	n-back	shapes	from publication	matched (2>1)
Manktelow et al., 2017	15	MNI	2-back > 0-back	n-back	letter	from publication	matched (2>0)
Marquand et al., 2008	20	MNI	2-back > 0-back	n-back	letter	from publication	
Matsuo et al., 2007	15	TAL	2-back > 0-back	n-back	shapes	from publication	matched (2>1)
	15	TAL	1-back > 0-back	n-back	numbers	from publication	matched (2>1)
McAllister et al., 1999	11	MNI	1-back > 0-back	n-back	letter	from publication	matched (2>1)
	11	MNI	2-back > 1-back	n-back	letter	from publication	matched (2>1)
McGeown et al., 2008	9	TAL	1-back > 0-back	n-back	words	from publication	matched (2>1)
Meisenzahl et al., 2006	12	MNI	2-back > 0-back	n-back	letter	from publication	matched (2>1)
Migo et al., 2014	11	MNI	1-back > 0-back	n-back	letter	from publication	matched (2>1)
	11	MNI	2-back > 0-back	n-back	letter	from publication	matched (2>1)
Miró-Padilla et al., 2019	52	MNI	2-back > 0-back	n-back	letter	sent by authors	matched (2>0)
Monks et al., 2004	12	TAL	2-back > 0-back	n-back	letter	from publication	
Park et al., 2011	10	MNI	2-back > 0-back	n-back	letter	from publication	
Pfefferbaum et al., 2001	10	TAL	0-back > baseline	n-back	letter	from publication	matched (2>0)
	10	TAL	2-back > baseline	n-back	letter	from publication	matched (2>0)
	10	TAL	2-back > 0-back	n-back	letter	from publication	matched (2>0)
Philip et al., 2016	13	TAL	2-back > baseline	n-back	letter	from publication	matched (2>0)
	13	TAL	0-back > baseline	n-back	letter	from publication	matched (2>0)
Pomarol-Clotet et al., 2008	32	TAL	2-back > baseline	n-back	letter	from publication	

	N	Space	Contrast	Task	Stimuli	Source	Sub-analysis
Qin et al., 2009	27	MNI	2-back > 0-back	n-back	numbers	from publication	matched (2>0)
Ragland et al., 2002	11	TAL	2-back > 0-back	n-back	letter	from publication	matched (2>1)
	11	TAL	1-back > 0-back	n-back	letter	from publication	matched (2>1)
	11	TAL	2-back > 1-back	n-back	letter	from publication	matched (2>1)
Rama et al., 2001	8	TAL	2-back > 0-back	n-back	words	from publication	matched (2>1)
	8	TAL	1-back > 0-back	n-back	words	from publication	matched (2>1)
Richter et al., 2013	34	MNI	2-back > 0-back	n-back	faces	from publication	
Rodriguez-Cano et al., 2014	52	MNI	2-back > baseline	n-back	letter	from publication	matched (2>0)
Rodriguez-Cano et al., 2017	26	MNI	2-back > baseline	n-back	letter	from publication	
Salavert et al., 2018	41	MNI	2-back > baseline	n-back	letter	from publication	
Sapara et al., 2014	20	MNI	1-back > 0-back	n-back	shapes	from publication	matched (2>1)
	20	MNI	2-back > baseline	n-back	shapes	from publication	matched (2>0)
	20	MNI	0-back > baseline	n-back	shapes	from publication	matched (2>0)
	20	MNI	2-back > 0-back	n-back	shapes	from publication	matched (2>0, 2>1)
Scheller et al., 2017	34	MNI	2-back > 0-back	n-back	letter	from publication	matched (2>1)
	34	MNI	1-back > 0-back	n-back	letter	from publication	matched (2>1)
Scheuerecker et al., 2008	23	MNI	2-back > 0-back	n-back	letter	from publication	
Schlagenhauf et al., 2008	10	MNI	0-back > baseline	n-back	numbers	sent by authors	matched (2>0)
Schmidt et al., 2015	32	MNI	2-back > 0-back	n-back	letter	from publication	
Schneider et al., 2007	81	MNI	0-back > baseline	n-back	letter	sent by authors	matched (2>0)

	N	Space	Contrast	Task	Stimuli	Source	Sub-analysis
Schneiders et al., 2011	48	TAL	2-back > 0-back	n-back	shapes	from publication	
Seo et al., 2012	22	MNI	2-back > 0-back	n-back	letter	from publication	
Seo et al., 2014	34	MNI	2-back > 0-back	n-back	letter	from publication	
Smits et al., 2009	12	MNI	0-back > baseline	n-back	numbers	sent by authors	
Spreng et al., 2014	36	MNI	2-back > baseline	n-back	faces	from publication	
Stoodley et al., 2012	9	MNI	2-back > 0-back	n-back	letter	from publication	
Sánchez-Carrión et al., 2008	14	MNI	2-back > 0-back	n-back	numbers	from publication	
Takeuchi et al., 2018	1235	MNI	2-back > 0-back	n-back	letter	sent by authors	matched (2>0)
	1235	MNI	2-back > baseline	n-back	letter	sent by authors	matched (2>0)
	1235	MNI	0-back > baseline	n-back	letter	sent by authors	matched (2>0)
Thornton and Conway, 2013	14	MNI	2-back > 1-back	n-back	faces	from publication	matched (2>1)
Vacchi et al., 2017	24	MNI	2-back > 0-back	n-back	letter	sent by authors	matched (2>1)
	24	MNI	1-back > 0-back	n-back	letter	sent by authors	matched (2>1)
Verhallen et al., 2021	116	MNI	2-back > 0-back	n-back	letter	from publication	matched (2>0, 2>1)
	116	MNI	2-back > 1-back	n-back	letter	from publication	matched (2>1)
	116	MNI	1-back > 0-back	n-back	letter	from publication	matched (2>1)
	116	MNI	2-back > baseline	n-back	letter	from publication	matched (2>0)
	116	MNI	0-back > baseline	n-back	letter	from publication	matched (2>0)
Waiter et al., 2009	37	TAL	2-back > 0-back	n-back	letter	from publication	
Walitt et al., 2016	13	MNI	2-back > 0-back	n-back	letter	from publication	matched (2>0)

	N	Space	Contrast	Task	Stimuli	Source	Sub-analysis
Wesley et al., 2017	11	MNI	1-back > 0-back	n-back	letter	from publication	matched (2>1)
	11	MNI	2-back > 1-back	n-back	letter	from publication	matched (2>1)
Wishart et al., 2006	22	MNI	2-back > 0-back	n-back	letter	from publication	
Wu et al., 2017	45	MNI	2-back > baseline	n-back	numbers	sent by authors	matched (2>0)
	45	MNI	0-back > baseline	n-back	numbers	sent by authors	matched (2>0)
	45	MNI	2-back > 0-back	n-back	numbers	from publication	matched (2>0)
X. Li et al., 2019	24	MNI	2-back > 0-back	n-back	shapes/ letter	from publication	
Yan et al., 2011 - group 1	28	TAL	2-back > 0-back	n-back	shapes	from publication	matched (2>1)
Yan et al., 2011 - group 2	28	TAL	2-back > 0-back	n-back	shapes	from publication	
Yang et al., 2018	24	MNI	2-back > 0-back	n-back	letter	from publication	
Yoo et al., 2005	10	MNI	2-back > 0-back	n-back	faces	from publication	
Ziemus et al., 2007	9	TAL	2-back > 0-back	n-back	letter	from publication	
Zurowski et al., 2002	8	MNI	2-back > 0-back	n-back	syllable	from publication	
van der Horn et al., 2016	20	MNI	2-back > 1-back	n-back	letter	sent by authors	matched (2>1)
	20	MNI	2-back > 0-back	n-back	letter	sent by authors	matched (2>1)
	20	MNI	1-back > 0-back	n-back	letter	sent by authors	matched (2>1)

3.2 Interference processing dataset

Supplementary Table S3.2: Interference processing dataset

Studies	N	Space	Contrast	Task	Stimuli	Source	Sub-analysis
Adleman et al., 2002	11	TAL	incongruent > neutral	colour naming	colour-words	from publication	

Studies	N	Space	Contrast	Task	Stimuli	Source	Sub-analysis
Agostini et al 2017	17	MNI	incongruent > congruent	colourword matching	colour-words	sent by authors	matched
	17	MNI	congruent > baseline	colourword matching	colour-words	sent by authors	matched
	17	MNI	incongruent > baseline	colourword matching	colour-words	sent by authors	matched
Banich et al., 2001	14	TAL	incongruent > neutral	colour naming	colour-words	from publication	
Barkley-Levenson et al., 2018	105	MNI	incongruent > neutral	colour naming	colour-words	from publication	matched
	105	MNI	congruent > baseline	colour naming	colour-words	from publication	matched
	105	MNI	incongruent > congruent	colour naming	colour-words	from publication	matched
	105	MNI	incongruent > baseline	colour naming	colour-words	from publication	matched
Basten et al., 2011	46	MNI	incongruent > congruent	colour naming	colour-words	from publication	
Becker et al., 2008	17	TAL	incongruent > congruent	colour naming	colour-words	from publication	
Bench et al., 1993 - group 1	6	TAL	incongruent > neutral	colour naming	colour-words	from publication	
Bench et al., 1993 - group 2	6	TAL	incongruent > neutral	colour naming	colour-words	from publication	matched
	6	TAL	congruent > neutral	colour naming	colour-words	from publication	matched
Brass et al., 2005	10	TAL	incongruent > congruent	colour naming	colour-words	from publication	matched
Carter et al., 1995	14	TAL	incongruent > congruent	colour naming	colour-words	from publication	matched
	14	TAL	incongruent > neutral	colour naming	colour-words	from publication	matched
	14	TAL	congruent > neutral	colour naming	colour-words	from publication	matched
Chen et al., 2018	36	MNI	incongruent > congruent	colour naming	colour-words	from publication	
Coderre et al., 2008	9	TAL	incongruent > congruent	colour naming	colour-words	from publication	
	9	TAL	incongruent > neutral	colour naming	colour-words	from publication	
Coderre et al., 2013	14	MNI	incongruent > neutral	colour naming	colour-words	from publication	

Studies	N	Space	Contrast	Task	Stimuli	Source	Sub-analysis
	14	MNI	incongruent > congruent	colour naming	colour-words	from publication	
DeVito et al., 2012	12	MNI	incongruent > congruent	colour naming	colour-words	from publication	
Fan et al., 2003	12	MNI	incongruent > congruent	colour naming	colour-words	from publication	
Fechir et al., 2010	16	MNI	incongruent > congruent	colour naming	colour-words	from publication	
George et al., 1994	21	TAL	incongruent > neutral	colour naming	colour-words	from publication	
Ghavidel et al., 2020	18	MNI	congruent > baseline	colour naming	colour-words	sent by authors	matched
	18	MNI	incongruent > baseline	colour naming	colour-words	sent by authors	matched
	18	MNI	incongruent > congruent	colour naming	colour-words	sent by authors	matched
Gianaros et al., 2008	32	MNI	incongruent > congruent	colour naming	colour-words	from publication	
Grandjean et al., 2013, 2012	25	MNI	incongruent > congruent	colour naming	colour-words	from publication	
	25	MNI	incongruent > neutral	colour naming	colour-words	from publication	
Hough et al., 2016	24	MNI	incongruent > congruent	colour naming	colour-words	from publication	
Huang et al., 2017	33	MNI	incongruent > neutral	colour naming	colour-words	from publication	matched
Jaspar et al., 2014	45	MNI	incongruent > neutral	colour naming	colour-words	from publication	
Jeong et al., 2005	10	MNI	incongruent > congruent	colourword matching	colour-words	from publication	
Kerns et al., 2005	13	TAL	incongruent > congruent	colour naming	colour-words	from publication	matched
Kim et al., 2014	18	MNI	incongruent > congruent	colourword matching	colour-words	from publication	
Kozasa et al., 2018	33	MNI	congruent > neutral	colour naming	colour-words	sent by authors	matched
	33	MNI	incongruent > congruent	colour naming	colour-words	sent by authors	matched
Kronhaus et al., 2006	11	TAL	incongruent > neutral	colour naming	colour-words	from publication	
Köhler et al., 2016	45	MNI	incongruent > baseline	colourword matching	colour-words	sent by authors	matched

Studies	N	Space	Contrast	Task	Stimuli	Source	Sub-analysis
	45	MNI	incongruent > congruent	colourword matching	colour-words	from publication	matched
	45	MNI	congruent > baseline	colourword matching	colour-words	sent by authors	matched
Kühn et al., 2016	19	MNI	incongruent > congruent	colour naming	colour-words	from publication	
Lesh et al., 2013	54	MNI	incongruent > congruent	colour naming	colour-words	from publication	
Li et al., 2019	20	MNI	incongruent > congruent	colour naming	colour-words	from publication	
Luethi et al., 2016	88	MNI	incongruent > baseline	colourword matching	colour-words	sent by authors	matched
	88	MNI	congruent > baseline	colourword matching	colour-words	sent by authors	matched
	88	MNI	incongruent > congruent	colourword matching	colour-words	from publication	matched
Manard et al., 2017	40	MNI	incongruent > neutral	colour naming	colour-words	from publication	
Mathis et al., 2009 - group 1	12	MNI	congruent > neutral	colourword matching	colour-words	from publication	matched
	12	MNI	incongruent > congruent	colourword matching	colour-words	from publication	matched
	12	MNI	incongruent > neutral	colourword matching	colour-words	from publication	matched
Mathis et al., 2009 - group 2	12	MNI	incongruent > congruent	colourword matching	colour-words	from publication	matched
	12	MNI	incongruent > neutral	colourword matching	colour-words	from publication	matched
	12	MNI	congruent > neutral	colourword matching	colour-words	from publication	matched
Mathis et al., 2009 - group 3	12	MNI	incongruent > neutral	colourword matching	colour-words	from publication	matched
	12	MNI	congruent > neutral	colourword matching	colour-words	from publication	matched
Mead et al., 2002	18	TAL	incongruent > congruent	colour naming	colour-words	from publication	
	18	TAL	incongruent > neutral	colour naming	colour-words	from publication	
Milham et al., 2001	16	TAL	incongruent > neutral	colour naming	colour-words	from publication	
Mitchell, 2005	13	TAL	incongruent > neutral	colour naming	colour-words	from publication	matched

Studies	N	Space	Contrast	Task	Stimuli	Source	Sub-analysis
	13	TAL	congruent > neutral	colour naming	colour-words	from publication	matched
Morgenroth et al., 2019	39	MNI	incongruent > congruent	colour naming	colour-words	from publication	
Nakao et al., 2005	14	TAL	incongruent > congruent	colour naming	colour-words	from publication	
Ning et al., 2021	19	MNI	incongruent > congruent	colour naming	colour-words	from publication	
Norris et al., 2002	7	TAL	incongruent > neutral	colourword matching	colour-words	from publication	
Overbeek et al., 2019	21	MNI	incongruent > congruent	colour naming	colour-words	from publication	
Papalini et al., 2018	58	MNI	incongruent > congruent	colour naming	colour-words	sent by authors	
Pardo et al., 1990	8	TAL	incongruent > congruent	colour naming	colour-words	from publication	matched
Peven et al., 2019	50	MNI	incongruent > baseline	colour naming	colour-words	sent by authors	matched
	50	MNI	congruent > baseline	colour naming	colour-words	sent by authors	matched
	50	MNI	incongruent > congruent	colour naming	colour-words	sent by authors	matched
	50	MNI	incongruent > neutral	colour naming	colour-words	sent by authors	matched
Piai et al., 2013	23	MNI	incongruent > congruent	colour naming	colour-words	from publication	
	23	MNI	incongruent > neutral	colour naming	colour-words	from publication	
Polk et al., 2008	14	TAL	incongruent > neutral	colour naming	colour-words	from publication	
Pompei et al., 2011	48	TAL	incongruent > neutral	colour naming	colour-words	from publication	
Portes et al., 2019 - group 1	19	MNI	incongruent > congruent	colour naming	colour-words	from publication	
Portes et al., 2019 - group 2	21	MNI	incongruent > congruent	colour naming	colour-words	from publication	
Potenza et al., 2003	11	TAL	incongruent > congruent	colour naming	colour-words	from publication	matched
Prakash et al., 2009 - group 1	25	MNI	incongruent > neutral	colour naming	colour-words	from publication	
Prakash et al., 2009 - group 2	25	MNI	incongruent > neutral	colour naming	colour-words	from publication	

Studies	N	Space	Contrast	Task	Stimuli	Source	Sub-analysis
Purmann and Pollmann, 2015	18	MNI	incongruent > congruent	colourword matching	colour-words	from publication	
Ravnkilde et al., 2002	46	TAL	incongruent > congruent	colour naming	colour-words	from publication	
Roberts and Hall, 2008	16	MNI	incongruent > neutral	colour naming	colour-words	from publication	matched
	16	MNI	congruent > neutral	colour naming	colour-words	from publication	matched
Ruff et al., 2001	12	MNI	incongruent > neutral	colour naming	colour-words	from publication	
Schmidt et al., 2012	31	MNI	incongruent > baseline	colour naming	colour-words	sent by authors	matched
	31	MNI	incongruent > neutral	colour naming	colour-words	sent by authors	matched
	31	MNI	congruent > neutral	colour naming	colour-words	sent by authors	matched
	31	MNI	congruent > baseline	colour naming	colour-words	sent by authors	matched
	31	MNI	incongruent > congruent	colour naming	colour-words	sent by authors	matched
Schulte et al., 2009	24	MNI	incongruent > congruent	colourword matching	colour-words	from publication	
Schulte et al., 2012	17	MNI	incongruent > congruent	colourword matching	colour-words	from publication	
Shashidhara et al., 2020	18	MNI	incongruent > congruent	colourword matching	colour-words	sent by authors	
Sheu et al., 2012	138	MNI	congruent > baseline	colour naming	colour-words	from publication	matched
	138	MNI	incongruent > congruent	colour naming	colour-words	from publication	matched
	138	MNI	incongruent > baseline	colour naming	colour-words	from publication	matched
Shin and Kim, 2015	43	MNI	incongruent > congruent	colourword matching	colour-words	from publication	
Silton et al., 2010	30	MNI	incongruent > congruent	colour naming	colour-words	from publication	
Song et al., 2015	20	MNI	incongruent > neutral	colourword matching	colour-words	from publication	
Steel et al., 2001	7	TAL	incongruent > neutral	colour naming	colour-words	from publication	matched
	7	TAL	congruent > neutral	colour naming	colour-words	from publication	matched

Studies	N	Space	Contrast	Task	Stimuli	Source	Sub-analysis
Taylor et al., 1997 - group 1	12	TAL	incongruent > neutral	colour naming	colour-words	from publication	
Taylor et al., 1997 - group 2	6	TAL	incongruent > neutral	colour naming	colour-words	from publication	
Terry et al., 2012	20	MNI	incongruent > congruent	colour naming	colour-words	from publication	
Van't Ent et al., 2014	45	MNI	incongruent > congruent	colour naming	colour-words	from publication	
Veroude et al., 2013	74	MNI	incongruent > neutral	colour naming	colour-words	from publication	
Verstynen et al., 2014	28	MNI	incongruent > neutral	colour naming	colour-words	from publication	
Wagner et al., 2016	40	MNI	incongruent > congruent	colourword matching	colour-words	sent by authors	matched
	40	MNI	incongruent > baseline	colourword matching	colour-words	sent by authors	matched
	40	MNI	congruent > baseline	colourword matching	colour-words	sent by authors	matched
Wallentin et al., 2015	49	MNI	congruent > baseline	colour naming	colour-words	sent by authors	matched
	49	MNI	incongruent > baseline	colour naming	colour-words	sent by authors	matched
	49	MNI	incongruent > congruent	colour naming	colour-words	sent by authors	matched
Ye et al., 2009	19	MNI	congruent > neutral	colour naming	colour-words	sent by authors	matched
	19	MNI	congruent > baseline	colour naming	colour-words	sent by authors	matched
	19	MNI	incongruent > neutral	colour naming	colour-words	sent by authors	matched
	19	MNI	incongruent > baseline	colour naming	colour-words	sent by authors	matched
	19	MNI	incongruent > congruent	colour naming	colour-words	from publication	matched
Zhu et al., 2013	26	MNI	incongruent > congruent	colour naming	colour-words	from publication	
Zoccatelli et al., 2010	10	TAL	incongruent > congruent	colour naming	colour-words	from publication	matched
Zysset et al., 2001	9	TAL	incongruent > neutral	colourword matching	colour-words	from publication	matched
	9	TAL	congruent > neutral	colourword matching	colour-words	from publication	matched

Studies	N	Space	Contrast	Task	Stimuli	Source	Sub-analysis
	9	TAL	incongruent > congruent	colourword matching	colour-words	from publication	matched
Zysset et al., 2007	47	TAL	incongruent > neutral	colourword matching	colour-words	from publication	
van de Meerendonk et al., 2013	24	MNI	incongruent > neutral	colour naming	colour-words	from publication	

164 3.3 Emotional face processing dataset

165 *Supplementary Table S3.3: Emotional faces dataset*

Studies	N	Space	Contrast	Task (condition of interest)	Task (contrasting condition)	Stimuli (condition of interest)	Stimuli (contrasting condition)	Source	Sub-analysis
Bas-Hoogendam et al., 2015	21	MNI	emotional > control	gender-discrimination	arrow-location	anger, fear, happiness, sadness	scrambled images	from publication	matched
Batut et al., 2006	15	TAL	emotional > neutral	gender-discrimination	gender-discrimination	fear	neutral faces	from publication	matched
	15	TAL	emotional > neutral	gender-discrimination	gender-discrimination	happiness	neutral faces	from publication	matched
	15	TAL	emotional > neutral	gender-discrimination	gender-discrimination	sadness	neutral faces	from publication	matched
Benuzzi et al., 2004	14	TAL	emotional > neutral	gender-discrimination	gender-discrimination	fear	neutral faces	from publication	
Benuzzi et al., 2007	24	TAL	neutral > control	gender-discrimination	pick-white-square	neutral	scrambled images	from publication	matched
Binelli et al., 2015	20	MNI	emotional > control	emotion-matching	shape-matching	happiness, anger, fear	shapes (ovals, circles)	from publication	
Bird et al., 2006	16	MNI	neutral > control	passive-viewing	passive-viewing	neutral	houses	from publication	
Borgsted et al., 2018	15	MNI	emotional > neutral	gender-discrimination	gender-discrimination	anger, fear	neutral faces	from publication	matched
Briceno et al., 2013	22	TAL	emotional > control	emotion-discrimination	animal-discrimination	anger, fear, happiness, sadness	animals	from publication	
Brown et al., 2006	58	MNI	emotional > control	identity-matching	shape-matching	anger, fear	shapes (circles, ellipses)	from publication	

Studies	N	Space	Contrast	Task (condition of interest)	Task (contrasting condition)	Stimuli (condition of interest)	Stimuli (contrasting condition)	Source	Sub-analysis
Bukowski et al., 2013	11	TAL	neutral > control	identity-one-back	identity-one-back	neutral	scrambled images, cars	from publication	matched
Cardoner et al., 2011	21	MNI	emotional > control	emotion-matching	shape-matching	fear	shapes (ovals, circles)	from publication	
	21	MNI	emotional > control	emotion-matching	shape-matching	happiness, fear	shapes (ovals, circles)	from publication	
	21	MNI	emotional > control	emotion-matching	shape-matching	happiness	shapes (ovals, circles)	from publication	
Carvajal et al., 2013	20	TAL	emotional > neutral	gender-discrimination, social-task	gender-discrimination_and_social-task	anger, happiness, sadness	neutral faces	from publication	
Contreras-Rodriguez et al., 2014	22	MNI	emotional > control	emotion-matching	shape-matching	fear, happiness	shapes (ovals, circles)	from publication	
	22	MNI	emotional > control	emotion-matching	shape-matching	fear	shapes (ovals, circles)	from publication	
	22	MNI	emotional > control	emotion-matching	shape-matching	happiness	shapes (ovals, circles)	from publication	
Critchley et al., 2005	15	MNI	emotional > baseline	emotion-discrimination	-	anger, disgust, happiness, sadness	implicit	from publication	
Cullen et al., 2016	12	MNI	emotional > baseline	active-viewing	active-viewing	fear	fixation cross	sent by authors	
Dan et al., 2019	32	MNI	emotional > control	emotion-matching	shape-matching	anger, fear	shapes (ovals)	sent by authors	
Deeley et al., 2007	9	TAL	emotional > baseline	gender-discrimination	-	sadness	fixation cross	from publication	matched
	9	TAL	emotional > baseline	gender-discrimination	-	fear	fixation cross	from publication	matched
	9	TAL	emotional > baseline	gender-discrimination	-	disgust	fixation cross	from publication	matched
	9	TAL	emotional > baseline	gender-discrimination	-	happiness	fixation cross	from publication	matched
	9	TAL	neutral > baseline	gender-discrimination	-	neutral	fixation cross	from publication	matched

Studies	N	Space	Contrast	Task (condition of interest)	Task (contrasting condition)	Stimuli (condition of interest)	Stimuli (contrasting condition)	Source	Sub-analysis
Demenescu et al., 2011	56	MNI	neutral > control	gender-discrimination	arrow-location	neutral	scrambled images	from publication	matched
	56	MNI	emotional > control	gender-discrimination	arrow-location	fear	scrambled images	from publication	matched
	56	MNI	emotional > neutral	gender-discrimination	gender-discrimination	anger, fear, happiness, sadness	neutral faces	from publication	matched
	56	MNI	emotional > control	gender-discrimination	arrow-location	sadness	scrambled images	from publication	matched
	56	MNI	emotional > control	gender-discrimination	arrow-location	happiness	scrambled images	from publication	matched
	56	MNI	emotional > control	gender-discrimination	arrow-location	anger	scrambled images	from publication	matched
Dima et al., 2011	40	MNI	emotional > neutral	emotion-discrimination	emotion-discrimination	anger, fear, sadness	neutral faces	from publication	
Dolan et al., 1996	8	MNI	emotional > neutral	identity-matching	identity-matching	happiness	neutral faces	from publication	matched
Dominguez-Borras et al., 2017	19	MNI	emotional > neutral	gender-discrimination	gender-discrimination	fear	neutral faces	from publication	
Engell et al., 2007	12	TAL	emotional > neutral	identity-matching	identity-matching	anger, disgust, fear, surprise	neutral faces	from publication	
Ethofer et al., 2013	23	MNI	neutral > control	identity-one-back	identity-one-back	neutral	houses and scenes	from publication	
Ewbank et al., 2010	27	MNI	emotional > neutral	gender-discrimination	gender-discrimination	anger	neutral faces	from publication	
	27	MNI	emotional > neutral	gender-discrimination	gender-discrimination	fear	neutral faces	from publication	
Fakra et al., 2008	14	MNI	emotional > control	emotion-discrimination	shape-matching	anger, fear	shapes (circles, ellipses)	from publication	
Fitzgerald et al., 2006	20	MNI	emotional > control	emotion-attention	color-attention	anger	radios	from publication	matched
	20	MNI	neutral > control	emotion-attention	color-attention	neutral	radios	from publication	matched
	20	MNI	emotional > control	emotion-attention	color-attention	happiness	radios	from publication	matched

Studies	N	Space	Contrast	Task (condition of interest)	Task (contrasting condition)	Stimuli (condition of interest)	Stimuli (contrasting condition)	Source	Sub-analysis
	20	MNI	emotional > control	emotion-attention	color-attention	sadness	radios	from publication	matched
	20	MNI	emotional > control	emotion-attention	color-attention	fear	radios	from publication	matched
	20	MNI	emotional > control	emotion-attention	color-attention	disgust	radios	from publication	matched
Fraessle et al., 2016	20	MNI	neutral > control	passive-viewing	passive-viewing	neutral	scrambled images	from publication	
Germine et al., 2011	30	MNI	emotional > baseline	emotion-matching	-	happiness, sadness, surprise, fear, disgust, anger	fixation cross	from publication	
Gianaros et al., 2009	36	MNI	emotional > control	emotion-matching	shape-matching	anger, fear	shapes (circles, ellipses)	from publication	
Gianaros et al., 2020	427	MNI	emotional > control	emotion-matching	shape-matching	anger, fear	shapes (circles, ellipses)	from publication	
Grant et al., 2011	16	TAL	emotional > neutral	gender-discrimination	gender-discrimination	positive	neutral faces	from publication	matched
	16	TAL	emotional > neutral	gender-discrimination	gender-discrimination	sadness	neutral faces	from publication	matched
Günther et al., 2020 - group 1	51	MNI	emotional > neutral	color-discrimination	color-discrimination	fear	neutral faces	from publication	
Günther et al., 2020 - group 2	56	MNI	emotional > neutral	color-discrimination	color-discrimination	fear	neutral faces	from publication	
H. Y. Park et al., 2016	17	MNI	neutral > baseline	gender-discrimination	-	neutral	fixation cross	from publication	matched
	17	MNI	emotional > baseline	gender-discrimination	-	fear	fixation cross	from publication	matched
	17	MNI	emotional > baseline	gender-discrimination	-	happiness	fixation cross	from publication	matched
Haas et al., 2009	29	MNI	emotional > neutral	gender-discrimination	gender-discrimination	sadness	neutral faces	from publication	matched
	29	MNI	emotional > neutral	gender-discrimination	gender-discrimination	happiness	neutral faces	from publication	matched
	29	MNI	emotional > neutral	gender-discrimination	gender-discrimination	fear	neutral faces	from publication	matched

Studies	N	Space	Contrast	Task (condition of interest)	Task (contrasting condition)	Stimuli (condition of interest)	Stimuli (contrasting condition)	Source	Sub-analysis
Harada et al., 2020	58	MNI	emotional > control	identity-matching	identity-matching	anger, fear	shapes (ovals, circles)	from publication	matched
Hessl et al., 2007	10	TAL	neutral > control	passive-viewing	passive-viewing	neutral	scrambled images	from publication	matched
	10	TAL	emotional > control	passive-viewing	passive-viewing	fear	scrambled images	from publication	matched
Ihme et al., 2014	48	MNI	emotional > neutral	emotion-discrimination	emotion-discrimination	anger	neutral faces	from publication	matched
	48	MNI	emotional > neutral	emotion-discrimination	emotion-discrimination	fear	neutral faces	from publication	matched
	48	MNI	emotional > neutral	emotion-discrimination	emotion-discrimination	happiness	neutral faces	from publication	matched
Iidaka et al., 2001	12	TAL	emotional > neutral	gender-discrimination	gender-discrimination	anger, disgust	neutral faces	from publication	matched
Iidaka et al., 2002 - group 1	12	MNI	neutral > control	gender-discrimination	size-discrimination	neutral	scrambled images	from publication	matched
	12	MNI	emotional > control	gender-discrimination	size-discrimination	anger, disgust, fear, sadness	scrambled images	from publication	matched
	12	MNI	emotional > control	gender-discrimination	size-discrimination	happiness	scrambled images	from publication	matched
Iidaka et al., 2002 - group 2	12	MNI	emotional > control	gender-discrimination	size-discrimination	anger, disgust, fear, sadness	scrambled images	from publication	matched
	12	MNI	neutral > control	gender-discrimination	size-discrimination	neutral	scrambled images	from publication	matched
	12	MNI	emotional > control	gender-discrimination	size-discrimination	happiness	scrambled images	from publication	matched
Iidaka et al., 2006	12	MNI	neutral > control	face-house-discrimination	face-house-discrimination	neutral	houses	from publication	
Ishitobi et al., 2011	24	MNI	emotional > control	emotion-discrimination	shape-discrimination	anger, disgust, happiness, sadness	shapes_squares-circles	from publication	matched
J.-W. Park et al., 2016	19	MNI	emotional > neutral	emotion-one-back	emotion-one-back	negative	neutral faces	from publication	matched
Jackson et al., 2008	35	TAL	emotional > neutral	identity-matching	identity-matching	anger	neutral faces	from publication	

Studies	N	Space	Contrast	Task (condition of interest)	Task (contrasting condition)	Stimuli (condition of interest)	Stimuli (contrasting condition)	Source	Sub-analysis
Jeong et al., 2011	15	MNI	emotional > baseline	passive-viewing	passive-viewing	sadness	fixation cross	from publication	matched
	15	MNI	emotional > baseline	passive-viewing	passive-viewing	happiness	fixation cross	from publication	matched
Jimura et al., 2009	34	MNI	emotional > neutral	emotion-discrimination	emotion-discrimination	happiness, sadness	neutral faces	from publication	matched
Joassin et al., 2011	12	MNI	neutral > baseline	gender-discrimination	-	neutral	fixation cross	from publication	matched
Jogia et al., 2008	8	MNI	emotional > neutral	emotion-discrimination	emotion-discrimination	sadness	neutral faces	from publication	
Kempton et al., 2009	74	TAL	emotional > neutral	emotion-discrimination	emotion-discrimination	fear	neutral faces	from publication	
Kesler-West et al., 2001	21	TAL	emotional > neutral	emotion-attention	emotion-attention	happiness	neutral faces	from publication	matched
	21	TAL	emotional > neutral	emotion-attention	emotion-attention	anger	neutral faces	from publication	matched
	21	TAL	emotional > neutral	emotion-attention	emotion-attention	fear	neutral faces	from publication	matched
	21	TAL	neutral > control	emotion-attention	passive-viewing	neutral	scrambled images	from publication	matched
	21	TAL	emotional > neutral	emotion-attention	emotion-attention	sadness	neutral faces	from publication	matched
Kilts et al., 2003	13	TAL	emotional > neutral	emotion-intensity-discrimination	spatial-orientation-discrimination	happiness	neutral faces	from publication	
	13	TAL	emotional > neutral	emotion-intensity-discrimination	spatial-orientation-discrimination	anger	neutral faces	from publication	
Kitada et al., 2010	20	MNI	emotional > neutral	emotion-discrimination	emotion-discrimination	happiness	neutral faces	from publication	
	20	MNI	emotional > neutral	emotion-discrimination	emotion-discrimination	disgust	neutral faces	from publication	
Koppe et al., 2015	30	MNI	emotional > control	gender-discrimination	shape-discrimination	happiness	shapes (triangles, squares)	from publication	matched
	30	MNI	emotional > control	gender-discrimination	shape-discrimination	anger	shapes (triangles, squares)	from publication	matched
Kowalczyk et al., 2020	20	MNI	emotional > baseline	gender-discrimination	-	fear	fixation cross	sent by authors	matched
	20	MNI	neutral > baseline	gender-discrimination	-	neutral	fixation cross	sent by authors	matched

Studies	N	Space	Contrast	Task (condition of interest)	Task (contrasting condition)	Stimuli (condition of interest)	Stimuli (contrasting condition)	Source	Sub-analysis
	20	MNI	emotional > neutral	gender-discrimination	gender-discrimination	fear	neutral faces	sent by authors	matched
Kronbichler et al., 2018	31	MNI	neutral > baseline	orientation-discrimination	-	neutral	rest	sent by authors	
	31	MNI	neutral > control	orientation-discrimination	orientation-discrimination	neutral	houses	sent by authors	
Kumari et al., 2015	20	MNI	emotional > control	gender-discrimination	active-viewing	anger	shapes (ovals)	from publication	matched
	20	MNI	emotional > control	gender-discrimination	active-viewing	fear	shapes (ovals)	from publication	matched
	20	MNI	neutral > control	gender-discrimination	active-viewing	neutral	shapes (ovals)	from publication	matched
	20	MNI	emotional > control	gender-discrimination	active-viewing	happiness	shapes (ovals)	from publication	matched
Lee et al., 2008	13	MNI	emotional > neutral	gender-discrimination	gender-discrimination	happiness	neutral faces	from publication	
	13	MNI	emotional > neutral	gender-discrimination	gender-discrimination	sadness	neutral faces	from publication	
Li et al., 2020	14	MNI	emotional > neutral	emotion-discrimination	emotion-discrimination	fear	neutral faces	from publication	
	14	MNI	emotional > neutral	emotion-discrimination	emotion-discrimination	happiness	neutral faces	from publication	
Loven et al., 2013	29	MNI	neutral > baseline	passive-viewing	passive-viewing	neutral	fixation cross	from publication	matched
M. Jehna et al., 2011	29	MNI	neutral > control	gender-discrimination	active-viewing	neutral	scrambled images	from publication	matched
	29	MNI	neutral > control	gender-discrimination	active-viewing	neutral	houses	from publication	matched
	29	MNI	emotional > neutral	gender-discrimination	gender-discrimination	disgust	neutral faces	from publication	matched
	29	MNI	emotional > neutral	gender-discrimination	gender-discrimination	fear	neutral faces	from publication	matched
	29	MNI	emotional > neutral	gender-discrimination	gender-discrimination	anger	neutral faces	from publication	matched
Madsen et al., 2015	76	MNI	emotional > neutral	gender-discrimination	gender-discrimination	anger, fear	neutral faces	from publication	
Malhi et al., 2007	10	TAL	emotional > neutral	emotion-discrimination	emotion-discrimination	disgust	neutral faces	from publication	
	10	TAL	emotional > neutral	emotion-discrimination	emotion-discrimination	fear	neutral faces	from publication	

Studies	N	Space	Contrast	Task (condition of interest)	Task (contrasting condition)	Stimuli (condition of interest)	Stimuli (contrasting condition)	Source	Sub-analysis
Marchand et al., 2011	19	MNI	emotional > neutral	identity-one-back	identity-one-back	happiness	neutral faces	from publication	
Margit Jehna et al., 2011	15	MNI	emotional > neutral	gender-discrimination	gender-discrimination	disgust	neutral faces	from publication	matched
	15	MNI	emotional > neutral	gender-discrimination	gender-discrimination	anger	neutral faces	from publication	matched
	15	MNI	neutral > control	gender-discrimination	active-viewing	neutral	houses	from publication	matched
Maurage et al., 2013	14	MNI	emotional > baseline	emotion-discrimination	-	anger, happiness	fixation cross	from publication	
McCloskey et al., 2016	20	MNI	emotional > neutral	emotion-discrimination	emotion-discrimination	anger	neutral faces	from publication	matched
Mende-Siedlecki et al., 2013	215	MNI	neutral > control	gender-discrimination, identity-one-back	location-discrimination_and_identity-one-back	neutral	chairs, flowers, scenes	from publication	
Meriau et al., 2006	23	MNI	emotional > control	emotion-discrimination	shape-discrimination	anger, fear	shapes_squares-circles	from publication	
Michalopoulou et al., 2008	9	TAL	emotional > neutral	gender-discrimination	gender-discrimination	fear	neutral faces	from publication	
Mier et al., 2010	16	MNI	emotional > baseline	emotion-discrimination	-	anger, fear, happiness(joy)	implicit	from publication	matched
	16	MNI	neutral > baseline	gender-discrimination	-	neutral	implicit	from publication	matched
Miskowiak et al., 2014	17	MNI	emotional > baseline	gender-discrimination	-	fear, happiness	fixation cross	from publication	matched
Morr et al., 2021	54	MNI	emotional > neutral	identity-matching	identity-matching	fear, happiness	neutral faces	from publication	
	54	MNI	emotional > neutral	identity-matching	identity-matching	fear	neutral faces	from publication	
	54	MNI	emotional > neutral	identity-matching	identity-matching	happiness	neutral faces	from publication	
Narumoto et al., 2000	7	TAL	emotional > baseline	matching-emotion	-	fear, happiness, sadness	rest	from publication	
Narumoto et al., 2001	11	MNI	emotional > control	matching-shape, matching-person,	matching-shape	anger, fear, happiness, surprise,	scrambled images	from publication	

Studies	N	Space	Contrast	Task (condition of interest)	Task (contrasting condition)	Stimuli (condition of interest)	Stimuli (contrasting condition)	Source	Sub-analysis
				matching-expression		sadness, disgust			
O'Nions et al., 2011	30	MNI	emotional > neutral	gender-discrimination	gender-discrimination	fear	neutral faces	from publication	matched
	30	MNI	emotional > neutral	gender-discrimination	gender-discrimination	happiness	neutral faces	from publication	matched
Palm et al., 2011	16	MNI	emotional > neutral	gender-discrimination	gender-discrimination	fear	neutral faces	from publication	matched
	16	MNI	emotional > neutral	gender-discrimination	gender-discrimination	anger	neutral faces	from publication	matched
	16	MNI	emotional > neutral	gender-discrimination	gender-discrimination	happiness	neutral faces	from publication	matched
Paradiso et al., 2003	17	TAL	neutral > control	active-viewing	active-viewing	neutral	neutral faces	from publication	matched
Park et al., 2015	16	TAL	emotional > baseline	emotion-intensity-discrimination	-	anger	fixation cross	from publication	
Passamonti et al., 2009	12	MNI	emotional > control	emotion-matching	shape-matching	anger, fear, sadness	shapes (circles, ellipses)	from publication	
Pegors et al., 2015	28	MNI	neutral > control	attractiveness-rating	attractiveness-rating	neutral	places	from publication	
Preckel et al., 2019	27	MNI	emotional > neutral	identity-matching	identity-matching	fear	neutral faces	from publication	
Prochnow et al., 2013	12	TAL	emotional > control	emotion-discrimination	passive-viewing	happiness, anger, sadness	scrambled images	from publication	
Prochnow et al., 2014	26	TAL	emotional > control	empathic-reasoning	passive-viewing	happiness, anger, sadness, fear	scrambled images	from publication	
Reisch et al., 2020	43	MNI	emotional > neutral	passive-viewing	passive-viewing	fear	neutral faces	from publication	
Rosengarth et al., 2021	12	MNI	emotional > control	identity-one-back	identity-one-back	anger, fear, happiness	scrambled images	from publication	matched
	12	MNI	emotional > neutral	identity-one-back	identity-one-back	anger, fear, happiness	neutral faces	from publication	matched
Rossion et al., 2012	40	TAL	neutral > control	identity-one-back	identity-one-back	neutral	cars	from publication	matched
	40	TAL	neutral > control	identity-one-back	identity-one-back	neutral	scrambled images	from publication	matched

Studies	N	Space	Contrast	Task (condition of interest)	Task (contrasting condition)	Stimuli (condition of interest)	Stimuli (contrasting condition)	Source	Sub-analysis
Rubino et al., 2007	28	TAL	emotional > control	emotion-discrimination	shape-matching	anger, fear	shapes (circles, ellipses)	from publication	
Rymarczyk et al., 2019	46	MNI	emotional > neutral	passive-viewing	passive-viewing	fear	neutral faces	from publication	
	46	MNI	emotional > neutral	passive-viewing	passive-viewing	disgust	neutral faces	from publication	
	46	MNI	emotional > neutral	passive-viewing	passive-viewing	fear, disgust	neutral faces	from publication	
Sagaspe et al., 2011	12	MNI	emotional > neutral	gonogo	gonogo	fear	neutral faces	from publication	
Salloum et al., 2007	11	TAL	emotional > baseline	emotion-intensity-discrimination	-	happiness	fixation cross	from publication	matched
	11	TAL	emotional > baseline	emotion-intensity-discrimination	-	disgust	fixation cross	from publication	matched
	11	TAL	emotional > baseline	emotion-intensity-discrimination	-	fear	fixation cross	from publication	matched
	11	TAL	emotional > baseline	emotion-intensity-discrimination	-	anger	fixation cross	from publication	matched
	11	TAL	emotional > baseline	emotion-intensity-discrimination	-	sadness	fixation cross	from publication	matched
Sambataro et al., 2006	24	TAL	emotional > neutral	gender-discrimination	gender-discrimination	disgust	neutral faces	from publication	
	24	TAL	emotional > neutral	gender-discrimination	gender-discrimination	contempt	neutral faces	from publication	
Scheuerecker et al., 2007	12	MNI	emotional > control	gender-matching	shape-matching	anger, sadness	shapes (circles, ellipses, squares, triangles)	from publication	matched
Schultz et al., 2009	10	MNI	emotional > control	identity-one-back	identity-one-back	anger, surprise	scrambled images	from publication	matched
Seara-Cardoso et al., 2015	30	MNI	emotional > neutral	empathic-self-emotion-rating	empathic-self-emotion-rating	sadness, fear, anger, happiness	neutral faces	from publication	
Seitz et al., 2008	14	TAL	emotional > control	identify-emotion, generate-	passive-viewing	happiness, sadness	scrambled images	from publication	

Studies	N	Space	Contrast	Task (condition of interest)	Task (contrasting condition)	Stimuli (condition of interest)	Stimuli (contrasting condition)	Source	Sub-analysis
				emotion, count-earrings					
Snoek et al., 2021 PIOP1	208	MNI	emotional > control	emotion-matching	shape-matching	anger, fear	neutral faces	from neurovault	
Snoek et al., 2021 PIOP2	222	MNI	emotional > control	emotion-matching	shape-matching	anger, fear	neutral faces	from neurovault	
Spilka et al., 2015	27	MNI	neutral > control	passive-viewing	passive-viewing	neutral	scrambled images	from publication	matched
	27	MNI	emotional > control	passive-viewing	passive-viewing	sadness	scrambled images	from publication	matched
	27	MNI	emotional > control	passive-viewing	passive-viewing	anger	scrambled images	from publication	matched
Sprengelmeyer et al., 1998	6	TAL	emotional > neutral	gender-discrimination	gender-discrimination	disgust	neutral faces	from publication	matched
	6	TAL	emotional > neutral	gender-discrimination	gender-discrimination	fear	neutral faces	from publication	matched
	6	TAL	emotional > neutral	gender-discrimination	gender-discrimination	anger	neutral faces	from publication	matched
Stevens et al., 2014	20	MNI	emotional > neutral	passive-viewing	passive-viewing	fear	neutral faces	from publication	matched
Surguladze et al., 2003	9	TAL	emotional > neutral	gender-discrimination	gender-discrimination	disgust	neutral faces	from publication	matched
	9	TAL	emotional > neutral	gender-discrimination	gender-discrimination	happiness	neutral faces	from publication	matched
	9	TAL	emotional > neutral	gender-discrimination	gender-discrimination	sadness	neutral faces	from publication	matched
	9	TAL	emotional > neutral	gender-discrimination	gender-discrimination	fear	neutral faces	from publication	matched
Surguladze et al., 2008	29	TAL	neutral > baseline	gender-discrimination	-	neutral	fixation cross	from publication	matched
	29	TAL	emotional > baseline	gender-discrimination	-	fear	fixation cross	from publication	matched
Surguladze et al., 2010	9	TAL	emotional > neutral	gender-discrimination	gender-discrimination	fear	neutral faces	from publication	
	9	TAL	emotional > neutral	gender-discrimination	gender-discrimination	disgust	neutral faces	from publication	
Surguladze et al., 2011	16	TAL	emotional > baseline	gender-discrimination	-	fear	fixation cross	from publication	matched
	16	TAL	emotional > baseline	gender-discrimination	-	happiness	fixation cross	from publication	matched

Studies	N	Space	Contrast	Task (condition of interest)	Task (contrasting condition)	Stimuli (condition of interest)	Stimuli (contrasting condition)	Source	Sub-analysis
	16	TAL	neutral > baseline	gender-discrimination	-	neutral	fixation cross	from publication	matched
Szabó et al., 2018	49	MNI	emotional > baseline	gender-discrimination	-	fear, happiness, sadness	rest	sent by authors	matched
	49	MNI	emotional > neutral	gender-discrimination	gender-discrimination	fear	neutral faces	sent by authors	matched
	49	MNI	emotional > neutral	gender-discrimination	gender-discrimination	happiness	neutral faces	sent by authors	matched
	49	MNI	emotional > neutral	gender-discrimination	gender-discrimination	sadness	neutral faces	sent by authors	matched
	49	MNI	emotional > neutral	gender-discrimination	gender-discrimination	fear, happiness, sadness	neutral faces	sent by authors	matched
	49	MNI	neutral > baseline	gender-discrimination	-	neutral	rest	sent by authors	matched
Szaflarski et al., 2018	24	MNI	emotional > baseline	gender-discrimination	-	sadness	implicit	from publication	matched
	24	MNI	emotional > baseline	gender-discrimination	-	fear	implicit	from publication	matched
	24	MNI	emotional > baseline	gender-discrimination	-	happiness	implicit	from publication	matched
	24	MNI	neutral > baseline	gender-discrimination	-	neutral	implicit	from publication	matched
Todorov et al., 2008	15	TAL	neutral > baseline	identity-matching	-	neutral	fixation cross	from publication	matched
Trautmann et al., 2009	16	TAL	emotional > neutral	passive-viewing	passive-viewing	disgust	neutral faces	from publication	matched
	16	TAL	emotional > neutral	passive-viewing	passive-viewing	happiness	neutral faces	from publication	matched
Via et al., 2014	67	MNI	emotional > control	emotion-matching	shape-matching	fear	shapes	from publication	
Villalta-Gil et al., 2017	32	MNI	neutral > baseline	identity-matching	-	neutral	implicit	from publication	matched
	32	MNI	emotional > control	identity-matching	identity-matching	anger, fear, surprise	shapes (circles, ellipses)	from publication	matched
	32	MNI	emotional > baseline	identity-matching	-	anger, fear, surprise	implicit	from publication	matched
Vuilleumier et al., 2001	12	MNI	emotional > neutral	identity-matching	identity-matching	fear	neutral faces	from publication	matched

Studies	N	Space	Contrast	Task (condition of interest)	Task (contrasting condition)	Stimuli (condition of interest)	Stimuli (contrasting condition)	Source	Sub-analysis
Vuilleumier et al., 2004	13	MNI	emotional > neutral	identity-matching	identity-matching	fear	neutral faces	from publication	
Weisenbach et al., 2014 - group 1	21	MNI	emotional > neutral	emotion-discrimination	emotion-discrimination	anger	neutral faces	from publication	matched
	21	MNI	emotional > neutral	emotion-discrimination	emotion-discrimination	fear	neutral faces	from publication	matched
	21	MNI	emotional > neutral	emotion-discrimination	emotion-discrimination	sadness	neutral faces	from publication	matched
	21	MNI	emotional > neutral	emotion-discrimination	emotion-discrimination	happiness	neutral faces	from publication	matched
Weisenbach et al., 2014 - group 2	17	MNI	emotional > neutral	emotion-discrimination	emotion-discrimination	sadness	neutral faces	from publication	
	17	MNI	emotional > neutral	emotion-discrimination	emotion-discrimination	anger	neutral faces	from publication	
	17	MNI	emotional > neutral	emotion-discrimination	emotion-discrimination	fear	neutral faces	from publication	
	17	MNI	emotional > neutral	emotion-discrimination	emotion-discrimination	happiness	neutral faces	from publication	
Williams et al., 2001	11	TAL	emotional > neutral	gender-discrimination	gender-discrimination	fear	neutral faces	from publication	
Williams et al., 2004a, 2004b	22	TAL	emotional > neutral	gender-discrimination	gender-discrimination	fear	neutral faces	from publication	matched
Williams et al., 2005	13	TAL	emotional > neutral	gender-discrimination	gender-discrimination	fear	neutral faces	from publication	
	13	TAL	emotional > neutral	gender-discrimination	gender-discrimination	anger	neutral faces	from publication	
	13	TAL	emotional > neutral	gender-discrimination	gender-discrimination	disgust	neutral faces	from publication	
Williams et al., 2006	13	MNI	emotional > neutral	passive-viewing	passive-viewing	fear	neutral faces	from publication	matched
Wright et al., 2006	12	TAL	neutral > control	identity-matching	pattern-matching	neutral	scrambled images	from publication	matched
	12	TAL	emotional > control	emotion-matching	pattern-matching	anger, fear, happiness	scrambled images	from publication	matched
Zsoldos et al., 2016 - group 1	17	MNI	emotional > neutral	gender-discrimination	gender-discrimination	fear	neutral faces	from publication	

Studies	N	Space	Contrast	Task (condition of interest)	Task (contrasting condition)	Stimuli (condition of interest)	Stimuli (contrasting condition)	Source	Sub-analysis
Zsoldos et al., 2016 - group 2	17	MNI	emotional > neutral	gender-discrimination	gender-discrimination	fear	neutral faces	from publication	

4 References

Adleman, N. E., Menon, V., Blasey, C. M., White, C. D., Warsofsky, I. S., Glover, G. H., & Reiss, A. L. (2002). A Developmental fMRI Study of the Stroop Color-Word Task. *NeuroImage*, *16*(1), 61–75. https://doi.org/10.1006/nimg.2001.1046

Agostini, A., Ballotta, D., Righi, S., Moretti, M., Bertani, A., Scarcelli, A., Sartini, A., Ercolani, M., Nichelli, P., Campieri, M., & Benuzzi, F. (2017). Stress and brain functional changes in patients with Crohn's disease: A functional magnetic resonance imaging study. *Neurogastroenterology & Motility*, *29*(10), e13108. https://doi.org/10.1111/nmo.13108

Aguilar-Ortiz, S., Salgado-Pineda, P., Vega, D., Pascual, J. C., Marco-Pallarés, J., Soler, J., Brunel, C., Martin-Blanco, A., Soto, A., Ribas, J., Maristany, T., Sarró, S., Rodríguez-Fornells, A., Salvador, R., McKenna, P. J., & Pomarol-Clotet, E. (2020). Evidence for default mode network dysfunction in borderline personality disorder. *Psychological Medicine*, *50*(10), 1746–1754. https://doi.org/10.1017/S0033291719001880

Aguirre, N., Cruz-Gómez, Á. J., Miró-Padilla, A., Bueichekú, E., Broseta Torres, R., Ávila, C., Sanchis-Segura, C., & Forn, C. (2019). Repeated Working Memory Training Improves Task Performance and Neural Efficiency in Multiple Sclerosis Patients and Healthy Controls. *Multiple Sclerosis International*, *2019*, 1–13. https://doi.org/10.1155/2019/2657902

Alain, C., Khatamian, Y., He, Y., Lee, Y., Moreno, S., Leung, A. W. S., & Bialystok, E. (2018). Different neural activities support auditory working memory in musicians and

bilinguals: Neural resources in musicians and bilinguals. *Annals of the New York Academy of Sciences*, *1423*(1), 435–446. https://doi.org/10.1111/nyas.13717

Alain, C., Shen, D., Yu, H., & Grady, C. (2010). Dissociable Memory- and Response-Related Activity in Parietal Cortex During Auditory Spatial Working Memory. *Frontiers in Psychology*, *1*. https://doi.org/10.3389/fpsyg.2010.00202

Allen, P. P., Cleare, A. J., Lee, F., Fusar-Poli, P., Tunstall, N., Fu, C. H. Y., Brammer, M. J., & McGuire, P. K. (2006). Effect of acute tryptophan depletion on pre-frontal engagement. *Psychopharmacology*, *187*(4), 486–497. https://doi.org/10.1007/s00213-006-0444-x

Alonso-Lana, S., Goikolea, J. M., Bonnin, C. M., Sarró, S., Segura, B., Amann, B. L., Monté, G. C., Moro, N., Fernandez-Corcuera, P., Maristany, T., Salvador, R., Vieta, E., Pomarol-Clotet, E., & McKenna, P. J. (2016). Structural and Functional Brain Correlates of Cognitive Impairment in Euthymic Patients with Bipolar Disorder. *PLOS ONE*, *11*(7), e0158867. https://doi.org/10.1371/journal.pone.0158867

Amann, M., Dössegger, L. S., Penner, I.-K., Hirsch, J. G., Raselli, C., Calabrese, P., Weier, K., Radü, E.-W., Kappos, L., & Gass, A. (2011). Altered functional adaptation to attention and working memory tasks with increasing complexity in relapsing-remitting multiple sclerosis patients: Functional Adaptation in MS Patients. *Human Brain Mapping*, *32*(10), 1704–1719. https://doi.org/10.1002/hbm.21142

Awh, E., Jonides, J., Smith, E. E., Schumacher, E. H., Koeppe, R. A., & Katz, S. (1996). Dissociation of Storage and Rehearsal in Verbal Working Memory: Evidence From Positron Emission Tomography. *Psychological Science*, *7*(1), 25–31. https://doi.org/10.1111/j.1467-9280.1996.tb00662.x

Banich, M. T., Milham, M. P., Jacobson, B. L., Webb, A., Wszalek, T., Cohen, N. J., & Kramer, A. F. (2001). Chapter 29 Attentional selection and the processing of task-irrelevant information: Insights from fMRI examinations of the Stroop task. In *Progress*

in Brain Research (Vol. 134, pp. 459–470). Elsevier. https://doi.org/10.1016/S0079-6123(01)34030-X

Barch, D. M., Burgess, G. C., Harms, M. P., Petersen, S. E., Schlaggar, B. L., Corbetta, M., Glasser, M. F., Curtiss, S., Dixit, S., Feldt, C., Nolan, D., Bryant, E., Hartley, T., Footer, O., Bjork, J. M., Poldrack, R., Smith, S., Johansen-Berg, H., Snyder, A. Z., & Van Essen, D. C. (2013). Function in the human connectome: Task-fMRI and individual differences in behavior. *NeuroImage*, *80*, 169–189. https://doi.org/10.1016/j.neuroimage.2013.05.033

Barkley-Levenson, E., Xue, F., Droutman, V., Miller, L. C., Smith, B. J., Jeong, D., Lu, Z.-L., Bechara, A., & Read, S. J. (2018). Prefrontal Cortical Activity During the Stroop Task: New Insights into the Why and the Who of Real-World Risky Sexual Behavior. *Annals of Behavioral Medicine*, *52*(5), 367–379. https://doi.org/10.1093/abm/kax019

Bas-Hoogendam, J. M., Andela, C. D., van der Werff, S. J. A., Pannekoek, J. N., van Steenbergen, H., Meijer, O. C., van Buchem, M. A., Rombouts, S. A. R. B., van der Mast, R. C., Biermasz, N. R., van der Wee, N. J. A., & Pereira, A. M. (2015). Altered neural processing of emotional faces in remitted Cushing's disease. *Psychoneuroendocrinology*, *59*, 134–146. https://doi.org/10.1016/j.psyneuen.2015.05.001

Basten, U., Stelzel, C., & Fiebach, C. J. (2011). Trait Anxiety Modulates the Neural Efficiency of Inhibitory Control. *Journal of Cognitive Neuroscience*, *23*(10), 3132–3145. https://doi.org/10.1162/jocn_a_00003

Batut, A.-C., Gounot, D., Namer, I. J., Hirsch, E., Kehrli, P., & Metz-Lutz, M.-N. (2006). Neural responses associated with positive and negative emotion processing in patients with left versus right temporal lobe epilepsy. *Epilepsy & Behavior*, *9*(3), 415–423. https://doi.org/10.1016/j.yebeh.2006.07.013

Becker, T. M., Kerns, J. G., MacDonald, A. W., & Carter, C. S. (2008). Prefrontal Dysfunction in First-Degree Relatives of Schizophrenia Patients during a Stroop Task. *Neuropsychopharmacology*, *33*(11), 2619–2625. https://doi.org/10.1038/sj.npp.1301673

Bench, C. J., Frith, C. D., Grasby, P. M., Friston, K. J., Paulesu, E., Frackowiak, R. S. J., & Dolan, R. J. (1993). Investigations of the functional anatomy of attention using the stroop test. *Neuropsychologia*, *31*(9), 907–922. https://doi.org/10.1016/0028-3932(93)90147-R

Benuzzi, F., Meletti, S., Zamboni, G., Calandra-Buonaura, G., Serafini, M., Lui, F., Baraldi, P., Rubboli, G., Tassinari, C. A., & Nichelli, P. (2004). Impaired fear processing in right mesial temporal sclerosis: A fMRI study. *Brain Research Bulletin*, *63*(4), 269–281. https://doi.org/10.1016/j.brainresbull.2004.03.005

Benuzzi, F., Pugnaghi, M., Meletti, S., Lui, F., Serafini, M., Baraldi, P., & Nichelli, P. (2007). Processing the socially relevant parts of faces. *Brain Research Bulletin*, *74*(5), 344–356. https://doi.org/10.1016/j.brainresbull.2007.07.010

Binelli, C., Muñiz, A., Subira, S., Navines, R., Blanco-Hinojo, L., Perez-Garcia, D., Crippa, J., Farré, M., Pérez-Jurado, L., Pujol, J., & Martin-Santos, R. (2016). Facial emotion processing in patients with social anxiety disorder and Williams–Beuren syndrome: An fMRI study. *Journal of Psychiatry and Neuroscience*, *41*(3), 182–191. https://doi.org/10.1503/jpn.140384

Bird, G., Catmur, C., Silani, G., Frith, C., & Frith, U. (2006). Attention does not modulate neural responses to social stimuli in autism spectrum disorders. *NeuroImage*, *31*(4), 1614–1624. https://doi.org/10.1016/j.neuroimage.2006.02.037

Bleich-Cohen, M., Hendler, T., Weizman, R., Faragian, S., Weizman, A., & Poyurovsky, M. (2014). Working memory dysfunction in schizophrenia patients with obsessive-compulsive symptoms: An fMRI study. *European Psychiatry*, *29*(3), 160–166. https://doi.org/10.1016/j.eurpsy.2013.02.004

Boller, B., Mellah, S., Ducharme-Laliberté, G., & Belleville, S. (2017). Relationships between years of education, regional grey matter volumes, and working memory-related brain activity in healthy older adults. *Brain Imaging and Behavior*, *11*(2), 304–317. https://doi.org/10.1007/s11682-016-9621-7

Borgsted, C., Ozenne, B., Mc Mahon, B., Madsen, M. K., Hjordt, L. V., Hageman, I., Baaré, W. F. C., Knudsen, G. M., & Fisher, P. M. (2018). Amygdala response to emotional faces in seasonal affective disorder. *Journal of Affective Disorders*, *229*, 288–295. https://doi.org/10.1016/j.jad.2017.12.097

Brass, M., Derrfuss, J., & von Cramon, D. Y. (2005). The inhibition of imitative and overlearned responses: A functional double dissociation. *Neuropsychologia*, *43*(1), 89–98. https://doi.org/10.1016/j.neuropsychologia.2004.06.018

Briceño, E. M., Weisenbach, S. L., Rapport, L. J., Hazlett, K. E., Bieliauskas, L. A., Haase, B. D., Ransom, M. T., Brinkman, M. L., Peciña, M., Schteingart, D. E., Starkman, M. N., Giordani, B., Welsh, R. C., Noll, D. C., Zubieta, J.-K., & Langenecker, S. A. (2013). Shifted inferior frontal laterality in women with major depressive disorder is related to emotion-processing deficits. *Psychological Medicine*, *43*(7), 1433–1445. https://doi.org/10.1017/S0033291712002176

Brown, S. M., Manuck, S. B., Flory, J. D., & Hariri, A. R. (2006). Neural basis of individual differences in impulsivity: Contributions of corticolimbic circuits for behavioral arousal and control. *Emotion*, *6*(2), 239–245. https://doi.org/10.1037/1528-3542.6.2.239

Bukowski, H., Dricot, L., Hanseeuw, B., & Rossion, B. (2013). Cerebral lateralization of face-sensitive areas in left-handers: Only the FFA does not get it right. *Cortex*, *49*(9), 2583–2589. https://doi.org/10.1016/j.cortex.2013.05.002

Campanella, S., Peigneux, P., Petit, G., Lallemand, F., Saeremans, M., Noël, X., Metens, T., Nouali, M., De Tiège, X., De Witte, P., Ward, R., & Verbanck, P. (2013). Increased Cortical Activity in Binge Drinkers during Working Memory Task: A

Preliminary Assessment through a Functional Magnetic Resonance Imaging Study. *PLoS ONE*, *8*(4), e62260. https://doi.org/10.1371/journal.pone.0062260

Cardoner, N., Harrison, B. J., Pujol, J., Soriano-Mas, C., Hernández-Ribas, R., López-Solá, M., Real, E., Deus, J., Ortiz, H., Alonso, P., & Menchón, J. M. (2011). Enhanced brain responsiveness during active emotional face processing in obsessive compulsive disorder. *The World Journal of Biological Psychiatry*, *12*(5), 349–363. https://doi.org/10.3109/15622975.2011.559268

Carter, C. S., Mintun, M., & Cohen, J. D. (1995). Interference and Facilitation Effects during Selective Attention: An H215O PET Study of Stroop Task Performance. *NeuroImage*, *2*(4), 264–272. https://doi.org/10.1006/nimg.1995.1034

Carvajal, F., Rubio, S., Serrano, J. M., Ríos-Lago, M., Alvarez-Linera, J., Pacheco, L., & Martín, P. (2013). Is a neutral expression also a neutral stimulus? A study with functional magnetic resonance. *Experimental Brain Research*, *228*(4), 467–479. https://doi.org/10.1007/s00221-013-3578-1

Cerasa, A., Gioia, M. C., Fera, F., Passamonti, L., Liguori, M., Lanza, P., Muglia, M., Magariello, A., & Quattrone, A. (2008). Ventro-lateral prefrontal activity during working memory is modulated by MAO A genetic variation. *Brain Research*, *1201*, 114–121. https://doi.org/10.1016/j.brainres.2008.01.048

Chen, Z., Zhao, X., Fan, J., & Chen, A. (2018). Functional cerebral asymmetry analyses reveal how the control system implements its flexibility. *Human Brain Mapping*, *39*(12), 4678–4688. https://doi.org/10.1002/hbm.24313

Choo, W.-C., Lee, W.-W., Venkatraman, V., Sheu, F.-S., & Chee, M. W. L. (2005). Dissociation of cortical regions modulated by both working memory load and sleep deprivation and by sleep deprivation alone. *NeuroImage*, *25*(2), 579–587. https://doi.org/10.1016/j.neuroimage.2004.11.029

Ciesielski, K. T., Lesnik, P. G., Savoy, R. L., Grant, E. P., & Ahlfors, S. P. (2006). Developmental neural networks in children performing a Categorical N-Back Task. *NeuroImage*, *33*(3), 980–990. https://doi.org/10.1016/j.neuroimage.2006.07.028

Cieslik, E. C., Mueller, V. I., Eickhoff, C. R., Langner, R., & Eickhoff, S. B. (2015). Three key regions for supervisory attentional control: Evidence from neuroimaging meta-analyses. *Neuroscience & Biobehavioral Reviews*, *48*, 22–34. https://doi.org/10.1016/j.neubiorev.2014.11.003

Clark, C. M., Lawlor-Savage, L., & Goghari, V. M. (2017). Comparing brain activations associated with working memory and fluid intelligence. *Intelligence*, *63*, 66–77. https://doi.org/10.1016/j.intell.2017.06.001

Coderre, E. L., Filippi, C. G., Newhouse, P. A., & Dumas, J. A. (2008). The Stroop effect in kana and kanji scripts in native Japanese speakers: An fMRI study. *Brain and Language*, *107*(2), 124–132. https://doi.org/10.1016/j.bandl.2008.01.011

Coderre, E. L., & van Heuven, W. J. B. (2013). Modulations of the executive control network by stimulus onset asynchrony in a Stroop task. *BMC Neuroscience*, *14*(1), 79. https://doi.org/10.1186/1471-2202-14-79

Cohen, J. (2013). *Statistical Power Analysis for the Behavioral Sciences.* Elsevier Science.

Contreras-Rodríguez, O., Pujol, J., Batalla, I., Harrison, B. J., Bosque, J., Ibern-Regàs, I., Hernández-Ribas, R., Soriano-Mas, C., Deus, J., López-Solà, M., Pifarré, J., Menchón, J. M., & Cardoner, N. (2014). Disrupted neural processing of emotional faces in psychopathy. *Social Cognitive and Affective Neuroscience*, *9*(4), 505–512. https://doi.org/10.1093/scan/nst014

Critchley, H. D., Rotshtein, P., Nagai, Y., O'Doherty, J., Mathias, C. J., & Dolan, R. J. (2005). Activity in the human brain predicting differential heart rate responses to emotional facial expressions. *NeuroImage*, *24*(3), 751–762. https://doi.org/10.1016/j.neuroimage.2004.10.013

Cullen, K. R., LaRiviere, L. L., Vizueta, N., Thomas, K. M., Hunt, R. H., Miller, M. J., Lim, K. O., & Schulz, S. C. (2016). Brain activation in response to overt and covert fear and happy faces in women with borderline personality disorder. *Brain Imaging and Behavior*, *10*(2), 319–331. https://doi.org/10.1007/s11682-015-9406-4

Daamen, M., Bäuml, J. G., Scheef, L., Sorg, C., Busch, B., Baumann, N., Bartmann, P., Wolke, D., Wohlschläger, A., & Boecker, H. (2015). Working memory in preterm-born adults: Load-dependent compensatory activity of the posterior default mode network: Working Memory in Preterm-Born Adults. *Human Brain Mapping*, *36*(3), 1121–1137. https://doi.org/10.1002/hbm.22691

Dan, R., Růžička, F., Bezdicek, O., Roth, J., Růžička, E., Vymazal, J., Goelman, G., & Jech, R. (2019). Impact of dopamine and cognitive impairment on neural reactivity to facial emotion in Parkinson's disease. *European Neuropsychopharmacology*, *29*(11), 1258–1272. https://doi.org/10.1016/j.euroneuro.2019.09.003

Deckersbach, T., Rauch, S. L., Buhlmann, U., Ostacher, M. J., Beucke, J.-C., Nierenberg, A. A., Sachs, G., & Dougherty, D. D. (2008). An fMRI investigation of working memory and sadness in females with bipolar disorder: A brief report. *Bipolar Disorders*, *10*(8), 928–942. https://doi.org/10.1111/j.1399-5618.2008.00633.x

Deeley, Q., Daly, E. M., Surguladze, S., Page, L., Toal, F., Robertson, D., Curran, S., Giampietro, V., Seal, M., Brammer, M. J., Andrew, C., Murphy, K., Phillips, M. L., & Murphy, D. G. M. (2007). An Event Related Functional Magnetic Resonance Imaging Study of Facial Emotion Processing in Asperger Syndrome. *Biological Psychiatry*, *62*(3), 207–217. https://doi.org/10.1016/j.biopsych.2006.09.037

Demenescu, L. R., Renken, R., Kortekaas, R., van Tol, M.-J., Marsman, J. B. C., van Buchem, M. A., van der Wee, N. J. A., Veltman, D. J., den Boer, J. A., & Aleman, A. (2011). Neural correlates of perception of emotional facial expressions in out-patients with mild-to-moderate depression and anxiety. A multicenter fMRI study. *Psychological Medicine*, *41*(11), 2253–2264. https://doi.org/10.1017/S0033291711000596

DeVito, E. E., Worhunsky, P. D., Carroll, K. M., Rounsaville, B. J., Kober, H., & Potenza, M. N. (2012). A preliminary study of the neural effects of behavioral therapy for substance use disorders. *Drug and Alcohol Dependence*, *122*(3), 228–235. https://doi.org/10.1016/j.drugalcdep.2011.10.002

Dima, D., Jogia, J., & Frangou, S. (2014). Dynamic causal modeling of load-dependent modulation of effective connectivity within the verbal working memory network: Brain Connectivity in Increasing Memory Load. *Human Brain Mapping*, *35*(7), 3025–3035. https://doi.org/10.1002/hbm.22382

Dima, D., Stephan, K. E., Roiser, J. P., Friston, K. J., & Frangou, S. (2011). Effective Connectivity during Processing of Facial Affect: Evidence for Multiple Parallel Pathways. *Journal of Neuroscience*, *31*(40), 14378–14385. https://doi.org/10.1523/JNEUROSCI.2400-11.2011

Döhnel, K., Sommer, M., Ibach, B., Rothmayr, C., Meinhardt, J., & Hajak, G. (2008). Neural correlates of emotional working memory in patients with mild cognitive impairment. *Neuropsychologia*, *46*(1), 37–48. https://doi.org/10.1016/j.neuropsychologia.2007.08.012

Dolan, R. J., Fletcher, P., Morris, J., Kapur, N., Deakin, J. F. W., & Frith, C. D. (1996). Neural Activation during Covert Processing of Positive Emotional Facial Expressions. *NeuroImage*, *4*(3), 194–200. https://doi.org/10.1006/nimg.1996.0070

Domínguez-Borràs, J., Rieger, S. W., Corradi-Dell'Acqua, C., Neveu, R., & Vuilleumier, P. (2017). Fear Spreading Across Senses: Visual Emotional Events Alter Cortical Responses to Touch, Audition, and Vision. *Cerebral Cortex*, *27*(1), 68–82. https://doi.org/10.1093/cercor/bhw337

Dores, A. R., Barbosa, F., Carvalho, I. P., Almeida, I., Guerreiro, S., da Rocha, B. M., de Sousa, L., & Castro-Caldas, A. (2017). Study of behavioural and neural bases of visuo-spatial working memory with an fMRI paradigm based on an n-back task. *Journal of Neuropsychology*, *11*(1), 122–134. https://doi.org/10.1111/jnp.12076

Drapier, D., Surguladze, S., Marshall, N., Schulze, K., Fern, A., Hall, M.-H., Walshe, M., Murray, R. M., & McDonald, C. (2008). Genetic Liability for Bipolar Disorder Is Characterized by Excess Frontal Activation in Response to a Working Memory Task. *Biological Psychiatry*, *64*(6), 513–520. https://doi.org/10.1016/j.biopsych.2008.04.038

Duggirala, S. X., Saharan, S., Raghunathan, P., & Mandal, P. K. (2016). Stimulus-dependent modulation of working memory for identity monitoring: A functional MRI study. *Brain and Cognition*, *102*, 55–64. https://doi.org/10.1016/j.bandc.2015.12.006

Eickhoff, S. B., Nichols, T. E., Laird, A. R., Hoffstaedter, F., Amunts, K., Fox, P. T., Bzdok, D., & Eickhoff, C. R. (2016). Behavior, sensitivity, and power of activation likelihood estimation characterized by massive empirical simulation. *NeuroImage*, *137*, 70–85. https://doi.org/10.1016/j.neuroimage.2016.04.072

Engell, A. D., & Haxby, J. V. (2007). Facial expression and gaze-direction in human superior temporal sulcus. *Neuropsychologia*, *45*(14), 3234–3241. https://doi.org/10.1016/j.neuropsychologia.2007.06.022

Esteban, O., Ciric, R., Finc, K., Blair, R. W., Markiewicz, C. J., Moodie, C. A., Kent, J. D., Goncalves, M., DuPre, E., Gomez, D. E. P., Ye, Z., Salo, T., Valabregue, R., Amlien, I. K., Liem, F., Jacoby, N., Stojić, H., Cieslak, M., Urchs, S., … Gorgolewski, K. J. (2020). Analysis of task-based functional MRI data preprocessed with fMRIPrep. *Nature Protocols*, *15*(7), 2186–2202. https://doi.org/10.1038/s41596-020-0327-3

Esteves, M., Magalhães, R., Marques, P., Castanho, T. C., Portugal-Nunes, C., Soares, J. M., Almeida, A., Santos, N. C., Sousa, N., & Leite-Almeida, H. (2018). Functional Hemispheric (A)symmetries in the Aged Brain—Relevance for Working Memory. *Frontiers in Aging Neuroscience*, *10*, 58. https://doi.org/10.3389/fnagi.2018.00058

Ethofer, T., Bretscher, J., Wiethoff, S., Bisch, J., Schlipf, S., Wildgruber, D., & Kreifelts, B. (2013). Functional responses and structural connections of cortical areas for processing faces and voices in the superior temporal sulcus. *NeuroImage*, *76*, 45–56. https://doi.org/10.1016/j.neuroimage.2013.02.064

Ewbank, M., Fox, E., & Calder, A. (2010). The interaction between gaze and facial expression in the amygdala and extended amygdala is modulated by anxiety. *Frontiers in Human Neuroscience*, *4*. https://doi.org/10.3389/fnhum.2010.00056

Fakra, E., Salgado-Pineda, P., Delaveau, P., Hariri, A. R., & Blin, O. (2008). Neural bases of different cognitive strategies for facial affect processing in schizophrenia. *Schizophrenia Research*, *100*(1-3), 191–205. https://doi.org/10.1016/j.schres.2007.11.040

Fan, J., Flombaum, J. I., McCandliss, B. D., Thomas, K. M., & Posner, M. I. (2003). Cognitive and Brain Consequences of Conflict. *NeuroImage*, *18*(1), 42–57. https://doi.org/10.1006/nimg.2002.1319

Fechir, M., Gamer, M., Blasius, I., Bauermann, T., Breimhorst, M., Schlindwein, P., Schlereth, T., & Birklein, F. (2010). Functional imaging of sympathetic activation during mental stress. *NeuroImage*, *50*(2), 847–854. https://doi.org/10.1016/j.neuroimage.2009.12.004

Fernández-Corcuera, P., Salvador, R., Monté, G. C., Salvador Sarró, S., Goikolea, J. M., Amann, B., Moro, N., Sans-Sansa, B., Ortiz-Gil, J., Vieta, E., Maristany, T., McKenna, P. J., & Pomarol-Clotet, E. (2013). Bipolar depressed patients show both failure to activate and failure to de-activate during performance of a working memory task. *Journal of Affective Disorders*, *148*(2-3), 170–178. https://doi.org/10.1016/j.jad.2012.04.009

Fitzgerald, D. A., Angstadt, M., Jelsone, L. M., Nathan, P. J., & Phan, K. L. (2006). Beyond threat: Amygdala reactivity across multiple expressions of facial affect. *NeuroImage*, *30*(4), 1441–1448. https://doi.org/10.1016/j.neuroimage.2005.11.003

Forn, C., Barros-Loscertales, A., Escudero, J., Benlloch, V., Campos, S., Antònia Parcet, M., & Ávila, C. (2007). Compensatory activations in patients with multiple sclerosis during preserved performance on the auditory N-back task. *Human Brain Mapping*, *28*(5), 424–430. https://doi.org/10.1002/hbm.20284

Frässle, S., Paulus, F. M., Krach, S., Schweinberger, S. R., Stephan, K. E., & Jansen, A. (2016). Mechanisms of hemispheric lateralization: Asymmetric interhemispheric recruitment in the face perception network. *NeuroImage*, *124*, 977–988. https://doi.org/10.1016/j.neuroimage.2015.09.055

Fuentes-Claramonte, P., López-Araquistain, L., Sarró, S., Sans-Sansa, B., Ortiz-Gil, J., Maristany, T., Salvador, R., McKenna, P. J., & Pomarol-Clotet, E. (2021). Brain functional correlates of formal thought disorder in schizophrenia: Examining the frontal/dysexecutive hypothesis. *Psychological Medicine*, *51*(14), 2446–2453. https://doi.org/10.1017/S0033291720001063

Fuentes-Claramonte, P., Martín-Subero, M., Salgado-Pineda, P., Alonso-Lana, S., Moreno-Alcázar, A., Argila-Plaza, I., Santo-Angles, A., Albajes-Eizagirre, A., Anguera-Camós, M., Capdevila, A., Sarró, S., McKenna, P. J., Pomarol-Clotet, E., & Salvador, R. (2019). Shared and differential default-mode related patterns of activity in an autobiographical, a self-referential and an attentional task. *PLOS ONE*, *14*(1), e0209376. https://doi.org/10.1371/journal.pone.0209376

Fukuda, Y., Katthagen, T., Deserno, L., Shayegan, L., Kaminski, J., Heinz, A., & Schlagenhauf, F. (2019). Reduced parietofrontal effective connectivity during a working-memory task in people with high delusional ideation. *Journal of Psychiatry & Neuroscience*, *44*(3), 195–204. https://doi.org/10.1503/jpn.180043

Garrett, A., Kelly, R., Gomez, R., Keller, J., Schatzberg, A. F., & Reiss, A. L. (2011). Aberrant Brain Activation During a Working Memory Task in Psychotic Major Depression. *American Journal of Psychiatry*, *168*(2), 173–182. https://doi.org/10.1176/appi.ajp.2010.09121718

George, M. S., Ketter, T. A., Parekh, P. I., Rosinsky, N., Ring, H., Casey, B. J., Trimble, M. R., Horwitz, B., Herscovitch, P., & Post, R. M. (1994). Regional brain activity when selecting a response despite interference: An $H_2{}^{15}O$ PET study of the stroop and an emotional stroop: The Stroop. *Human Brain Mapping*, *1*(3), 194–209. https://doi.org/10.1002/hbm.460010305

Germine, L. T., Garrido, L., Bruce, L., & Hooker, C. (2011). Social anhedonia is associated with neural abnormalities during face emotion processing. *NeuroImage*, *58*(3), 935–945. https://doi.org/10.1016/j.neuroimage.2011.06.059

Ghavidel, N., Khodagholi, F., Ahmadiani, A., Khosrowabadi, R., Asadi, S., & Shams, J. (2020). Frontocingulate Dysfunction Is Associated with Depression and Decreased Serum PON1 in Methamphetamine-Dependent Patients. *Neuropsychiatric Disease and Treatment*, *Volume 16*, 489–499. https://doi.org/10.2147/NDT.S237528

Gianaros, P. J., Hariri, A. R., Sheu, L. K., Muldoon, M. F., Sutton-Tyrrell, K., & Manuck, S. B. (2009). Preclinical Atherosclerosis Covaries with Individual Differences in Reactivity and Functional Connectivity of the Amygdala. *Biological Psychiatry*, *65*(11), 943–950. https://doi.org/10.1016/j.biopsych.2008.10.007

Gianaros, P. J., Kraynak, T. E., Kuan, D. C.-H., Gross, J. J., McRae, K., Hariri, A. R., Manuck, S. B., Rasero, J., & Verstynen, T. D. (2020). Affective brain patterns as multivariate neural correlates of cardiovascular disease risk. *Social Cognitive and Affective Neuroscience*, *15*(10), 1034–1045. https://doi.org/10.1093/scan/nsaa050

Gianaros, P. J., Sheu, L. K., Matthews, K. A., Jennings, J. R., Manuck, S. B., & Hariri, A. R. (2008). Individual Differences in Stressor-Evoked Blood Pressure Reactivity Vary with Activation, Volume, and Functional Connectivity of the Amygdala. *Journal of Neuroscience*, *28*(4), 990–999. https://doi.org/10.1523/JNEUROSCI.3606-07.2008

Gillis, M. M., Garcia, S., & Hampstead, B. M. (2016). Working memory contributes to the encoding of object location associations: Support for a 3-part model of object location memory. *Behavioural Brain Research*, *311*, 192–200. https://doi.org/10.1016/j.bbr.2016.05.037

Glasser, M. F., Sotiropoulos, S. N., Wilson, J. A., Coalson, T. S., Fischl, B., Andersson, J. L., Xu, J., Jbabdi, S., Webster, M., Polimeni, J. R., Van Essen, D. C., & Jenkinson, M. (2013). The minimal preprocessing pipelines for the Human Connectome Project. *NeuroImage*, *80*, 105–124. https://doi.org/10.1016/j.neuroimage.2013.04.127

Goikolea, J. M., Dima, D., Landín-Romero, R., Torres, I., DelVecchio, G., Valentí, M., Amann, B. L., Bonnín, C. M., McKenna, P. J., Pomarol-Clotet, E., Frangou, S., & Vieta, E. (2019). Multimodal Brain Changes in First-Episode Mania: A Voxel-Based Morphometry, Functional Magnetic Resonance Imaging, and Connectivity Study. *Schizophrenia Bulletin*, *45*(2), 464–473. https://doi.org/10.1093/schbul/sby047

Gorgolewski, K. J., Varoquaux, G., Rivera, G., Schwartz, Y., Sochat, V. V., Ghosh, S. S., Maumet, C., Nichols, T. E., Poline, J.-B., Yarkoni, T., Margulies, D. S., & Poldrack, R. A. (2016). NeuroVault.org: A repository for sharing unthresholded statistical maps, parcellations, and atlases of the human brain. *NeuroImage*, *124*, 1242–1244. https://doi.org/10.1016/j.neuroimage.2015.04.016

Grandjean, J., D'Ostilio, K., Fias, W., Phillips, C., Balteau, E., Degueldre, C., Luxen, A., Maquet, P., Salmon, E., & Collette, F. (2013). Exploration of the mechanisms underlying the ISPC effect: Evidence from behavioral and neuroimaging data. *Neuropsychologia*, *51*(6), 1040–1049. https://doi.org/10.1016/j.neuropsychologia.2013.02.015

Grandjean, J., D'Ostilio, K., Phillips, C., Balteau, E., Degueldre, C., Luxen, A., Maquet, P., Salmon, E., & Collette, F. (2012). Modulation of Brain Activity during a Stroop Inhibitory Task by the Kind of Cognitive Control Required. *PLoS ONE*, *7*(7), e41513. https://doi.org/10.1371/journal.pone.0041513

Grant, M. M., Cannistraci, C., Hollon, S. D., Gore, J., & Shelton, R. (2011). Childhood trauma history differentiates amygdala response to sad faces within MDD. *Journal of Psychiatric Research*, *45*(7), 886–895. https://doi.org/10.1016/j.jpsychires.2010.12.004

Gropman, A. L., Shattuck, K., Prust, M. J., Seltzer, R. R., Breeden, A. L., Hailu, A., Rigas, A., Hussain, R., & VanMeter, J. (2013). Altered neural activation in ornithine transcarbamylase deficiency during executive cognition: An fMRI study. *Human Brain Mapping*, *34*(4), 753–761. https://doi.org/10.1002/hbm.21470

Günther, V., Hußlack, A., Weil, A.-S., Bujanow, A., Henkelmann, J., Kersting, A., Quirin, M., Hoffmann, K.-T., Egloff, B., Lobsien, D., & Suslow, T. (2020). Individual differences

in anxiety and automatic amygdala response to fearful faces: A replication and extension of Etkin et al. (2004). *NeuroImage: Clinical*, *28*, 102441. https://doi.org/10.1016/j.nicl.2020.102441

Haas, B. W., Constable, R. T., & Canli, T. (2009). Functional magnetic resonance imaging of temporally distinct responses to emotional facial expressions. *Social Neuroscience*, *4*(2), 121–134. https://doi.org/10.1080/17470910802176326

Habel, U., Koch, K., Pauly, K., Kellermann, T., Reske, M., Backes, V., Seiferth, N. Y., Stöcker, T., Kircher, T., Amunts, K., Jon Shah, N., & Schneider, F. (2007). The influence of olfactory-induced negative emotion on verbal working memory: Individual differences in neurobehavioral findings. *Brain Research*, *1152*, 158–170. https://doi.org/10.1016/j.brainres.2007.03.048

Harada, T., Mano, Y., Komeda, H., Hechtman, L. A., Pornpattananangkul, N., Parrish, T. B., Sadato, N., Iidaka, T., & Chiao, J. Y. (2020). Cultural influences on neural systems of intergroup emotion perception: An fMRI study. *Neuropsychologia*, *137*, 107254. https://doi.org/10.1016/j.neuropsychologia.2019.107254

Harding, I. H., Corben, L. A., Storey, E., Egan, G. F., Stagnitti, M. R., Poudel, G. R., Delatycki, M. B., & Georgiou-Karistianis, N. (2016). Fronto-cerebellar dysfunction and dysconnectivity underlying cognition in friedreich ataxia: The IMAGE-FRDA study: Cognitive Networks in Friedreich Ataxia. *Human Brain Mapping*, *37*(1), 338–350. https://doi.org/10.1002/hbm.23034

Heinzel, S., Lorenz, R. C., Pelz, P., Heinz, A., Walter, H., Kathmann, N., Rapp, M. A., & Stelzel, C. (2016). Neural correlates of training and transfer effects in working memory in older adults. *NeuroImage*, *134*, 236–249. https://doi.org/10.1016/j.neuroimage.2016.03.068

Hessl, D., Rivera, S., Koldewyn, K., Cordeiro, L., Adams, J., Tassone, F., Hagerman, P. J., & Hagerman, R. J. (2007). Amygdala dysfunction in men with the fragile X premutation. *Brain*, *130*(2), 404–416. https://doi.org/10.1093/brain/awl338

Honey, G. D., Bullmore, E. T., & Sharma, T. (2000). Prolonged Reaction Time to a Verbal Working Memory Task Predicts Increased Power of Posterior Parietal Cortical Activation. *NeuroImage*, *12*(5), 495–503. https://doi.org/10.1006/nimg.2000.0624

Honey, G. D., Sharma, T., Suckling, J., Giampietro, V., Soni, W., Williams, S. C. R., & Bullmore, E. T. (2003). The functional neuroanatomy of schizophrenic subsyndromes. *Psychological Medicine*, *33*(6), 1007–1018. https://doi.org/10.1017/S0033291703007864

Hough, C. M., Luks, T. L., Lai, K., Vigil, O., Guillory, S., Nongpiur, A., Fekri, S. M., Kupferman, E., Mathalon, D. H., & Mathews, C. A. (2016). Comparison of brain activation patterns during executive function tasks in hoarding disorder and non-hoarding OCD. *Psychiatry Research: Neuroimaging*, *255*, 50–59. https://doi.org/10.1016/j.pscychresns.2016.07.007

Huang, R.-R., Jia, B.-H., Xie, L., Ma, S.-H., Yin, J.-J., Sun, Z.-B., Le, H.-B., Xu, W.-C., Huang, J.-Z., & Luo, D.-X. (2016). Spatial working memory impairment in primary onset middle-age type 2 diabetes mellitus: An ethology and BOLD-fMRI study: Memory Impairment With Middle-Age Onset T2DM. *Journal of Magnetic Resonance Imaging*, *43*(1), 75–87. https://doi.org/10.1002/jmri.24967

Huang, S., Zhu, Z., Zhang, W., Chen, Y., & Zhen, S. (2017). Trait impulsivity components correlate differently with proactive and reactive control. *PLOS ONE*, *12*(4), e0176102. https://doi.org/10.1371/journal.pone.0176102

Ihme, K., Sacher, J., Lichev, V., Rosenberg, N., Kugel, H., Rufer, M., Grabe, H.-J., Pampel, A., Lepsien, J., Kersting, A., Villringer, A., & Suslow, T. (2014). Alexithymia and the labeling of facial emotions: Response slowing and increased motor and somatosensory processing. *BMC Neuroscience*, *15*(1), 40. https://doi.org/10.1186/1471-2202-15-40

Iidaka, T., Matsumoto, A., Haneda, K., Okada, T., & Sadato, N. (2006). Hemodynamic and electrophysiological relationship involved in human face processing: Evidence from

a combined fMRI–ERP study. *Brain and Cognition*, *60*(2), 176–186. https://doi.org/10.1016/j.bandc.2005.11.004

Iidaka, T., Okada, T., Murata, T., Omori, M., Kosaka, H., Sadato, N., & Yonekura, Y. (2002). Age-related differences in the medial temporal lobe responses to emotional faces as revealed by fMRI. *Hippocampus*, *12*(3), 352–362. https://doi.org/10.1002/hipo.1113

Iidaka, T., Omori, M., Murata, T., Kosaka, H., Yonekura, Y., Okada, T., & Sadato, N. (2001). Neural Interaction of the Amygdala with the Prefrontal and Temporal Cortices in the Processing of Facial Expressions as Revealed by fMRI. *Journal of Cognitive Neuroscience*, *13*(8), 1035–1047. https://doi.org/10.1162/089892901753294338

Ishitobi, M., Kosaka, H., Omori, M., Matsumura, Y., Munesue, T., Mizukami, K., Shimoyama, T., Murata, T., Sadato, N., Okazawa, H., & Wada, Y. (2011). Differential amygdala response to lower face in patients with autistic spectrum disorders: An fMRI study. *Research in Autism Spectrum Disorders*, *5*(2), 910–919. https://doi.org/10.1016/j.rasd.2010.10.005

Jablonska, K., Piotrowska, M., Bednarek, H., Szymaszek, A., Marchewka, A., Wypych, M., & Szelag, E. (2020). Maintenance vs. Manipulation in Auditory Verbal Working Memory in the Elderly: New Insights Based on Temporal Dynamics of Information Processing in the Millisecond Time Range. *Frontiers in Aging Neuroscience*, *12*, 194. https://doi.org/10.3389/fnagi.2020.00194

Jackson, M. C., Wolf, C., Johnston, S. J., Raymond, J. E., & Linden, D. E. J. (2008). Neural Correlates of Enhanced Visual Short-Term Memory for Angry Faces: An fMRI Study. *PLoS ONE*, *3*(10), e3536. https://doi.org/10.1371/journal.pone.0003536

Jaspar, M., Genon, S., Muto, V., Meyer, C., Manard, M., Dideberg, V., Bours, V., Salmon, E., Maquet, P., & Collette, F. (2014). Modulating effect of COMT genotype on the brain regions underlying proactive control process during inhibition. *Cortex*, *50*, 148–161. https://doi.org/10.1016/j.cortex.2013.06.003

Jehna, M., Langkammer, C., Wallner-Blazek, M., Neuper, C., Loitfelder, M., Ropele, S., Fuchs, S., Khalil, M., Pluta-Fuerst, A., Fazekas, F., & Enzinger, C. (2011). Cognitively preserved MS patients demonstrate functional differences in processing neutral and emotional faces. *Brain Imaging and Behavior*, *5*(4), 241–251. https://doi.org/10.1007/s11682-011-9128-1

Jehna, M., Neuper, C., Ischebeck, A., Loitfelder, M., Ropele, S., Langkammer, C., Ebner, F., Fuchs, S., Schmidt, R., Fazekas, F., & Enzinger, C. (2011). The functional correlates of face perception and recognition of emotional facial expressions as evidenced by fMRI. *Brain Research*, *1393*, 73–83. https://doi.org/10.1016/j.brainres.2011.04.007

Jeong, J.-W., Diwadkar, V. A., Chugani, C. D., Sinsoongsud, P., Muzik, O., Behen, M. E., Chugani, H. T., & Chugani, D. C. (2011). Congruence of happy and sad emotion in music and faces modifies cortical audiovisual activation. *NeuroImage*, *54*(4), 2973–2982. https://doi.org/10.1016/j.neuroimage.2010.11.017

Jiang, S., Yan, H., Chen, Q., Tian, L., Lu, T., Tan, H.-Y., Yan, J., & Zhang, D. (2015). Cerebral Inefficient Activation in Schizophrenia Patients and Their Unaffected Parents during the N-Back Working Memory Task: A Family fMRI Study. *PLOS ONE*, *10*(8), e0135468. https://doi.org/10.1371/journal.pone.0135468

Jimura, K., Konishi, S., & Miyashita, Y. (2009). Temporal pole activity during perception of sad faces, but not happy faces, correlates with neuroticism trait. *Neuroscience Letters*, *453*(1), 45–48. https://doi.org/10.1016/j.neulet.2009.02.012

Joassin, F., Maurage, P., & Campanella, S. (2011). The neural network sustaining the crossmodal processing of human gender from faces and voices: An fMRI study. *NeuroImage*, *54*(2), 1654–1661. https://doi.org/10.1016/j.neuroimage.2010.08.073

Jogia, J., Haldane, M., Cobb, A., Kumari, V., & Frangou, S. (2008). Pilot investigation of the changes in cortical activation during facial affect recognition with lamotrigine

monotherapy in bipolar disorder. *British Journal of Psychiatry*, *192*(3), 197–201. https://doi.org/10.1192/bjp.bp.107.037960

Johannsen, L., Li, K. Z. H., Chechlacz, M., Bibi, A., Kourtzi, Z., & Wing, A. M. (2013). Functional neuroimaging of the interference between working memory and the control of periodic ankle movement timing. *Neuropsychologia*, *51*(11), 2142–2153. https://doi.org/10.1016/j.neuropsychologia.2013.07.009

Jonides, J., Schumacher, E. H., Smith, E. E., Lauber, E. J., Awh, E., Minoshima, S., & Koeppe, R. A. (1997). Verbal Working Memory Load Affects Regional Brain Activation as Measured by PET. *Journal of Cognitive Neuroscience*, *9*(4), 462–475. https://doi.org/10.1162/jocn.1997.9.4.462

Jung, K., Friston, K. J., Pae, C., Choi, H. H., Tak, S., Choi, Y. K., Park, B., Park, C.-A., Cheong, C., & Park, H.-J. (2018). Effective connectivity during working memory and resting states: A DCM study. *NeuroImage*, *169*, 485–495. https://doi.org/10.1016/j.neuroimage.2017.12.067

Kaminski, J., Gleich, T., Fukuda, Y., Katthagen, T., Gallinat, J., Heinz, A., & Schlagenhauf, F. (2020). Association of Cortical Glutamate and Working Memory Activation in Patients With Schizophrenia: A Multimodal Proton Magnetic Resonance Spectroscopy and Functional Magnetic Resonance Imaging Study. *Biological Psychiatry*, *87*(3), 225–233. https://doi.org/10.1016/j.biopsych.2019.07.011

Kaur, H., Chaudhary, S., Mohanty, S., Sharma, G., Kumaran, S. S., Ghati, N., Bhatia, R., Nehra, A., & Pandey, R. (2022). Comparing cognition, coping skills and vedic personality of individuals practicing yoga, physical exercise or sedentary lifestyle: A cross-sectional fMRI study. *Integrative Medicine Research*, *11*(1), 100750. https://doi.org/10.1016/j.imr.2021.100750

Kempton, M. J., Haldane, M., Jogia, J., Christodoulou, T., Powell, J., Collier, D., Williams, S. C. R., & Frangou, S. (2009). The effects of gender and COMT Val158Met polymorphism on fearful facial affect recognition: A fMRI study. *The International*

Journal of Neuropsychopharmacology, *12*(03), 371. https://doi.org/10.1017/S1461145708009395

Kerns, J. G., Cohen, J. D., MacDonald, A. W., Johnson, M. K., Stenger, V. A., Aizenstein, H., & Carter, C. S. (2005). Decreased Conflict- and Error-Related Activity in the Anterior Cingulate Cortex in Subjects With Schizophrenia. *American Journal of Psychiatry*, *162*(10), 1833–1839. https://doi.org/10.1176/appi.ajp.162.10.1833

Kesler/West, M. L., Andersen, A. H., Smith, C. D., Avison, M. J., Davis, C. E., Kryscio, R. J., & Blonder, L. X. (2001). Neural substrates of facial emotion processing using fMRI. *Cognitive Brain Research*, *11*(2), 213–226. https://doi.org/10.1016/S0926-6410(00)00073-2

Kilts, C. D., Egan, G., Gideon, D. A., Ely, T. D., & Hoffman, J. M. (2003). Dissociable Neural Pathways Are Involved in the Recognition of Emotion in Static and Dynamic Facial Expressions. *NeuroImage*, *18*(1), 156–168. https://doi.org/10.1006/nimg.2002.1323

Kim, C., Johnson, N. F., & Gold, B. T. (2014). Conflict adaptation in prefrontal cortex: Now you see it, now you don’t. *Cortex*, *50*, 76–85. https://doi.org/10.1016/j.cortex.2013.08.011

Kim, J.-J., Kim, M. S., Lee, J. S., Lee, D. S., Lee, M. C., & Kwon, J. S. (2002). Dissociation of Working Memory Processing Associated with Native and Second Languages: PET Investigation. *NeuroImage*, *15*(4), 879–891. https://doi.org/10.1006/nimg.2001.1025

Kim, J.-J., Kwon, J. S., Park, H. J., Youn, T., Kang, D. H., Kim, M. S., Lee, D. S., & Lee, M. C. (2003). Functional Disconnection Between the Prefrontal and Parietal Cortices During Working Memory Processing in Schizophrenia: A [^{15} O]H $_2$ O PET Study. *American Journal of Psychiatry*, *160*(5), 919–923. https://doi.org/10.1176/appi.ajp.160.5.919

Kim, J., Whyte, J., Wang, J., Rao, H., Tang, K. Z., & Detre, J. A. (2006). Continuous ASL perfusion fMRI investigation of higher cognition: Quantification of tonic CBF changes during sustained attention and working memory tasks. *NeuroImage*, *31*(1), 376–385. https://doi.org/10.1016/j.neuroimage.2005.11.035

King, T. Z., Na, S., & Mao, H. (2015). Neural Underpinnings of Working Memory in Adult Survivors of Childhood Brain Tumors. *Journal of the International Neuropsychological Society*, *21*(7), 494–505. https://doi.org/10.1017/S135561771500051X

Kitada, R., Johnsrude, I. S., Kochiyama, T., & Lederman, S. J. (2010). Brain networks involved in haptic and visual identification of facial expressions of emotion: An fMRI study. *NeuroImage*, *49*(2), 1677–1689. https://doi.org/10.1016/j.neuroimage.2009.09.014

Knops, A., Nuerk, H.-C., Fimm, B., Vohn, R., & Willmes, K. (2006). A special role for numbers in working memory? An fMRI study. *NeuroImage*, *29*(1), 1–14. https://doi.org/10.1016/j.neuroimage.2005.07.009

Koch, K., Pauly, K., Kellermann, T., Seiferth, N. Y., Reske, M., Backes, V., Stöcker, T., Shah, N. J., Amunts, K., Kircher, T., Schneider, F., & Habel, U. (2007). Gender differences in the cognitive control of emotion: An fMRI study. *Neuropsychologia*, *45*(12), 2744–2754. https://doi.org/10.1016/j.neuropsychologia.2007.04.012

Köhler, S., Bär, K.-J., & Wagner, G. (2016). Differential involvement of brainstem noradrenergic and midbrain dopaminergic nuclei in cognitive control: NA and Dopaminergic Nuclei and Cognitive Control. *Human Brain Mapping*, *37*(6), 2305–2318. https://doi.org/10.1002/hbm.23173

Koppe, G., Heidel, A., Sammer, G., Bohus, M., Gallhofer, B., Kirsch, P., & Lis, S. (2015). Temporal unpredictability of a stimulus sequence and the processing of neutral and emotional stimuli. *NeuroImage*, *120*, 214–224. https://doi.org/10.1016/j.neuroimage.2015.06.081

Koppelstaetter, F., Poeppel, T. D., Siedentopf, C. M., Ischebeck, A., Verius, M., Haala, I., Mottaghy, F. M., Rhomberg, P., Golaszewski, S., Gotwald, T., Lorenz, I. H., Kolbitsch, C., Felber, S., & Krause, B. J. (2008). Does caffeine modulate verbal working memory processes? An fMRI study. *NeuroImage*, *39*(1), 492–499. https://doi.org/10.1016/j.neuroimage.2007.08.037

Korsnes, M. S., Lövdahl, H., Andersson, S., Björnerud, A., Due-Tönnesen, P., Endestad, T., & Malt, U. F. (2013). Working memory in recurrent brief depression: An fMRI pilot study. *Journal of Affective Disorders*, *149*(1-3), 383–392. https://doi.org/10.1016/j.jad.2013.02.017

Kowalczyk, O. S., Pauls, A. M., Fusté, M., Williams, S. C. R., Hazelgrove, K., Vecchio, C., Seneviratne, G., Pariante, C. M., Dazzan, P., & Mehta, M. A. (2021). Neurocognitive correlates of working memory and emotional processing in postpartum psychosis: An fMRI study. *Psychological Medicine*, *51*(10), 1724–1732. https://doi.org/10.1017/S0033291720000471

Kozasa, E. H., Balardin, J. B., Sato, J. R., Chaim, K. T., Lacerda, S. S., Radvany, J., Mello, L. E. A. M., & Amaro, E. (2018). Effects of a 7-Day Meditation Retreat on the Brain Function of Meditators and Non-Meditators During an Attention Task. *Frontiers in Human Neuroscience*, *12*, 222. https://doi.org/10.3389/fnhum.2018.00222

Kronbichler, L., Stelzig-Schöler, R., Pearce, B.-G., Tschernegg, M., Said-Yürekli, S., Reich, L. A., Weber, S., Aichhorn, W., & Kronbichler, M. (2018). Schizophrenia and Category-Selectivity in the Brain: Normal for Faces but Abnormal for Houses. *Frontiers in Psychiatry*, *9*, 47. https://doi.org/10.3389/fpsyt.2018.00047

Kronhaus, D. M., Lawrence, N. S., Williams, A. M., Frangou, S., Brammer, M. J., Williams, S. C., Andrew, C. M., & Phillips, M. L. (2006). Stroop performance in bipolar disorder: Further evidence for abnormalities in the ventral prefrontal cortex. *Bipolar Disorders*, *8*(1), 28–39. https://doi.org/10.1111/j.1399-5618.2006.00282.x

Krug, A., Markov, V., Eggermann, T., Krach, S., Zerres, K., Stöcker, T., Shah, N. J., Schneider, F., Nöthen, M. M., Treutlein, J., Rietschel, M., & Kircher, T. (2008). Genetic variation in the schizophrenia-risk gene Neuregulin1 correlates with differences in frontal brain activation in a working memory task in healthy individuals. *NeuroImage*, *42*(4), 1569–1576. https://doi.org/10.1016/j.neuroimage.2008.05.058

Kühn, S., Schubert, F., Mekle, R., Wenger, E., Ittermann, B., Lindenberger, U., & Gallinat, J. (2016). Neurotransmitter changes during interference task in anterior cingulate cortex: Evidence from fMRI-guided functional MRS at 3 T. *Brain Structure and Function*, *221*(5), 2541–2551. https://doi.org/10.1007/s00429-015-1057-0

Kumari, V., Aasen, I., Taylor, P., ffytche, D. H., Das, M., Barkataki, I., Goswami, S., O'Connell, P., Howlett, M., Williams, S. C. R., & Sharma, T. (2006). Neural dysfunction and violence in schizophrenia: An fMRI investigation. *Schizophrenia Research*, *84*(1), 144–164. https://doi.org/10.1016/j.schres.2006.02.017

Kumari, V., Peters, E., Guinn, A., Fannon, D., Russell, T., Sumich, A., Kuipers, E., Williams, S. C. R., & ffytche, D. H. (2016). Mapping Depression in Schizophrenia: A Functional Magnetic Resonance Imaging Study. *Schizophrenia Bulletin*, *42*(3), 802–813. https://doi.org/10.1093/schbul/sbv186

Lahr, J., Minkova, L., Tabrizi, S. J., Stout, J. C., Klöppel, S., Scheller, E., & the TrackOn-HD Investigators. (2018). Working Memory-Related Effective Connectivity in Huntington's Disease Patients. *Frontiers in Neurology*, *9*, 370. https://doi.org/10.3389/fneur.2018.00370

Langner, R., & Eickhoff, S. B. (2013). Sustaining attention to simple tasks: A meta-analytic review of the neural mechanisms of vigilant attention. *Psychological Bulletin*, *139*(4), 870–900. https://doi.org/10.1037/a0030694

Lee, K.-U., Khang, H. S., Kim, K.-T., Kim, Y.-J., Kweon, Y.-S., Shin, Y.-W., Kwon, J. S., Ho, S.-H., Garfinkel, S. N., Chae, J.-H., & Liberzon, I. (2008). Distinct processing of

facial emotion of own-race versus other-race. *NeuroReport*, *19*(10), 1021–1025. https://doi.org/10.1097/WNR.0b013e3283052df2

Lesh, T. A., Westphal, A. J., Niendam, T. A., Yoon, J. H., Minzenberg, M. J., Ragland, J. D., Solomon, M., & Carter, C. S. (2013). Proactive and reactive cognitive control and dorsolateral prefrontal cortex dysfunction in first episode schizophrenia. *NeuroImage: Clinical*, *2*, 590–599. https://doi.org/10.1016/j.nicl.2013.04.010

Leung, A. W. S., & Alain, C. (2011). Working memory load modulates the auditory “What” and “Where” neural networks. *NeuroImage*, *55*(3), 1260–1269. https://doi.org/10.1016/j.neuroimage.2010.12.055

Li, J., Zhong, Y., Ma, Z., Wu, Y., Pang, M., Wang, C., Liu, N., Wang, C., & Zhang, N. (2020). Emotion reactivity-related brain network analysis in generalized anxiety disorder: A task fMRI study. *BMC Psychiatry*, *20*(1), 429. https://doi.org/10.1186/s12888-020-02831-6

Li, L., Men, W.-W., Chang, Y.-K., Fan, M.-X., Ji, L., & Wei, G.-X. (2014). Acute Aerobic Exercise Increases Cortical Activity during Working Memory: A Functional MRI Study in Female College Students. *PLoS ONE*, *9*(6), e99222. https://doi.org/10.1371/journal.pone.0099222

Li, L., Zhang, S., Cui, J., Chen, L.-Z., Wang, X., Fan, M., & Wei, G.-X. (2019). Fitness-Dependent Effect of Acute Aerobic Exercise on Executive Function. *Frontiers in Physiology*, *10*, 902. https://doi.org/10.3389/fphys.2019.00902

Li, M., Newton, A. T., Anderson, A. W., Ding, Z., & Gore, J. C. (2019). Characterization of the hemodynamic response function in white matter tracts for event-related fMRI. *Nature Communications*, *10*(1), 1140. https://doi.org/10.1038/s41467-019-09076-2

Li, X., Yi, Z., Lv, Q., Chu, M., Hu, H., Wang, J., Zhang, J., Cheung, E. E. F., & Chan, R. C. K. (2019). Clinical utility of the dual n-back task in schizophrenia: A functional imaging approach. *Psychiatry Research: Neuroimaging*, *284*, 37–44. https://doi.org/10.1016/j.pscychresns.2019.01.002

Liu, M., Liu, C. H., Zheng, S., Zhao, K., & Fu, X. (2021). Reexamining the neural network involved in perception of facial expression: A meta-analysis. *Neuroscience & Biobehavioral Reviews*, *131*, 179–191. https://doi.org/10.1016/j.neubiorev.2021.09.024

Lovén, J., Svärd, J., Ebner, N. C., Herlitz, A., & Fischer, H. (2014). Face gender modulates women's brain activity during face encoding. *Social Cognitive and Affective Neuroscience*, *9*(7), 1000–1005. https://doi.org/10.1093/scan/nst073

Luethi, M. S., Friese, M., Binder, J., Boesiger, P., Luechinger, R., & Rasch, B. (2016). Motivational incentives lead to a strong increase in lateral prefrontal activity after self-control exertion. *Social Cognitive and Affective Neuroscience*, *11*(10), 1618–1626. https://doi.org/10.1093/scan/nsw073

Luo, Y., Qin, S., Fernández, G., Zhang, Y., Klumpers, F., & Li, H. (2014). Emotion perception and executive control interact in the salience network during emotionally charged working memory processing: Examination of Neural Mechanisms on Processing of Emotional WM. *Human Brain Mapping*, *35*(11), 5606–5616. https://doi.org/10.1002/hbm.22573

Madsen, M. K., Mc Mahon, B., Andersen, S. B., Siebner, H. R., Knudsen, G. M., & Fisher, P. M. (2016). Threat-related amygdala functional connectivity is associated with 5-HTTLPR genotype and neuroticism. *Social Cognitive and Affective Neuroscience*, *11*(1), 140–149. https://doi.org/10.1093/scan/nsv098

Malhi, G. S., Lagopoulos, J., Sachdev, P. S., Ivanovski, B., Shnier, R., & Ketter, T. (2007). Is a lack of disgust something to fear? A functional magnetic resonance imaging facial emotion recognition study in euthymic bipolar disorder patients. *Bipolar Disorders*, *9*(4), 345–357. https://doi.org/10.1111/j.1399-5618.2007.00485.x

Malisza, K. L., Allman, A.-A., Shiloff, D., Jakobson, L., Longstaffe, S., & Chudley, A. E. (2005). Evaluation of Spatial Working Memory Function in Children and Adults with Fetal Alcohol Spectrum Disorders: A Functional Magnetic Resonance Imaging Study.

Pediatric Research, *58*(6), 1150–1157. https://doi.org/10.1203/01.pdr.0000185479.92484.a1

Manard, M., François, S., Phillips, C., Salmon, E., & Collette, F. (2017). The neural bases of proactive and reactive control processes in normal aging. *Behavioural Brain Research*, *320*, 504–516. https://doi.org/10.1016/j.bbr.2016.10.026

Manktelow, A. E., Menon, D. K., Sahakian, B. J., & Stamatakis, E. A. (2017). Working Memory after Traumatic Brain Injury: The Neural Basis of Improved Performance with Methylphenidate. *Frontiers in Behavioral Neuroscience*, *11*. https://doi.org/10.3389/fnbeh.2017.00058

Marchand, W. R., Lee, J. N., Garn, C., Thatcher, J., Gale, P., Kreitschitz, S., Johnson, S., & Wood, N. (2011). Aberrant emotional processing in posterior cortical midline structures in bipolar II depression. *Progress in Neuro-Psychopharmacology and Biological Psychiatry*, *35*(7), 1729–1737. https://doi.org/10.1016/j.pnpbp.2011.05.017

Marquand, A. F., Mourão-Miranda, J., Brammer, M. J., Cleare, A. J., & Fu, C. H. Y. (2008). Neuroanatomy of verbal working memory as a diagnostic biomarker for depression: *NeuroReport*, *19*(15), 1507–1511. https://doi.org/10.1097/WNR.0b013e328310425e

Mathis, A., Schunck, T., Erb, G., Namer, I. J., & Luthringer, R. (2009). The effect of aging on the inhibitory function in middle-aged subjects: A functional MRI study coupled with a color-matched Stroop task. *International Journal of Geriatric Psychiatry*, *24*(10), 1062–1071. https://doi.org/10.1002/gps.2222

Matsuo, K., Glahn, D. C., Peluso, M. A. M., Hatch, J. P., Monkul, E. S., Najt, P., Sanches, M., Zamarripa, F., Li, J., Lancaster, J. L., Fox, P. T., Gao, J.-H., & Soares, J. C. (2007). Prefrontal hyperactivation during working memory task in untreated individuals with major depressive disorder. *Molecular Psychiatry*, *12*(2), 158–166. https://doi.org/10.1038/sj.mp.4001894

Maurage, P., Joassin, F., Pesenti, M., Grandin, C., Heeren, A., Philippot, P., & de Timary, P. (2013). The neural network sustaining crossmodal integration is impaired in alcohol-dependence: An fMRI study. *Cortex*, *49*(6), 1610–1626. https://doi.org/10.1016/j.cortex.2012.04.012

McAllister, T. W., Saykin, A. J., Flashman, L. A., Sparling, M. B., Johnson, S. C., Guerin, S. J., Mamourian, A. C., Weaver, J. B., & Yanofsky, N. (1999). Brain activation during working memory 1 month after mild traumatic brain injury: A functional MRI study. *Neurology*, *53*(6), 1300–1300. https://doi.org/10.1212/WNL.53.6.1300

McCloskey, M. S., Phan, K. L., Angstadt, M., Fettich, K. C., Keedy, S., & Coccaro, E. F. (2016). Amygdala hyperactivation to angry faces in intermittent explosive disorder. *Journal of Psychiatric Research*, *79*, 34–41. https://doi.org/10.1016/j.jpsychires.2016.04.006

McGeown, W. J., Shanks, M. F., & Venneri, A. (2008). Prolonged cholinergic enrichment influences regional cortical activation in early Alzheimer’s disease. *Neuropsychiatric Disease and Treatment*, 465. https://doi.org/10.2147/NDT.S2461

Mead, L. A., Mayer, A. R., Bobholz, J. A., Woodley, S. J., Cunningham, J. M., Hammeke, T. A., & Rao, S. M. (2002). Neural basis of the Stroop interference task: Response competition or selective attention? *Journal of the International Neuropsychological Society*, *8*(6), 735–742. https://doi.org/10.1017/S1355617702860015

Meisenzahl, E. M., Scheuerecker, J., Zipse, M., Ufer, S., Wiesmann, M., Frodl, T., Koutsouleris, N., Zetzsche, T., Schmitt, G., Riedel, M., Spellmann, I., Dehning, S., Linn, J., Brückmann, H., & Möller, H. J. (2006). Effects of treatment with the atypical neuroleptic quetiapine on working memory function: A functional MRI follow-up investigation. *European Archives of Psychiatry and Clinical Neuroscience*, *256*(8), 522–531. https://doi.org/10.1007/s00406-006-0687-x

Mencarelli, L., Francesco, N., Davide, M., Arianna, M., Simone, R., Alessandro, R., & Emiliano, S. (2019). Stimuli, presentation modality, and load-specific brain activity patterns during n-back task. *Human Brain Mapping*, hbm.24633. https://doi.org/10.1002/hbm.24633

Mende-Siedlecki, P., Verosky, S. C., Turk-Browne, N. B., & Todorov, A. (2013). Robust Selectivity for Faces in the Human Amygdala in the Absence of Expressions. *Journal of Cognitive Neuroscience*, *25*(12), 2086–2106. https://doi.org/10.1162/jocn_a_00469

Mériau, K., Wartenburger, I., Kazzer, P., Prehn, K., Lammers, C.-H., van der Meer, E., Villringer, A., & Heekeren, H. R. (2006). A neural network reflecting individual differences in cognitive processing of emotions during perceptual decision making. *NeuroImage*, *33*(3), 1016–1027. https://doi.org/10.1016/j.neuroimage.2006.07.031

Michalopoulou, P. G., Surguladze, S., Morley, L. A., Giampietro, V. P., Murray, R. M., & Shergill, S. S. (2008). Facial fear processing and psychotic symptoms in schizophrenia: Functional magnetic resonance imaging study. *British Journal of Psychiatry*, *192*(3), 191–196. https://doi.org/10.1192/bjp.bp.106.032649

Mier, D., Sauer, C., Lis, S., Esslinger, C., Wilhelm, J., Gallhofer, B., & Kirsch, P. (2010). Neuronal correlates of affective theory of mind in schizophrenia out-patients: Evidence for a baseline deficit. *Psychological Medicine*, *40*(10), 1607–1617. https://doi.org/10.1017/S0033291709992133

Migo, E. M., Mitterschiffthaler, M., O'Daly, O., Dawson, G. R., Dourish, C. T., Craig, K. J., Simmons, A., Wilcock, G. K., McCulloch, E., Jackson, S. H. D., Kopelman, M. D., Williams, S. C. R., & Morris, R. G. (2015). Alterations in working memory networks in amnestic mild cognitive impairment. *Aging, Neuropsychology, and Cognition*, *22*(1), 106–127. https://doi.org/10.1080/13825585.2014.894958

Milham, M. P., Banich, M. T., Webb, A., Barad, V., Cohen, N. J., Wszalek, T., & Kramer, A. F. (2001). The relative involvement of anterior cingulate and prefrontal cortex in

attentional control depends on nature of conflict. *Cognitive Brain Research*, *12*(3), 467–473. https://doi.org/10.1016/S0926-6410(01)00076-3

Miró-Padilla, A., Bueichekú, E., Ventura-Campos, N., Flores-Compañ, M.-J., Parcet, M. A., & Ávila, C. (2019). Long-term brain effects of N-back training: An fMRI study. *Brain Imaging and Behavior*, *13*(4), 1115–1127. https://doi.org/10.1007/s11682-018-9925-x

Miskowiak, K. W., Glerup, L., Vestbo, C., Harmer, C. J., Reinecke, A., Macoveanu, J., Siebner, H. R., Kessing, L. V., & Vinberg, M. (2015). Different neural and cognitive response to emotional faces in healthy monozygotic twins at risk of depression. *Psychological Medicine*, *45*(7), 1447–1458. https://doi.org/10.1017/S0033291714002542

Mitchell, R. L. C. (2005). The BOLD response during Stroop task-like inhibition paradigms: Effects of task difficulty and task-relevant modality. *Brain and Cognition*, *59*(1), 23–37. https://doi.org/10.1016/j.bandc.2005.04.001

Monks, P. J., Thompson, J. M., Bullmore, E. T., Suckling, J., Brammer, M. J., Williams, S. C., Simmons, A., Giles, N., Lloyd, A. J., Louise Harrison, C., Seal, M., Murray, R. M., Nicol Ferrier, I., Young, A. H., & Curtis, V. A. (2004). A functional MRI study of working memory task in euthymic bipolar disorder: Evidence for task-specific dysfunction. *Bipolar Disorders*, *6*(6), 550–564. https://doi.org/10.1111/j.1399-5618.2004.00147.x

Morgenroth, E., Orlov, N., Lythgoe, D. J., Stone, J. M., Barker, H., Munro, J., Eysenck, M., & Allen, P. (2019). Altered relationship between prefrontal glutamate and activation during cognitive control in people with high trait anxiety. *Cortex*, *117*, 53–63. https://doi.org/10.1016/j.cortex.2019.02.021

Morr, M., Lieberz, J., Dobbelstein, M., Philipsen, A., Hurlemann, R., & Scheele, D. (2021). Insula reactivity mediates subjective isolation stress in alexithymia. *Scientific Reports*, *11*(1), 15326. https://doi.org/10.1038/s41598-021-94799-w

Müller, V. I., Höhner, Y., & Eickhoff, S. B. (2018). Influence of task instructions and stimuli on the neural network of face processing: An ALE meta-analysis. *Cortex*, *103*, 240–255. https://doi.org/10.1016/j.cortex.2018.03.011

Nakao, T., Nakagawa, A., Yoshiura, T., Nakatani, E., Nabeyama, M., Yoshizato, C., Kudoh, A., Tada, K., Yoshioka, K., & Kawamoto, M. (2005). A functional MRI comparison of patients with obsessive–compulsive disorder and normal controls during a Chinese character Stroop task. *Psychiatry Research: Neuroimaging*, *139*(2), 101–114. https://doi.org/10.1016/j.pscychresns.2004.12.004

Narumoto, J., Okada, T., Sadato, N., Fukui, K., & Yonekura, Y. (2001). Attention to emotion modulates fMRI activity in human right superior temporal sulcus. *Cognitive Brain Research*, *12*(2), 225–231. https://doi.org/10.1016/S0926-6410(01)00053-2

Narumoto, J., Yamada, H., Iidaka, T., Sadato, N., Fukui, K., Itoh, H., & Yonekura, Y. (2000). Brain regions involved in verbal or non-verbal aspects of facial emotion recognition: *NeuroReport*, *11*(11), 2571–2574. https://doi.org/10.1097/00001756-200008030-00044

Ning, R. (2021). How language proficiency influences stroop effect and reverse-stroop effect: A functional magnetic resonance imaging study. *Journal of Neurolinguistics*, *60*, 101027. https://doi.org/10.1016/j.jneuroling.2021.101027

Norris, D. G., Zysset, S., Mildner, T., & Wiggins, C. J. (2002). An Investigation of the Value of Spin-Echo-Based fMRI Using a Stroop Color–Word Matching Task and EPI at 3 T. *NeuroImage*, *15*(3), 719–726. https://doi.org/10.1006/nimg.2001.1005

O'Nions, E. J. P., Dolan, R. J., & Roiser, J. P. (2011). Serotonin Transporter Genotype Modulates Subgenual Response to Fearful Faces Using an Incidental Task. *Journal of Cognitive Neuroscience*, *23*(11), 3681–3693. https://doi.org/10.1162/jocn_a_00055

Overbeek, G., Gawne, T. J., Reid, M. A., Salibi, N., Kraguljac, N. V., White, D. M., & Lahti, A. C. (2019). Relationship Between Cortical Excitation and Inhibition and Task-Induced Activation and Deactivation: A Combined Magnetic Resonance Spectroscopy

and Functional Magnetic Resonance Imaging Study at 7T in First-Episode Psychosis. *Biological Psychiatry: Cognitive Neuroscience and Neuroimaging*, *4*(2), 121–130. https://doi.org/10.1016/j.bpsc.2018.10.002

Palm, M. E., Elliott, R., McKie, S., Deakin, J. F. W., & Anderson, I. M. (2011). Attenuated responses to emotional expressions in women with generalized anxiety disorder. *Psychological Medicine*, *41*(5), 1009–1018. https://doi.org/10.1017/S0033291710001455

Papalini, S., Michels, F., Kohn, N., Wegman, J., van Hemert, S., Roelofs, K., Arias-Vasquez, A., & Aarts, E. (2019). Stress matters: Randomized controlled trial on the effect of probiotics on neurocognition. *Neurobiology of Stress*, *10*, 100141. https://doi.org/10.1016/j.ynstr.2018.100141

Paradiso, S., Robinson, R. G., Boles Ponto, L. L., Watkins, G. L., & Hichwa, R. D. (2003). Regional Cerebral Blood Flow Changes During Visually Induced Subjective Sadness in Healthy Elderly Persons. *The Journal of Neuropsychiatry and Clinical Neurosciences*, *15*(1), 35–44. https://doi.org/10.1176/jnp.15.1.35

Pardo, J. V., Pardo, P. J., Janer, K. W., & Raichle, M. E. (1990). The anterior cingulate cortex mediates processing selection in the Stroop attentional conflict paradigm. *Proceedings of the National Academy of Sciences*, *87*(1), 256–259. https://doi.org/10.1073/pnas.87.1.256

Park, H. Y., Yun, J.-Y., Shin, N. Y., Kim, S.-Y., Jung, W. H., Shin, Y. S., Cho, K. I. K., Yoon, Y. B., Lim, K.-O., Kim, S. N., & Kwon, J. S. (2016). Decreased neural response for facial emotion processing in subjects with high genetic load for schizophrenia. *Progress in Neuro-Psychopharmacology and Biological Psychiatry*, *71*, 90–96. https://doi.org/10.1016/j.pnpbp.2016.06.014

Park, J.-W., Kim, Y.-T., Yun, B.-J., Jin, S.-U., Lee, S.-H., Ahn, S.-H., Min, Y., Jung, T.-D., Lee, H. J., & Chang, Y. (2016). Stereoscopic 3D objects evoke stronger saliency for

nonverbal working memory: An fMRI study. *International Journal of Imaging Systems and Technology*, *26*(1), 76–84. https://doi.org/10.1002/ima.22159

Park, M.-S., Kim, S.-H., Sohn, S., Kim, G.-J., Kim, Y.-K., & Sohn, J.-H. (2015). Brain activation during processing of angry facial expressions in patients with alcohol dependency. *Journal of Physiological Anthropology*, *34*(1), 6. https://doi.org/10.1186/s40101-015-0046-6

Park, M.-S., Sohn, S., Park, J.-E., Kim, S.-H., Yu, I. K., & Sohn, J.-H. (2011). Brain functions associated with verbal working memory tasks among young males with alcohol use disorders: Brain functions and verbal working memory tasks. *Scandinavian Journal of Psychology*, *52*(1), 1–7. https://doi.org/10.1111/j.1467-9450.2010.00848.x

Passamonti, L., Cerasa, A., Liguori, M., Gioia, M. C., Valentino, P., Nistico, R., Quattrone, A., & Fera, F. (2009). Neurobiological mechanisms underlying emotional processing in relapsing-remitting multiple sclerosis. *Brain*, *132*(12), 3380–3391. https://doi.org/10.1093/brain/awp095

Pegors, T. K., Kable, J. W., Chatterjee, A., & Epstein, R. A. (2015). Common and Unique Representations in pFC for Face and Place Attractiveness. *Journal of Cognitive Neuroscience*, *27*(5), 959–973. https://doi.org/10.1162/jocn_a_00777

Peven, J. C., Litz, G. A., Brown, B., Xie, X., Grove, G. A., Watt, J. C., & Erickson, K. I. (2019). Higher Cardiorespiratory Fitness is Associated with Reduced Functional Brain Connectivity During Performance of the Stroop Task. *Brain Plasticity*, *5*(1), 57–67. https://doi.org/10.3233/BPL-190085

Pfefferbaum, A., Desmond, J. E., Galloway, C., Menon, V., Glover, G. H., & Sullivan, E. V. (2001). Reorganization of Frontal Systems Used by Alcoholics for Spatial Working Memory: An fMRI Study. *NeuroImage*, *14*(1), 7–20. https://doi.org/10.1006/nimg.2001.0785

Philip, N. S., Sweet, L. H., Tyrka, A. R., Carpenter, S. L., Albright, S. E., Price, L. H., & Carpenter, L. L. (2016). Exposure to childhood trauma is associated with altered n-back

activation and performance in healthy adults: Implications for a commonly used working memory task. *Brain Imaging and Behavior*, *10*(1), 124–135. https://doi.org/10.1007/s11682-015-9373-9

Piai, V., Roelofs, A., Acheson, D. J., & Takashima, A. (2013). Attention for speaking: Domain-general control from the anterior cingulate cortex in spoken word production. *Frontiers in Human Neuroscience*, *7*. https://doi.org/10.3389/fnhum.2013.00832

Poldrack, R. A., Baker, C. I., Durnez, J., Gorgolewski, K. J., Matthews, P. M., Munafò, M. R., Nichols, T. E., Poline, J.-B., Vul, E., & Yarkoni, T. (2017). Scanning the horizon: Towards transparent and reproducible neuroimaging research. *Nature Reviews Neuroscience*, *18*(2), 115–126. https://doi.org/10.1038/nrn.2016.167

Polk, T. A., Drake, R. M., Jonides, J. J., Smith, M. R., & Smith, E. E. (2008). Attention Enhances the Neural Processing of Relevant Features and Suppresses the Processing of Irrelevant Features in Humans: A Functional Magnetic Resonance Imaging Study of the Stroop Task. *Journal of Neuroscience*, *28*(51), 13786–13792. https://doi.org/10.1523/JNEUROSCI.1026-08.2008

Pomarol-Clotet, E., Salvador, R., Sarró, S., Gomar, J., Vila, F., Martínez, Á., Guerrero, A., Ortiz-Gil, J., Sans-Sansa, B., Capdevila, A., Cebamanos, J. M., & McKenna, P. J. (2008). Failure to deactivate in the prefrontal cortex in schizophrenia: Dysfunction of the default mode network? *Psychological Medicine*, *38*(8), 1185–1193. https://doi.org/10.1017/S0033291708003565

Pompei, F., Jogia, J., Tatarelli, R., Girardi, P., Rubia, K., Kumari, V., & Frangou, S. (2011). Familial and disease specific abnormalities in the neural correlates of the Stroop Task in Bipolar Disorder. *NeuroImage*, *56*(3), 1677–1684. https://doi.org/10.1016/j.neuroimage.2011.02.052

Portes, B., Balardin, J. B., Lacerda, S., Pires, F., Tobo, P., Barrichello, C., Peterson, J., Sanches, L. R., Sanches-Rocha, L., Amaro, E., & Kozasa, E. H. (2019). The effects of perceived chronic stress on the fMRI correlates of attentional control in women

managers. *Archives of Women’s Mental Health*, *22*(3), 375–381. https://doi.org/10.1007/s00737-018-0902-6

Potenza, M. N., Leung, H.-C., Blumberg, H. P., Peterson, B. S., Fulbright, R. K., Lacadie, C. M., Skudlarski, P., & Gore, J. C. (2003). An fMRI Stroop Task Study of Ventromedial Prefrontal Cortical Function in Pathological Gamblers. *American Journal of Psychiatry*, *160*(11), 1990–1994. https://doi.org/10.1176/appi.ajp.160.11.1990

Prakash, R. S., Erickson, K. I., Colcombe, S. J., Kim, J. S., Voss, M. W., & Kramer, A. F. (2009). Age-related differences in the involvement of the prefrontal cortex in attentional control. *Brain and Cognition*, *71*(3), 328–335. https://doi.org/10.1016/j.bandc.2009.07.005

Preckel, K., Trautwein, F.-M., Paulus, F. M., Kirsch, P., Krach, S., Singer, T., & Kanske, P. (2019). Neural mechanisms of affective matching across faces and scenes. *Scientific Reports*, *9*(1), 1492. https://doi.org/10.1038/s41598-018-37163-9

Prochnow, D., Brunheim, S., Steinhäuser, L., & Seitz, R. J. (2014). Reasoning about the implications of facial expressions: A behavioral and fMRI study on low and high social impact. *Brain and Cognition*, *90*, 165–173. https://doi.org/10.1016/j.bandc.2014.07.004

Prochnow, D., Kossack, H., Brunheim, S., Müller, K., Wittsack, H.-J., Markowitsch, H.-J., & Seitz, R. J. (2013). Processing of subliminal facial expressions of emotion: A behavioral and fMRI study. *Social Neuroscience*, *8*(5), 448–461. https://doi.org/10.1080/17470919.2013.812536

Purmann, S., & Pollmann, S. (2015). Adaptation to recent conflict in the classical color-word Stroop-task mainly involves facilitation of processing of task-relevant information. *Frontiers in Human Neuroscience*, *9*. https://doi.org/10.3389/fnhum.2015.00088

Qin, S., Hermans, E. J., van Marle, H. J. F., Luo, J., & Fernández, G. (2009). Acute Psychological Stress Reduces Working Memory-Related Activity in the Dorsolateral Prefrontal Cortex. *Biological Psychiatry*, *66*(1), 25–32. https://doi.org/10.1016/j.biopsych.2009.03.006

Ragland, J. D., Turetsky, B. I., Gur, R. C., Gunning-Dixon, F., Turner, T., Schroeder, L., Chan, R., & Gur, R. E. (2015). *Working Memory for Complex Figures: An fMRI Comparison of Letter and Fractal n-Back Tasks*. 23.

Rämä, P., Martinkauppi, S., Linnankoski, I., Koivisto, J., Aronen, H. J., & Carlson, S. (2001). Working Memory of Identification of Emotional Vocal Expressions: An fMRI Study. *NeuroImage*, *13*(6), 1090–1101. https://doi.org/10.1006/nimg.2001.0777

Ravnkilde, B., Videbech, P., Rosenberg, R., Gjedde, A., & Gade, A. (2002). Putative Tests of Frontal Lobe Function: A PET-Study of Brain Activation During Stroop's Test and Verbal Fluency. *Journal of Clinical and Experimental Neuropsychology*, *24*(4), 534–547. https://doi.org/10.1076/jcen.24.4.534.1033

Reisch, L. M., Wegrzyn, M., Woermann, F. G., Bien, C. G., & Kissler, J. (2020). Negative content enhances stimulus-specific cerebral activity during free viewing of pictures, faces, and words. *Human Brain Mapping*, *41*(15), 4332–4354. https://doi.org/10.1002/hbm.25128

Richter, S., Gorny, X., Machts, J., Behnisch, G., Wüstenberg, T., Herbort, M. C., Münte, T. F., Seidenbecher, C. I., & Schott, B. H. (2013). Effects of AKAP5 Pro100Leu Genotype on Working Memory for Emotional Stimuli. *PLoS ONE*, *8*(1), e55613. https://doi.org/10.1371/journal.pone.0055613

Roberts, K. L., & Hall, D. A. (2008). Examining a Supramodal Network for Conflict Processing: A Systematic Review and Novel Functional Magnetic Resonance Imaging Data for Related Visual and Auditory Stroop Tasks. *Journal of Cognitive Neuroscience*, *20*(6), 1063–1078. https://doi.org/10.1162/jocn.2008.20074

Rodríguez-Cano, E., Alonso-Lana, S., Sarró, S., Fernández-Corcuera, P., Goikolea, J. M., Vieta, E., Maristany, T., Salvador, R., McKenna, P. J., & Pomarol-Clotet, E. (2017). Differential failure to deactivate the default mode network in unipolar and bipolar depression. *Bipolar Disorders*, *19*(5), 386–395. https://doi.org/10.1111/bdi.12517

Rodríguez-Cano, E., Sarró, S., Monté, G. C., Maristany, T., Salvador, R., McKenna, P. J., & Pomarol-Clotet, E. (2014). Evidence for structural and functional abnormality in the subgenual anterior cingulate cortex in major depressive disorder. *Psychological Medicine*, *44*(15), 3263–3273. https://doi.org/10.1017/S0033291714000841

Rosengarth, K., Kleinjung, T., Langguth, B., Landgrebe, M., Lohaus, F., Greenlee, M. W., Hajak, G., Schmidt, N. O., & Schecklmann, M. (2021). Altered brain responses to emotional facial expressions in tinnitus patients. In *Progress in Brain Research* (Vol. 262, pp. 189–207). Elsevier. https://doi.org/10.1016/bs.pbr.2021.01.026

Rossion, B., Hanseeuw, B., & Dricot, L. (2012). Defining face perception areas in the human brain: A large-scale factorial fMRI face localizer analysis. *Brain and Cognition*, *79*(2), 138–157. https://doi.org/10.1016/j.bandc.2012.01.001

Rottschy, C., Langner, R., Dogan, I., Reetz, K., Laird, A. R., Schulz, J. B., Fox, P. T., & Eickhoff, S. B. (2012). Modelling neural correlates of working memory: A coordinate-based meta-analysis. *NeuroImage*, *60*(1), 830–846. https://doi.org/10.1016/j.neuroimage.2011.11.050

Rubino, V., Blasi, G., Latorre, V., Fazio, L., d'Errico, I., Mazzola, V., Caforio, G., Nardini, M., Popolizio, T., Hariri, A., Arciero, G., & Bertolino, A. (2007). Activity in medial prefrontal cortex during cognitive evaluation of threatening stimuli as a function of personality style. *Brain Research Bulletin*, *74*(4), 250–257. https://doi.org/10.1016/j.brainresbull.2007.06.019

Ruff, C. C., Woodward, T. S., Laurens, K. R., & Liddle, P. F. (2001). The Role of the Anterior Cingulate Cortex in Conflict Processing: Evidence from Reverse Stroop Interference. *NeuroImage*, *14*(5), 1150–1158. https://doi.org/10.1006/nimg.2001.0893

Rymarczyk, K., Żurawski, Ł., Jankowiak-Siuda, K., & Szatkowska, I. (2019). Empathy in Facial Mimicry of Fear and Disgust: Simultaneous EMG-fMRI Recordings During Observation of Static and Dynamic Facial Expressions. *Frontiers in Psychology*, *10*, 701. https://doi.org/10.3389/fpsyg.2019.00701

Sagaspe, P., Schwartz, S., & Vuilleumier, P. (2011). Fear and stop: A role for the amygdala in motor inhibition by emotional signals. *NeuroImage*, *55*(4), 1825–1835. https://doi.org/10.1016/j.neuroimage.2011.01.027

Salavert, J., Ramos-Quiroga, J. A., Moreno-Alcázar, A., Caseras, X., Palomar, G., Radua, J., Bosch, R., Salvador, R., McKenna, P. J., Casas, M., & Pomarol-Clotet, E. (2018). Functional Imaging Changes in the Medial Prefrontal Cortex in Adult ADHD. *Journal of Attention Disorders*, *22*(7), 679–693. https://doi.org/10.1177/1087054715611492

Salimi-Khorshidi, G., Smith, S. M., Keltner, J. R., Wager, T. D., & Nichols, T. E. (2009). Meta-analysis of neuroimaging data: A comparison of image-based and coordinate-based pooling of studies. *NeuroImage*, *45*(3), 810–823. https://doi.org/10.1016/j.neuroimage.2008.12.039

Salloum, J. B., Ramchandani, V. A., Bodurka, J., Rawlings, R., Momenan, R., George, D., & Hommer, D. W. (2007). Blunted Rostral Anterior Cingulate Response During a Simplified Decoding Task of Negative Emotional Facial Expressions in Alcoholic Patients. *Alcoholism: Clinical and Experimental Research*, *31*(9), 1490–1504. https://doi.org/10.1111/j.1530-0277.2007.00447.x

Sambataro, F., Dimalta, S., Di Giorgio, A., Taurisano, P., Blasi, G., Scarabino, T., Giannatempo, G., Nardini, M., & Bertolino, A. (2006). Preferential responses in amygdala and insula during presentation of facial contempt and disgust. *European Journal of Neuroscience*, *24*(8), 2355–2362. https://doi.org/10.1111/j.1460-9568.2006.05120.x

Sánchez-Carrión, R., Gómez, P. V., Junqué, C., Fernández-Espejo, D., Falcon, C., Bargalló, N., Roig-Rovira, T., Enseñat-Cantallops, A., & Bernabeu, M. (2008). Frontal Hypoactivation on Functional Magnetic Resonance Imaging in Working Memory after Severe Diffuse Traumatic Brain Injury. *Journal of Neurotrauma*, *25*(5), 479–494. https://doi.org/10.1089/neu.2007.0417

Sapara, A., ffytche, D. H., Birchwood, M., Cooke, M. A., Fannon, D., Williams, S. C. R., Kuipers, E., & Kumari, V. (2014). Preservation and compensation: The functional neuroanatomy of insight and working memory in schizophrenia. *Schizophrenia Research*, *152*(1), 201–209. https://doi.org/10.1016/j.schres.2013.11.026

Scheller, E., Peter, J., Schumacher, L. V., Lahr, J., Mader, I., Kaller, C. P., & Klöppel, S. (2017). APOE moderates compensatory recruitment of neuronal resources during working memory processing in healthy older adults. *Neurobiology of Aging*, *56*, 127–137. https://doi.org/10.1016/j.neurobiolaging.2017.04.015

Scheuerecker, J., Frodl, T., Koutsouleris, N., Zetzsche, T., Wiesmann, M., Kleemann, A. M., Brückmann, H., Schmitt, G., Möller, H.-J., & Meisenzahl, E. M. (2007). Cerebral Differences in Explicit and Implicit Emotional Processing – An fMRI Study. *Neuropsychobiology*, *56*(1), 32–39. https://doi.org/10.1159/000110726

Scheuerecker, J., Ufer, S., Zipse, M., Frodl, T., Koutsouleris, N., Zetzsche, T., Wiesmann, M., Albrecht, J., Brückmann, H., Schmitt, G., Möller, H.-J., & Meisenzahl, E. M. (2008). Cerebral changes and cognitive dysfunctions in medication-free schizophrenia – An fMRI study. *Journal of Psychiatric Research*, *42*(6), 469–476. https://doi.org/10.1016/j.jpsychires.2007.04.001

Schlagenhauf, F., Wüstenberg, T., Schmack, K., Dinges, M., Wrase, J., Koslowski, M., Kienast, T., Bauer, M., Gallinat, J., Juckel, G., & Heinz, A. (2008). Switching schizophrenia patients from typical neuroleptics to olanzapine: Effects on BOLD response during attention and working memory. *European Neuropsychopharmacology*, *18*(8), 589–599. https://doi.org/10.1016/j.euroneuro.2008.04.013

Schmidt, C., Collette, F., Reichert, C. F., Maire, M., Vandewalle, G., Peigneux, P., & Cajochen, C. (2015). Pushing the Limits: Chronotype and Time of Day Modulate Working Memory-Dependent Cerebral Activity. *Frontiers in Neurology*, *6*. https://doi.org/10.3389/fneur.2015.00199

Schmidt, C., Peigneux, P., Leclercq, Y., Sterpenich, V., Vandewalle, G., Phillips, C., Berthomier, P., Berthomier, C., Tinguely, G., Gais, S., Schabus, M., Desseilles, M., Dang-Vu, T., Salmon, E., Degueldre, C., Balteau, E., Luxen, A., Cajochen, C., Maquet, P., & Collette, F. (2012). Circadian Preference Modulates the Neural Substrate of Conflict Processing across the Day. *PLoS ONE*, *7*(1), e29658. https://doi.org/10.1371/journal.pone.0029658

Schneider, F., Habel, U., Reske, M., Kellermann, T., Stöcker, T., Shah, N. J., Zilles, K., Braus, D. F., Schmitt, A., Schlösser, R., Wagner, M., Frommann, I., Kircher, T., Rapp, A., Meisenzahl, E., Ufer, S., Ruhrmann, S., Thienel, R., Sauer, H., … Gaebel, W. (2007). Neural correlates of working memory dysfunction in first-episode schizophrenia patients: An fMRI multi-center study. *Schizophrenia Research*, *89*(1-3), 198–210. https://doi.org/10.1016/j.schres.2006.07.021

Schneiders, J. A., Opitz, B., Krick, C. M., & Mecklinger, A. (2011). Separating Intra-Modal and Across-Modal Training Effects in Visual Working Memory: An fMRI Investigation. *Cerebral Cortex*, *21*(11), 2555–2564. https://doi.org/10.1093/cercor/bhr037

Schulte, T., Müller-Oehring, E. M., Sullivan, E. V., & Pfefferbaum, A. (2012). Synchrony of Corticostriatal-Midbrain Activation Enables Normal Inhibitory Control and Conflict Processing in Recovering Alcoholic Men. *Biological Psychiatry*, *71*(3), 269–278. https://doi.org/10.1016/j.biopsych.2011.10.022

Schulte, T., Müller-Oehring, E. M., Vinco, S., Hoeft, F., Pfefferbaum, A., & Sullivan, E. V. (2009). Double dissociation between action-driven and perception-driven conflict resolution invoking anterior versus posterior brain systems. *NeuroImage*, *48*(2), 381–390. https://doi.org/10.1016/j.neuroimage.2009.06.058

Schultz, J., & Pilz, K. S. (2009). Natural facial motion enhances cortical responses to faces. *Experimental Brain Research*, *194*(3), 465–475. https://doi.org/10.1007/s00221-009-1721-9

Schurz, M., Radua, J., Tholen, M. G., Maliske, L., Margulies, D. S., Mars, R. B., Sallet, J., & Kanske, P. (2021). Toward a hierarchical model of social cognition: A neuroimaging meta-analysis and integrative review of empathy and theory of mind. *Psychological Bulletin*, *147*(3), 293–327. https://doi.org/10.1037/bul0000303

Seara-Cardoso, A., Sebastian, C. L., Viding, E., & Roiser, J. P. (2016). Affective resonance in response to others' emotional faces varies with affective ratings and psychopathic traits in amygdala and anterior insula. *Social Neuroscience*, *11*(2), 140–152. https://doi.org/10.1080/17470919.2015.1044672

Seitz, R. J., Schäfer, R., Scherfeld, D., Friederichs, S., Popp, K., Wittsack, H.-J., Azari, N. P., & Franz, M. (2008). Valuating other people's emotional face expression: A combined functional magnetic resonance imaging and electroencephalography study. *Neuroscience*, *152*(3), 713–722. https://doi.org/10.1016/j.neuroscience.2007.10.066

Seo, J., Kim, S.-H., Kim, Y.-T., Song, H., Lee, J., Kim, S.-H., Han, S. W., Nam, E. J., Kim, S.-K., Lee, H. J., Lee, S.-J., & Chang, Y. (2012). Working Memory Impairment in Fibromyalgia Patients Associated with Altered Frontoparietal Memory Network. *PLoS ONE*, *7*(6), e37808. https://doi.org/10.1371/journal.pone.0037808

Seo, J., Lee, B.-K., Jin, S.-U., Park, J. W., Kim, Y.-T., Ryeom, H.-K., Lee, J., Suh, K. J., Kim, S. H., Park, S.-J., Jeong, K. S., Ham, J.-O., Kim, Y., & Chang, Y. (2014). Lead-Induced Impairments in the Neural Processes Related to Working Memory Function. *PLoS ONE*, *9*(8), e105308. https://doi.org/10.1371/journal.pone.0105308

Seok Jeong, B., Soo Kwon, J., Yoon Kim, S., Lee, C., Youn, T., Moon, C.-H., & Yoon Kim, C. (2005). Functional imaging evidence of the relationship between recurrent psychotic episodes and neurodegenerative course in schizophrenia. *Psychiatry Research: Neuroimaging*, *139*(3), 219–228. https://doi.org/10.1016/j.pscychresns.2004.01.008

Shashidhara, S., Spronkers, F. S., & Erez, Y. (2020). Individual-subject Functional Localization Increases Univariate Activation but Not Multivariate Pattern Discriminability

in the “Multiple-demand” Frontoparietal Network. *Journal of Cognitive Neuroscience*, *32*(7), 1348–1368. https://doi.org/10.1162/jocn_a_01554

Sheu, L. K., Jennings, J. R., & Gianaros, P. J. (2012). Test-retest reliability of an fMRI paradigm for studies of cardiovascular reactivity: Test-retest reliability of an fMRI paradigm. *Psychophysiology*, *49*(7), 873–884. https://doi.org/10.1111/j.1469-8986.2012.01382.x

Shin, G., & Kim, C. (2015). Neural correlates of cognitive style and flexible cognitive control. *NeuroImage*, *113*, 78–85. https://doi.org/10.1016/j.neuroimage.2015.03.046

Silton, R. L., Heller, W., Towers, D. N., Engels, A. S., Spielberg, J. M., Edgar, J. C., Sass, S. M., Stewart, J. L., Sutton, B. P., Banich, M. T., & Miller, G. A. (2010). The time course of activity in dorsolateral prefrontal cortex and anterior cingulate cortex during top-down attentional control. *NeuroImage*, *50*(3), 1292–1302. https://doi.org/10.1016/j.neuroimage.2009.12.061

Smits, M., Dippel, D. W. J., Houston, G. C., Wielopolski, P. A., Koudstaal, P. J., Hunink, M. G. M., & van der Lugt, A. (2009). Postconcussion syndrome after minor head injury: Brain activation of working memory and attention. *Human Brain Mapping*, *30*(9), 2789–2803. https://doi.org/10.1002/hbm.20709

Snoek, L., van der Miesen, M. M., Beemsterboer, T., van der Leij, A., Eigenhuis, A., & Steven Scholte, H. (2021). The Amsterdam Open MRI Collection, a set of multimodal MRI datasets for individual difference analyses. *Scientific Data*, *8*(1), 85. https://doi.org/10.1038/s41597-021-00870-6

Song, Y., & Hakoda, Y. (2015). An fMRI study of the functional mechanisms of Stroop/reverse-Stroop effects. *Behavioural Brain Research*, *290*, 187–196. https://doi.org/10.1016/j.bbr.2015.04.047

Spilka, M. J., Arnold, A. E., & Goghari, V. M. (2015). Functional activation abnormalities during facial emotion perception in schizophrenia patients and nonpsychotic relatives.

Schizophrenia Research, *168*(1-2), 330–337. https://doi.org/10.1016/j.schres.2015.07.012

Spreng, R. N., DuPre, E., Selarka, D., Garcia, J., Gojkovic, S., Mildner, J., Luh, W.-M., & Turner, G. R. (2014). Goal-Congruent Default Network Activity Facilitates Cognitive Control. *Journal of Neuroscience*, *34*(42), 14108–14114. https://doi.org/10.1523/JNEUROSCI.2815-14.2014

Sprengelmeyer, R., Rausch, M., Eysel, U. T., & Przuntek, H. (1998). Neural structures associated with recognition of facial expressions of basic emotions. *Proceedings of the Royal Society of London. Series B: Biological Sciences*, *265*(1409), 1927–1931. https://doi.org/10.1098/rspb.1998.0522

Steel, C., Haworth, E. J., Peters, E., Hemsley, D. R., Sharma, T., Gray, J. A., Pickering, A., Gregory, L., Simmons, A., Bullmore, E. T., & Williams, S. C. R. (2001). Neuroimaging correlates of negative priming: *Neuroreport*, *12*(16), 3619–3624. https://doi.org/10.1097/00001756-200111160-00049

Stevens, J. S., Jovanovic, T., Fani, N., Ely, T. D., Glover, E. M., Bradley, B., & Ressler, K. J. (2013). Disrupted amygdala-prefrontal functional connectivity in civilian women with posttraumatic stress disorder. *Journal of Psychiatric Research*, *47*(10), 1469–1478. https://doi.org/10.1016/j.jpsychires.2013.05.031

Stoodley, C. J., Valera, E. M., & Schmahmann, J. D. (2012). Functional topography of the cerebellum for motor and cognitive tasks: An fMRI study. *NeuroImage*, *59*(2), 1560–1570. https://doi.org/10.1016/j.neuroimage.2011.08.065

Surguladze, S. A., Brammer, M. J., Young, A. W., Andrew, C., Travis, M. J., Williams, S. C. R., & Phillips, M. L. (2003). A preferential increase in the extrastriate response to signals of danger. *NeuroImage*, *19*(4), 1317–1328. https://doi.org/10.1016/S1053-8119(03)00085-5

Surguladze, S. A., Chu, E. M., Marshall, N., Evans, A., Anilkumar, A. P., Timehin, C., McDonald, C., Ecker, C., Phillips, M. L., & David, A. S. (2011). Emotion processing in

schizophrenia: fMRI study of patients treated with risperidone long-acting injections or conventional depot medication. *Journal of Psychopharmacology*, *25*(6), 722–733. https://doi.org/10.1177/0269881110363316

Surguladze, S. A., El-Hage, W., Dalgleish, T., Radua, J., Gohier, B., & Phillips, M. L. (2010). Depression is associated with increased sensitivity to signals of disgust: A functional magnetic resonance imaging study. *Journal of Psychiatric Research*, *44*(14), 894–902. https://doi.org/10.1016/j.jpsychires.2010.02.010

Surguladze, S. A., Elkin, A., Ecker, C., Kalidindi, S., Corsico, A., Giampietro, V., Lawrence, N., Deeley, Q., Murphy, D. G. M., Kucharska-Pietura, K., Russell, T. A., McGuffin, P., Murray, R., & Phillips, M. L. (2008). Genetic variation in the serotonin transporter modulates neural system-wide response to fearful faces. *Genes, Brain and Behavior*, *7*(5), 543–551. https://doi.org/10.1111/j.1601-183X.2008.00390.x

Szabó, E., Galambos, A., Kocsel, N., Édes, A. E., Pap, D., Zsombók, T., Kozák, L. R., Bagdy, G., Kökönyei, G., & Juhász, G. (2019). Association between migraine frequency and neural response to emotional faces: An fMRI study. *NeuroImage: Clinical*, *22*, 101790. https://doi.org/10.1016/j.nicl.2019.101790

Szaflarski, J. P., Allendorfer, J. B., Nenert, R., LaFrance, W. C., Barkan, H. I., DeWolfe, J., Pati, S., Thomas, A. E., & Ver Hoef, L. (2018). Facial emotion processing in patients with seizure disorders. *Epilepsy & Behavior*, *79*, 193–204. https://doi.org/10.1016/j.yebeh.2017.12.004

Takeuchi, H., Taki, Y., Nouchi, R., Yokoyama, R., Kotozaki, Y., Nakagawa, S., Sekiguchi, A., Iizuka, K., Hanawa, S., Araki, T., Miyauchi, C. M., Sakaki, K., Sassa, Y., Nozawa, T., Ikeda, S., Yokota, S., Daniele, M., & Kawashima, R. (2018). General intelligence is associated with working memory-related brain activity: New evidence from a large sample study. *Brain Structure and Function*, *223*(9), 4243–4258. https://doi.org/10.1007/s00429-018-1747-5

Taylor, S. F., Kornblum, S., Lauber, E. J., Minoshima, S., & Koeppe, R. A. (1997). Isolation of Specific Interference Processing in the Stroop Task: PET Activation Studies. *NeuroImage*, *6*(2), 81–92. https://doi.org/10.1006/nimg.1997.0285

Terry, D. P., Faraco, C. C., Smith, D., Diddams, M. J., Puente, A. N., & Miller, L. S. (2012). Lack of long-term fMRI differences after multiple sports-related concussions. *Brain Injury*, *26*(13-14), 1684–1696. https://doi.org/10.3109/02699052.2012.722259

Thornton, M. A., & Conway, A. R. A. (2013). Working memory for social information: Chunking or domain-specific buffer? *NeuroImage*, *70*, 233–239. https://doi.org/10.1016/j.neuroimage.2012.12.063

Todorov, A., & Engell, A. D. (2008). The role of the amygdala in implicit evaluation of emotionally neutral faces. *Social Cognitive and Affective Neuroscience*, *3*(4), 303–312. https://doi.org/10.1093/scan/nsn033

Trautmann, S. A., Fehr, T., & Herrmann, M. (2009). Emotions in motion: Dynamic compared to static facial expressions of disgust and happiness reveal more widespread emotion-specific activations. *Brain Research*, *1284*, 100–115. https://doi.org/10.1016/j.brainres.2009.05.075

Vacchi, L., Rocca, M. A., Meani, A., Rodegher, M., Martinelli, V., Comi, G., Falini, A., & Filippi, M. (2017). Working memory network dysfunction in relapse-onset multiple sclerosis phenotypes: A clinical-imaging evaluation. *Multiple Sclerosis Journal*, *23*(4), 577–587. https://doi.org/10.1177/1352458516656809

van de Meerendonk, N., Rueschemeyer, S.-A., & Kolk, H. H. J. (2013). Language comprehension interrupted: Both language errors and word degradation activate Broca’s area. *Brain and Language*, *126*(3), 291–301. https://doi.org/10.1016/j.bandl.2013.07.004

van der Horn, H. J., Liemburg, E. J., Scheenen, M. E., de Koning, M. E., Spikman, J. M., & van der Naalt, J. (2016). Post-concussive complaints after mild traumatic brain injury associated with altered brain networks during working memory performance.

Brain Imaging and Behavior, *10*(4), 1243–1253. https://doi.org/10.1007/s11682-015-9489-y

van ‘t Ent, D., den Braber, A., Rotgans, E., de Geus, E. J. C., & de Munck, J. C. (2014). The use of fMRI to detect neural responses to cognitive interference and planning: Evidence for a contribution of task related changes in heart rate? *Journal of Neuroscience Methods*, *229*, 97–107. https://doi.org/10.1016/j.jneumeth.2014.04.013

Van Essen, D. C., Smith, S. M., Barch, D. M., Behrens, T. E. J., Yacoub, E., & Ugurbil, K. (2013). The WU-Minn Human Connectome Project: An overview. *NeuroImage*, *80*, 62–79. https://doi.org/10.1016/j.neuroimage.2013.05.041

Verhallen, A. M., Renken, R. J., Marsman, J.-B. C., & ter Horst, G. J. (2021). Working Memory Alterations After a Romantic Relationship Breakup. *Frontiers in Behavioral Neuroscience*, *15*, 657264. https://doi.org/10.3389/fnbeh.2021.657264

Veroude, K., Jolles, J., Croiset, G., & Krabbendam, L. (2013). Changes in neural mechanisms of cognitive control during the transition from late adolescence to young adulthood. *Developmental Cognitive Neuroscience*, *5*, 63–70. https://doi.org/10.1016/j.dcn.2012.12.002

Verstynen, T. D. (2014). The organization and dynamics of corticostriatal pathways link the medial orbitofrontal cortex to future behavioral responses. *Journal of Neurophysiology*, *112*(10), 2457–2469. https://doi.org/10.1152/jn.00221.2014

Via, E., Cardoner, N., Pujol, J., Alonso, P., López-Solà, M., Real, E., Contreras-Rodríguez, O., Deus, J., Segalàs, C., Menchón, J. M., Soriano-Mas, C., & Harrison, B. J. (2014). Amygdala activation and symptom dimensions in obsessive–compulsive disorder. *British Journal of Psychiatry*, *204*(1), 61–68. https://doi.org/10.1192/bjp.bp.112.123364

Villalta-Gil, V., Hinton, K. E., Landman, B. A., Yvernault, B. C., Perkins, S. F., Katsantonis, A. S., Sellani, C. L., Lahey, B. B., & Zald, D. H. (2017). Convergent individual differences in visual cortices, but not the amygdala across standard

amygdalar fMRI probe tasks. *NeuroImage*, *146*, 312–319. https://doi.org/10.1016/j.neuroimage.2016.11.038

Vuilleumier, P., Armony, J. L., Driver, J., & Dolan, R. J. (2001). Effects of Attention and Emotion on Face Processing in the Human Brain: An Event-Related fMRI Study. *Neuron*, *30*(3), 829–841. https://doi.org/10.1016/S0896-6273(01)00328-2

Vuilleumier, P., Richardson, M. P., Armony, J. L., Driver, J., & Dolan, R. J. (2004). Distant influences of amygdala lesion on visual cortical activation during emotional face processing. *Nature Neuroscience*, *7*(11), 1271–1278. https://doi.org/10.1038/nn1341

Wagner, G., De la Cruz, F., Schachtzabel, C., Güllmar, D., Schultz, C. C., Schlösser, R. G., Bär, K.-J., & Koch, K. (2015). Structural and functional dysconnectivity of the fronto-thalamic system in schizophrenia: A DCM-DTI study. *Cortex*, *66*, 35–45. https://doi.org/10.1016/j.cortex.2015.02.004

Waiter, G. D., Deary, I. J., Staff, R. T., Murray, A. D., Fox, H. C., Starr, J. M., & Whalley, L. J. (2009). Exploring possible neural mechanisms of intelligence differences using processing speed and working memory tasks: An fMRI study. *Intelligence*, *37*(2), 199–206. https://doi.org/10.1016/j.intell.2008.09.008

Walitt, B., Čeko, M., Khatiwada, M., Gracely, J. L., Rayhan, R., VanMeter, J. W., & Gracely, R. H. (2016). Characterizing “fibrofog”: Subjective appraisal, objective performance, and task-related brain activity during a working memory task. *NeuroImage: Clinical*, *11*, 173–180. https://doi.org/10.1016/j.nicl.2016.01.021

Wallentin, M., Gravholt, C. H., & Skakkebæk, A. (2015). Broca’s region and Visual Word Form Area activation differ during a predictive Stroop task. *Cortex*, *73*, 257–270. https://doi.org/10.1016/j.cortex.2015.08.023

Weisenbach, S. L., Rapport, L. J., Briceno, E. M., Haase, B. D., Vederman, A. C., Bieliauskas, L. A., Welsh, R. C., Starkman, M. N., McInnis, M. G., Zubieta, J.-K., & Langenecker, S. A. (2014). Reduced emotion processing efficiency in healthy males

relative to females. *Social Cognitive and Affective Neuroscience*, *9*(3), 316–325. https://doi.org/10.1093/scan/nss137

Wesley, M. J., Lile, J. A., Fillmore, M. T., & Porrino, L. J. (2017). Neurophysiological capacity in a working memory task differentiates dependent from nondependent heavy drinkers and controls. *Drug and Alcohol Dependence*, *175*, 24–35. https://doi.org/10.1016/j.drugalcdep.2017.01.029

Williams, L. M., Brown, K. J., Das, P., Boucsein, W., Sokolov, E. N., Brammer, M. J., Olivieri, G., Peduto, A., & Gordon, E. (2004). The dynamics of cortico-amygdala and autonomic activity over the experimental time course of fear perception. *Cognitive Brain Research*, *21*(1), 114–123. https://doi.org/10.1016/j.cogbrainres.2004.06.005

Williams, L. M., Das, P., Harris, A. W. F., Liddell, B. B., Brammer, M. J., Olivieri, G., Skerrett, D., Phillips, M. L., David, A. S., Peduto, A., & Gordon, E. (2004). Dysregulation of Arousal and Amygdala-Prefrontal Systems in Paranoid Schizophrenia. *American Journal of Psychiatry*, *161*(3), 480–489. https://doi.org/10.1176/appi.ajp.161.3.480

Williams, L. M., Das, P., Liddell, B., Olivieri, G., Peduto, A., Brammer, M. J., & Gordon, E. (2005). BOLD, sweat and fears: fMRI and skin conductance distinguish facial fear signals: *NeuroReport*, *16*(1), 49–52. https://doi.org/10.1097/00001756-200501190-00012

Williams, L. M., Kemp, A. H., Felmingham, K., Barton, M., Olivieri, G., Peduto, A., Gordon, E., & Bryant, R. A. (2006). Trauma modulates amygdala and medial prefrontal responses to consciously attended fear. *NeuroImage*, *29*(2), 347–357. https://doi.org/10.1016/j.neuroimage.2005.03.047

Williams, L. M., Phillips, M. L., Brammer, M. J., Skerrett, D., Lagopoulos, J., Rennie, C., Bahramali, H., Olivieri, G., David, A. S., Peduto, A., & Gordon, E. (2001). Arousal Dissociates Amygdala and Hippocampal Fear Responses: Evidence from Simultaneous fMRI and Skin Conductance Recording. *NeuroImage*, *14*(5), 1070–1079. https://doi.org/10.1006/nimg.2001.0904

Wishart, H. A., Saykin, A. J., Rabin, L. A., Santulli, R. B., Flashman, L. A., Guerin, S. J., Mamourian, A. C., Belloni, D. R., Rhodes, C. H., & McAllister, T. W. (2006). Increased Brain Activation During Working Memory in Cognitively Intact Adults With the APOE $E4$ Allele. *American Journal of Psychiatry*, *163*(9), 1603–1610. https://doi.org/10.1176/ajp.2006.163.9.1603

Woolrich, M. W., Behrens, T. E. J., Beckmann, C. F., Jenkinson, M., & Smith, S. M. (2004). Multilevel linear modelling for FMRI group analysis using Bayesian inference. *NeuroImage*, *21*(4), 1732–1747. https://doi.org/10.1016/j.neuroimage.2003.12.023

Woolrich, M. W., Ripley, B. D., Brady, M., & Smith, S. M. (2001). Temporal Autocorrelation in Univariate Linear Modeling of FMRI Data. *NeuroImage*, *14*(6), 1370–1386. https://doi.org/10.1006/nimg.2001.0931

Wright, P., & Liu, Y. (2006). Neutral faces activate the amygdala during identity matching. *NeuroImage*, *29*(2), 628–636. https://doi.org/10.1016/j.neuroimage.2005.07.047

Wu, S., Wang, H., Chen, C., Zou, J., Huang, H., Li, P., Zhao, Y., Xu, Q., Zhang, L., Wang, H., Pandit, S., Dahal, S., Chen, J., Zhou, Y., Jiang, T., & Wang, G. (2017). Task Performance Modulates Functional Connectivity Involving the Dorsolateral Prefrontal Cortex in Patients with Schizophrenia. *Frontiers in Psychology*, *8*. https://doi.org/10.3389/fpsyg.2017.00056

Yan, X., Zhang, J., Gong, Q., & Weng, X. (2011). Adaptive influence of long term high altitude residence on spatial working memory: An fMRI study. *Brain and Cognition*, *77*(1), 53–59. https://doi.org/10.1016/j.bandc.2011.06.002

Yang, X., Zhang, X., Yang, Y., & Lin, N. (2018). How context features modulate the involvement of the working memory system during discourse comprehension. *Neuropsychologia*, *111*, 36–44. https://doi.org/10.1016/j.neuropsychologia.2018.01.010

Yaple, Z. A., Stevens, W. D., & Arsalidou, M. (2019). Meta-analyses of the n-back working memory task: fMRI evidence of age-related changes in prefrontal cortex

involvement across the adult lifespan. *NeuroImage*, *196*, 16–31. https://doi.org/10.1016/j.neuroimage.2019.03.074

Ye, Z., & Zhou, X. (2009). Conflict control during sentence comprehension: fMRI evidence. *NeuroImage*, *48*(1), 280–290. https://doi.org/10.1016/j.neuroimage.2009.06.032

Yoo, S.-S., Choi, B.-G., Juh, R.-H., Park, J.-M., Pae, C.-U., Kim, J.-J., Lee, S.-J., Lee, C., Paik, I.-H., Lee, C.-U., & Adkinson, N. F. (2005). WORKING MEMORY PROCESSING OF FACIAL IMAGES IN SCHIZOPHRENIA: fMRI INVESTIGATION. *International Journal of Neuroscience*, *115*(3), 351–366. https://doi.org/10.1080/00207450590520957

Zhang, Z., Peng, P., Eickhoff, S. B., Lin, X., Zhang, D., & Wang, Y. (2021). Neural substrates of the executive function construct, age-related changes, and task materials in adolescents and adults: ALE meta-analyses of 408 fMRI studies. *Developmental Science*, desc.13111. https://doi.org/10.1111/desc.13111

Zhu, Z., Feng, G., Zhang, J. X., Li, G., Li, H., & Wang, S. (2013). The role of the left prefrontal cortex in sentence-level semantic integration. *NeuroImage*, *76*, 325–331. https://doi.org/10.1016/j.neuroimage.2013.02.060

Ziemus, B., Baumann, O., Luerding, R., Schlosser, R., Schuierer, G., Bogdahn, U., & Greenlee, M. (2007). Impaired working-memory after cerebellar infarcts paralleled by changes in BOLD signal of a cortico-cerebellar circuit. *Neuropsychologia*, *45*(9), 2016–2024. https://doi.org/10.1016/j.neuropsychologia.2007.02.012

Zoccatelli, G., Beltramello, A., Alessandrini, F., Pizzini, F. B., & Tassinari, G. (2010). Word and position interference in stroop tasks: A behavioral and fMRI study. *Experimental Brain Research*, *207*(1-2), 139–147. https://doi.org/10.1007/s00221-010-2433-x

Zsoldos, I., Cousin, E., Klein-Koerkamp, Y., Pichat, C., & Hot, P. (2016). Age-related differences in brain activity during implicit and explicit processing of fearful facial

expressions. *Brain Research*, *1650*, 208–217. https://doi.org/10.1016/j.brainres.2016.09.004

Zurowski, B., Gostomzyk, J., Grön, G., Weller, R., Schirrmeister, H., Neumeier, B., Spitzer, M., Reske, S. N., & Walter, H. (2002). Dissociating a Common Working Memory Network from Different Neural Substrates of Phonological and Spatial Stimulus Processing. *NeuroImage*, *15*(1), 45–57. https://doi.org/10.1006/nimg.2001.0968

Zysset, S., Müller, K., Lohmann, G., & von Cramon, D. Y. (2001). Color-Word Matching Stroop Task: Separating Interference and Response Conflict. *NeuroImage*, *13*(1), 29–36. https://doi.org/10.1006/nimg.2000.0665

Zysset, S., Schroeter, M. L., Neumann, J., & Yves von Cramon, D. (2007). Stroop interference, hemodynamic response and aging: An event-related fMRI study. *Neurobiology of Aging*, *28*(6), 937–946. https://doi.org/10.1016/j.neurobiolaging.2006.05.008
